# Vascular smooth muscle RbFox2 regulates the cytoskeleton and arterial stiffness by a RhoBTB1/Cullin-3 mechanism

**DOI:** 10.1172/jci.insight.202638

**Published:** 2026-04-02

**Authors:** Gaurav Kumar, Nisita Chaihongsa, Daniel T. Brozoski, Daria Golosova, Ibrahim Vazirabad, Ko-Ting Lu, Kelsey K. Wackman, Ravi K. Singh, Curt D. Sigmund

**Affiliations:** 1 Department of Physiology, Cardiovascular Center, Medical College of Wisconsin, Milwaukee, Wisconsin, USA.; 2 Department of Pharmacological and Pharmaceutical Sciences, College of Pharmacy, University of Houston, Houston, Texas, USA.

**Keywords:** Cardiology, Vascular biology, Hypertension, Mouse models, Proteomics

## Abstract

The RhoBTB1/Cullin-3 (CUL3) pathway in smooth muscle cells (SMCs) controls the ubiquitination and proteasomal degradation of target proteins that regulate vasodilation, vasoconstriction, and the actin cytoskeleton and, through this, blood pressure (BP) and arterial stiffness. Using proximity labeling coupled with mass spectrometry in A7R5 SMCs, we identified proteins that bound to the C-terminal half of RhoBTB1, which functions as an adaptor to deliver substrates to CUL3. We examined the physiological relevance of one of these substrates, RbFox2. Coimmunoprecipitation validated the interaction of RbFox2 with RhoBTB1. RbFox2 expression was elevated in response to inhibition of the ubiquitination-proteasomal pathway, CUL3 deficiency, and RhoBTB1 inhibition by either siRNA or angiotensin II (ANG). RbFox2 was ubiquitinated in a RhoBTB1- and CUL3-dependent manner, suggesting its regulation through the RhoBTB1/CUL3-dependent ubiquitin-proteasome pathway. Inhibition of RbFox2 impaired the actin cytoskeleton in A7R5 cells and in primary SMCs from *RbFox2^fl/fl^* mice and decreased the levels of globular and filamentous actin. ANG increased BP and arterial stiffness of *RbFox2^fl/fl^* mice, but the progression of arterial stiffness was halted after SMC-specific RbFox2 deletion despite a continued rise in BP. We conclude that RhoBTB1 and RbFox2 are important regulators of arterial stiffness through a mechanism that influences cytoskeletal integrity.

## Abstract 2

RbFox2 is a RhoBTB1 target controlled by the CUL3 E3-ubiquitin ligase which plays a role in arterial stiffness by regulating components of the actin cytoskeleton.

## Graphical abstract

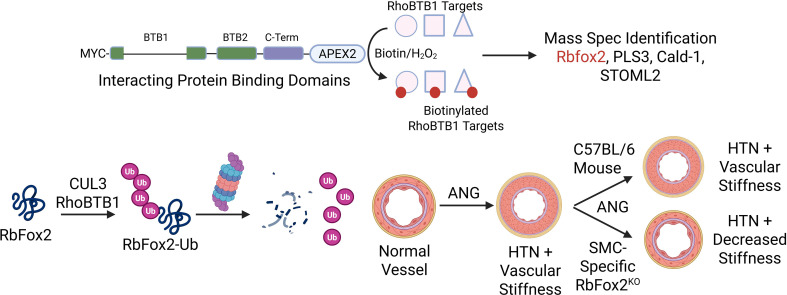

## Introduction

Cardiovascular diseases (CVDs) are a leading cause of mortality in the United States. The economic impact of CVDs is expected to dramatically rise in the coming decades. The American Heart Association estimates that the treatment of CVDs will cost more than $1 trillion by 2035 (1). Hypertension (HTN) is a risk factor for the initiation and progression of endothelial dysfunction and arterial stiffness, both of which are CVD risk factors (2). Arterial stiffness results from structural changes that promote adverse vascular remodeling, ultimately contributing to loss in vascular elasticity (3). It amplifies the wall stress induced by HTN and other factors, including oxidative stress, vascular inflammation, and renin-angiotensin system activation, and can result in a progressive worsening of arterial stiffness. These challenges trigger rearrangement and structural changes in the vascular cell cytoskeleton such as actin and its associated proteins (4). While initially adaptive, these changes may become maladaptive under chronic pathological conditions, driving their effects into progression of vascular rigidity and poorly compliant vessels, typically observed in chronic HTN.

PPARγ is a nuclear transcriptional factor and important regulator of vasomotor function (5). In endothelium, PPARγ controls the bioavailability of nitric oxide (NO), whereas in vascular smooth muscle it controls the response to endothelium-derived NO (6). One of the downstream targets of PPARγ is RhoBTB1, an atypical GTPase that functions as an adaptor for Cullin-3 (CUL3), a scaffolding component of the Cullin–RING (really *i*nteresting new gene) E3 ubiquitin ligase complex (7). Both RhoBTB1 and CUL3 are important regulators of vasomotor tone (8). *RhoBTB1* is in a locus genetically associated with blood pressure (BP) (9, 10). Mice expressing a smooth muscle cell–specific (SMC-specific) loss-of-function PPARγ mutation exhibit RhoBTB1 deficiency and HTN, profoundly impaired vascular function, and arterial stiffness (7, 11). SMC-specific restoration of RhoBTB1 expression in mice carrying an SMC-specific dominant-negative loss-of-function PPARγ mutation reversed the adverse cardiovascular phenotype, including elevated BP and arterial stiffness (12). RhoBTB1 regulates the cellular levels of phosphodiesterase 5 (PDE5) and, through this, the levels of cGMP and, consequently, NO bioavailability. Mechanistically, it accomplishes this by being a substrate recognition protein that delivers PDE5 to the CUL3–RING E3 ubiquitin ligase complex so it can be degraded by the proteasome. A deficiency in RhoBTB1 decreases PDE5 degradation, which increases PDE5 levels and enzyme activity, thus enhancing the breakdown of cGMP to GMP, resulting in a loss of NO action in SMCs.

RhoBTB1 has multiple domains (GTPase, proline rich, BTB1, BTB2, and C-terminal) (Supplemental Figure 1A; supplemental material available online with this article; https://doi.org/10.1172/jci.insight.202638DS1). The C-terminal half of the protein, consisting of BTB1, BTB2, and the C-terminal (also called B1B2C), is sufficient for its adaptor function (13). Loss of the GTPase and proline-rich domains is dispensable for its adaptor function and may not be involved in regulating vascular function. We reported that angiotensin II (ANG), likely through PPARγ inhibition, downregulates RhoBTB1 (14). Restoration of SMC RhoBTB1 in a model of ANG HTN regresses arterial stiffness by modulating the actin cytoskeleton. Moreover, this occurs independent of PDE5, suggesting other substrates for RhoBTB1 in vascular SMCs. Consequently, the mechanisms by which RhoBTB1 influences arterial stiffness need further investigation.

We used a proximity labeling approach to identify RhoBTB1 target proteins involved in arterial function by fusing the B1B2C domains of RhoBTB1 to ascorbate peroxidase 2 (APEX2) in A7R5 cells followed by mass spectrometry (MS) (Supplemental Figure 1B). Several previously unknown RhoBTB1 binding proteins were identified that may regulate contractile machinery in SMCs. We focused our attention on RNA-binding Fox-1 homolog 2 (RbFox2), a well-characterized splicing factor (15–17). We provide evidence that RbFox2 is regulated by the RhoBTB1/CUL3 pathway and plays an integral role in the regulation of arterial stiffness through effects on the actin cytoskeleton in SMCs and by modulating mRNA splicing, particularly of mRNAs encoding cytoskeletal proteins. These findings and our collective data support the notion that RbFox2 could be a potential therapeutic target for arterial stiffness.

## Results

### APEX2 proximity labeling identifies the RhoBTB1-interacting proteome.

We employed APEX2 and an MS-assisted proximity labeling approach to identify RhoBTB1 binding proteins in the A7R5 SMC line. We used a fusion construct comprising the C-terminal B1B2C domains of RhoBTB1 fused to APEX2 (Supplemental Figure 1). The B1B2C domains of RhoBTB1 are the minimal region necessary and sufficient to bind to PDE5 and foster its ubiquitination (13). APEX2 proximity labeling depends on the distance traveled by phenoxyl free radicals and biotinylates proteins within a 20 nm radius (18). To control for excess labeling of the A7R5 SMC proteome by B1B2C-APEX2, we used a concurrent control screen composed of a B1B2-APEX2 fusion protein that lacks the C-terminus of RhoBTB1. The premise is based on our previous study demonstrating that RhoBTB1 domains lacking the C-terminus could not bind to RhoBTB1-CUL3 substrates (13). Comparing the proteins identified through APEX2 proximity labeling of the SMC proteome using B1B2C-APEX2 versus B1B2-APEX2 should allow us to enrich the biotinylated proteome interacting with the C-terminal half of RhoBTB1.

We validated the expression of B1B2-APEX2 and B1B2C-APEX2 in A7R5 SMCs by immunoblotting (Supplemental Figure 2). Transfected A7R5 cells were treated with biotin phenol followed by a flash exposure of H_2_O_2_ to induce biotinylation (13). Immunoblotting with streptavidin-HRP conjugate demonstrated robust biotinylation of multiple proteins only in the presence of all reaction components, confirming the functionality of the assay (Supplemental Figure 3). Our previous data showing a preservation of B1B2C-APEX2 binding to PDE5 suggested that the APEX2 tag does not disturb the interaction of RhoBTB1 with its binding partners and that APEX2-tagged constructs are suitable for enriching the biotinylated proteome of SMCs (13).

**Figure 1:**
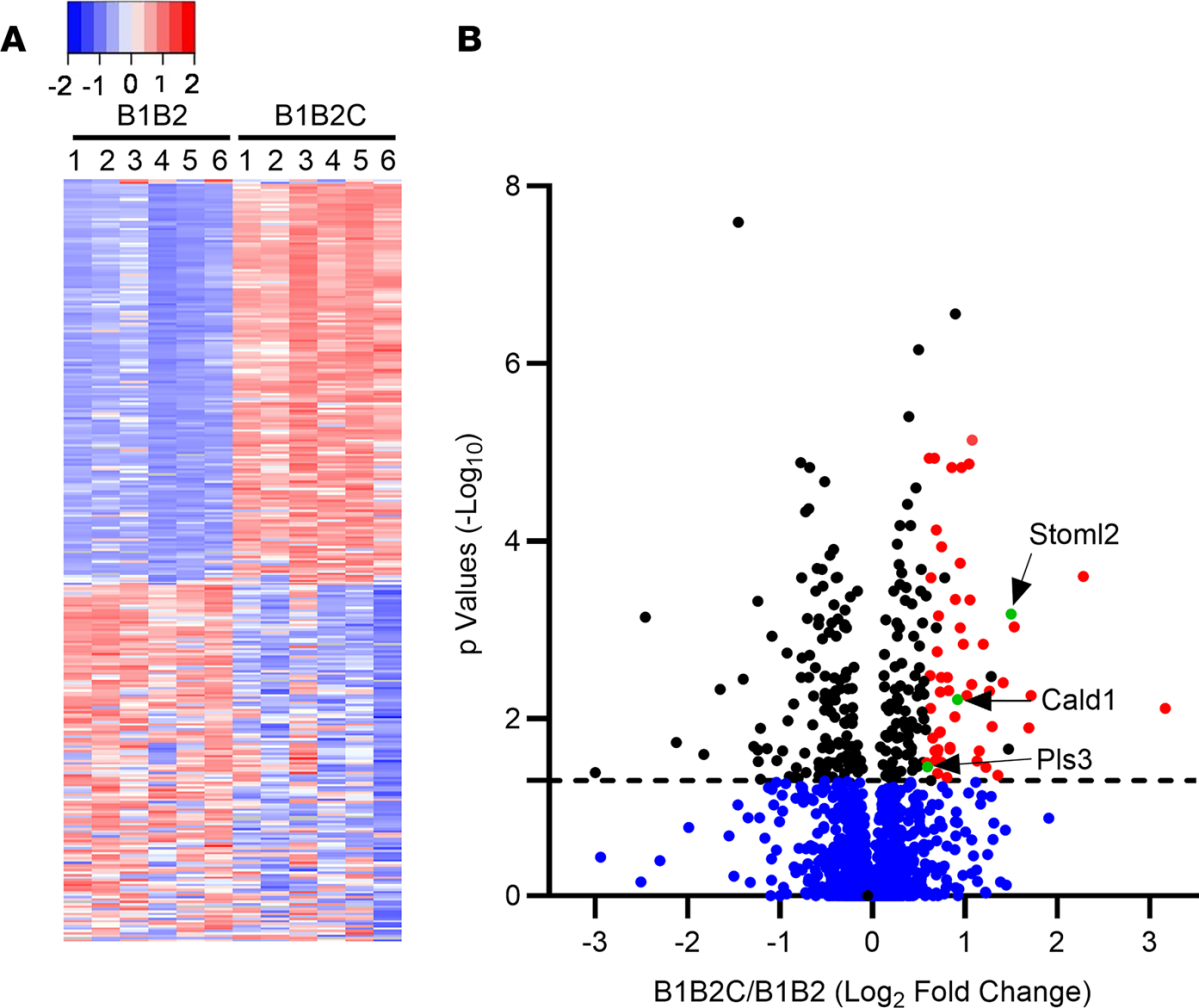
Profiling of the A7R5 proteome. (**A**) Heatmap and hierarchical clustering of RhoBTB1-interacting A7R5 SMC differential proteome identified by mass spectrometry. *N* = 6 (3 technical of 2 biological replicates) per group (B1B2C vs. B1B2). (**B**) Volcano plot of proteomic data. The magnitude of change (log_2_ fold change) is plotted on the *x* axis against the statistical significance (–log_10_) on the *y* axis. Proteins highlighted in red represent higher interaction toward B1B2C. Proteins highlighted in green were validated for differential interaction B1B2C > B1B2. Proteins highlighted in blue represent those below statistical significance.

The biotinylated proteome was enriched by streptavidin-conjugated Dynabeads and subsequently identified using MS. Across 12 total samples (3 technical replicates each of 2 biological samples for B1B2 and B1B2C, respectively), we identified a total of 1,645 total proteins. We selected 315 statistically significant differentially interacting proteins by hierarchical clustering (Benjamini-Hochberg *P* < 0.05; Figure 1A and Supplemental Dataset 1). Setting the relative fold enrichment of proteins comparing B1B2C with B1B2 to 1.5-fold or greater resulted in a list of protein candidates for further analysis (Supplemental Table 1). A volcano plot displays the proteins with higher interaction toward B1B2C (red), proteins validated for their differential interaction with B1B2C and B1B2 (green), and proteins below statistical significance across the proteomic screen (blue; Figure 1B). The candidate proteins were further analyzed for their molecular function and pathway enrichment using ShinyGO (19). Interestingly, many of the proteins in our dataset were related to the actin-based cytoskeleton and smooth muscle contraction, while some were also found to be associated with calcium and calmodulin signaling and RNA binding (Supplemental Figure 4). These findings are consistent with our previous results that revealed that RhoBTB1 regulates arterial stiffness through the actin cytoskeleton (14).

### Validation of RhoBTB1 protein targets.

**Figure 2:**
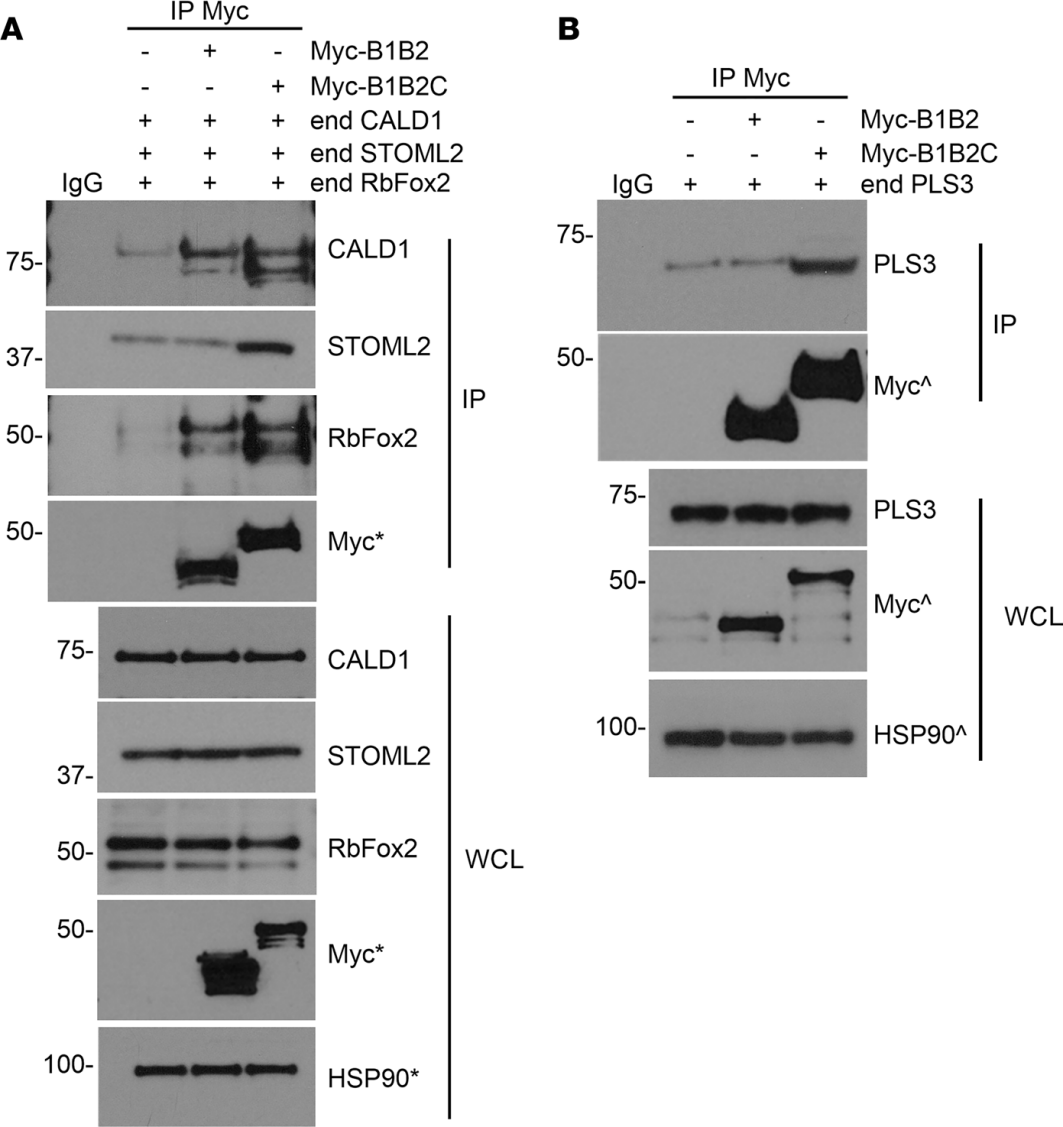
Differential interaction of RhoBTB1 target proteins with RhoBTB1 domains. Co-IP assay was performed with Myc-tagged RhoBTB1 domains (B1B2C and B1B2) transfected in A7R5 SMCs using anti-Myc beads or isotype-matched IgG as control. Cells in the control lane were transfected with pcDNA3.1 empty vector. Cells were treated with MLN4924 for 16 hours and MG132 for the last 4 hours. Transfection time was 24 hours. Immunocomplexes were Western blotted with indicated antisera. Molecular weight markers are transferred from original blots. IP, immunoprecipitates; WCL, whole-cell lysates. Data are representative of 2 independent replicates. Hsp90 was used as an internal control. *^,^^These blots are identical to those in Supplemental Figure 5, A and B, as those blots were from the same contemporaneous experiment.

Proximity labeling can result in nonspecific labeling and affinity capture of nearby proteins that do not directly bind to the bait protein. Thus, we validated the interactions of RhoBTB1 with several top-ranked candidate proteins — stomatin like 2 (StomL2), filamin A (Flna), tropomyosin 2 (Tpm2), caldesmon 1 (Cald1), plastin 3 (Pls3), and Ras homolog family member Q (RhoQ) — via coimmunoprecipitation (co-IP). A7R5 cells were transfected with either B1B2 or B1B2C domains of RhoBTB1 (this time lacking the APEX2 fusion) for 24 hours, followed by co-IP to assess physical interactions. Notably, Cald1, StomL2, and Pls3 exhibited stronger interaction with B1B2C than with B1B2 (Figure 2, and B). Conversely, despite having higher interaction score in the biotinylation screen, Flna, Tpm2, and RhoQ did not interact with B1B2C in co-IP assays (Supplemental Figure 5, A and B).

**Figure 3:**
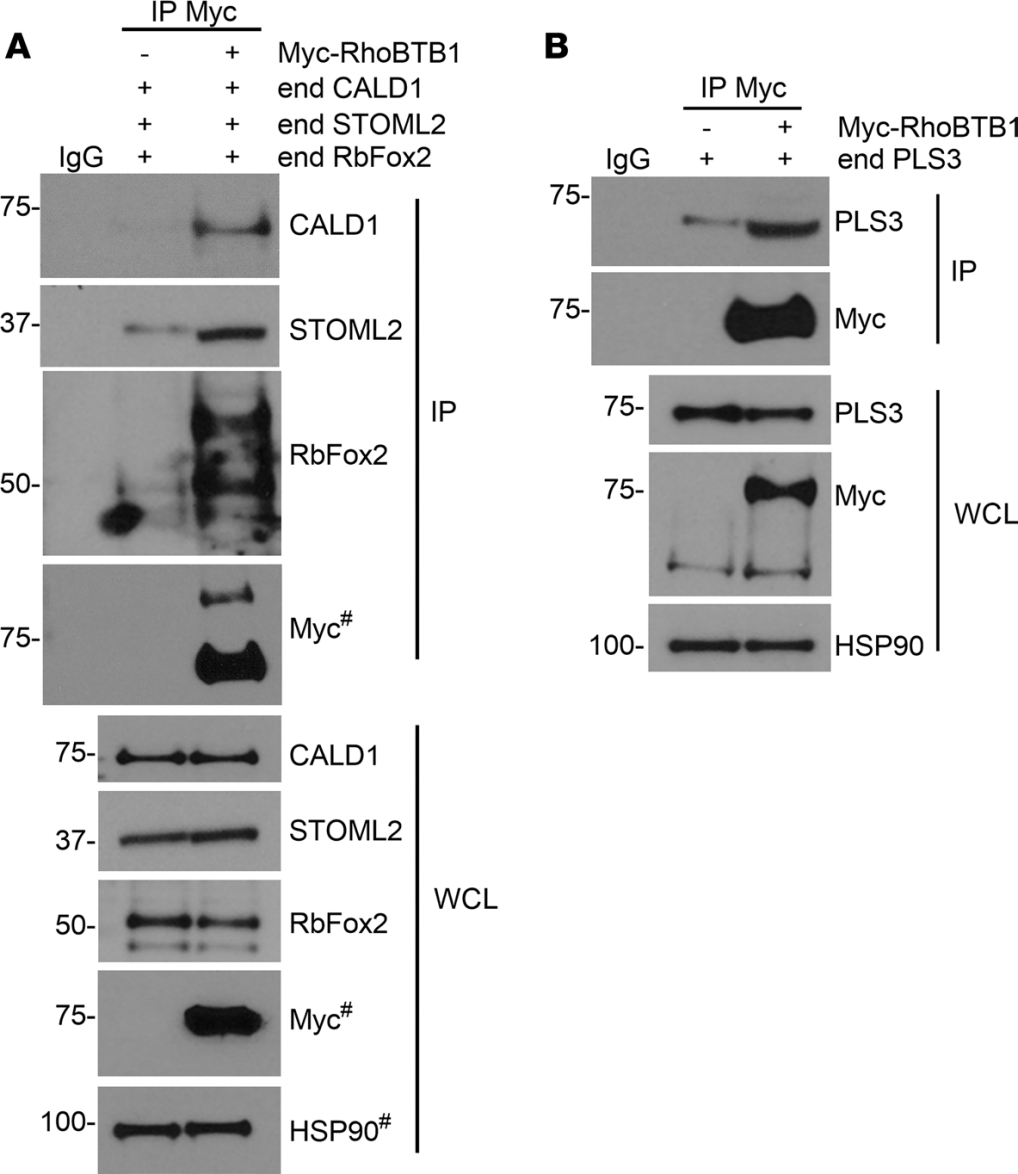
Differential interaction of RhoBTB1 target proteins with full-length RhoBTB1. Co-IP assay was performed in Myc epitope–tagged full-length RhoBTB1–transfected A7R5 SMCs using anti-Myc beads or isotype-matched IgG as control. Cells in the control lane were transfected with pcDNA3.1 empty vector. Cells were treated with MLN4924 for 16 hours and MG132 for the last 4 hours. Transfection time was 24 hours. Immunocomplexes were Western blotted with indicated antisera. Hsp90 was used as loading control. Molecular weight markers were transferred from original blots. Data are representative of 2 independent replicates. ^#^These blots are identical to those in Supplemental Figure 5C, as those blots were from the same contemporaneous experiment.

We next sought to determine whether the identified proteins interact with full-length RhoBTB1. To this end, A7R5 SMCs were transfected with Myc epitope–tagged full-length RhoBTB1, and the whole-cell lysates were subjected to co-IP. Consistent with the domain-specific co-IP results, Cald1, StomL2, and Pls3 were confirmed to interact with full-length RhoBTB1 (Figure 3, A and B). As before, RhoQ was not detected in the immune complex with full-length RhoBTB1 (Supplemental Figure 5C). These results validate that a subset of the candidate proteins detected through the proteomic screen specifically interact with full-length RhoBTB1. This also supports the idea that RhoBTB1 may selectively scaffold or regulate distinct protein complexes in SMCs.

In addition to the proteins selected above, we also chose to examine whether RNA-binding Fox-1 homolog 2 (RbFox2) also associated with the B1B2C domain and full-length RhoBTB1 (15). This choice was made for two reasons. First, the proteomic data demonstrated association of RbFox2 with B1B2C in 3 of 6 replicates, but no RbFox2 association with B1B2 in any replicates (Supplemental Dataset 1). Second, we had access to a readily available conditional knockout model for *RbFox2^flox/flox^* (*RbFox2^fl/fl^*) that offered us an opportunity to rapidly explore its regulation by the RhoBTB1-CUL3 axis and its potential role in vivo. To validate RhoBTB1 interaction with RbFox2, we performed co-IP with B1B2C and full-length RhoBTB1, which showed a stronger interaction between RbFox2 and B1B2C compared with B1B2 (Figure 2A) and with full-length RhoBTB1 (Figure 3A). Thus, we conclude that Cald1, Pls3, StomL2, and RbFox2 interacted differentially with B1B2 and B1B2C domains of RhoBTB1 and bound to full-length RhoBTB1.

**Figure 4:**
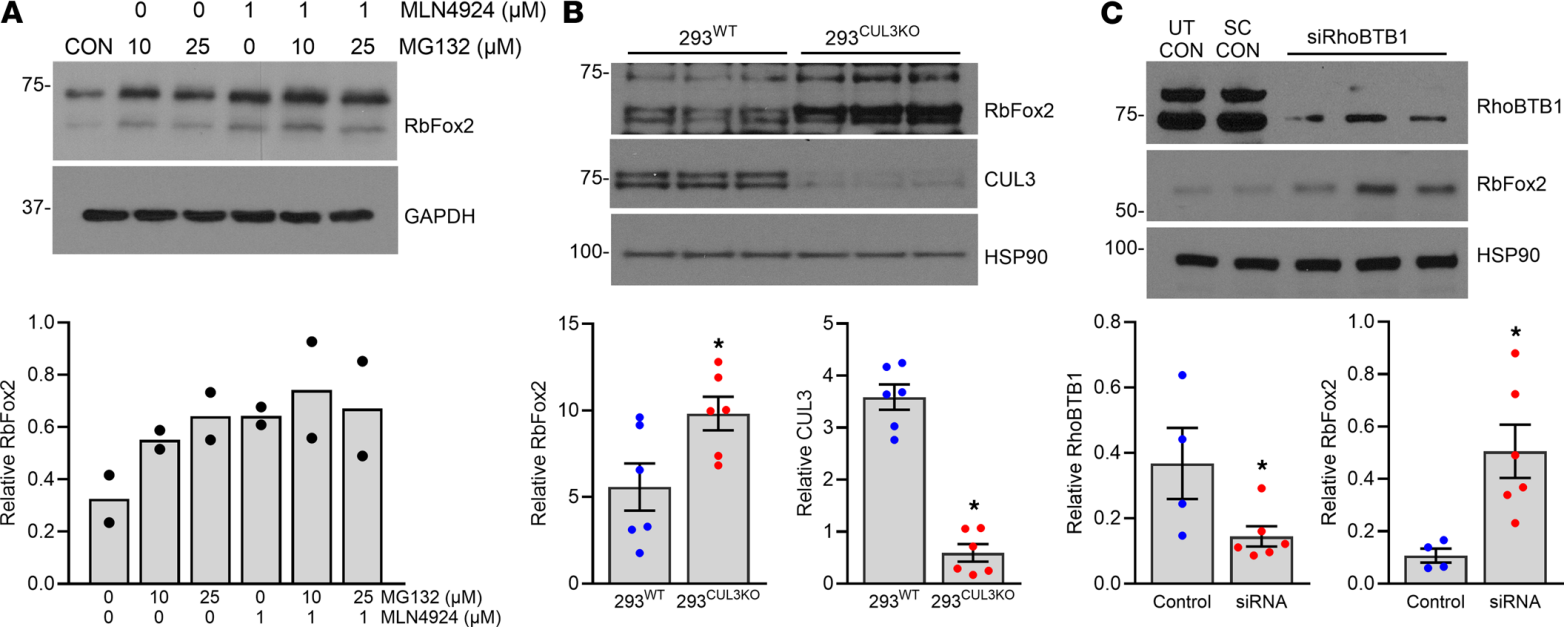
Validation of RbFox2 as a target of the RhoBTB1/CUL3 proteasomal pathway. (**A**) Immunoblot demonstrating the regulation of RbFox2 by CUL3-dependent ubiquitin-proteasome pathway in A7R5 cells treated with MLN4924 alone or in combination with MG132 for 24 hours. GAPDH was a protein loading control. The experiment was performed in duplicate with independent samples and is quantified below. (**B**) Relative levels of RbFox2 and CUL3 in HEK293^WT^ and HEK293^CUL3KO^ cells. Hsp90 was used an internal control. Data are quantified below. Data represent mean ± SEM; *P < 0.05 by 2-tailed *t* test; *N* = 6 each. (**C**) Relative levels of RbFox2 and RhoBTB1 in A7R5 cells transfected with scrambled control (SC CON) and siRNA targeting RhoBTB1 (siRhoBTB1) for 72 hours. UT, untransfected cells. Hsp90 was used as an internal control for protein loading. Data are quantified below and represent mean ± SEM; *P < 0.05 by 2-tailed *t* test; *N* = 4 for control scrambled siRNA; *N* = 6 for RhoBTB1 siRNA. Immunoblots were probed with indicated antisera. Molecular weight markers were transferred from the original blots.

**Figure 5:**
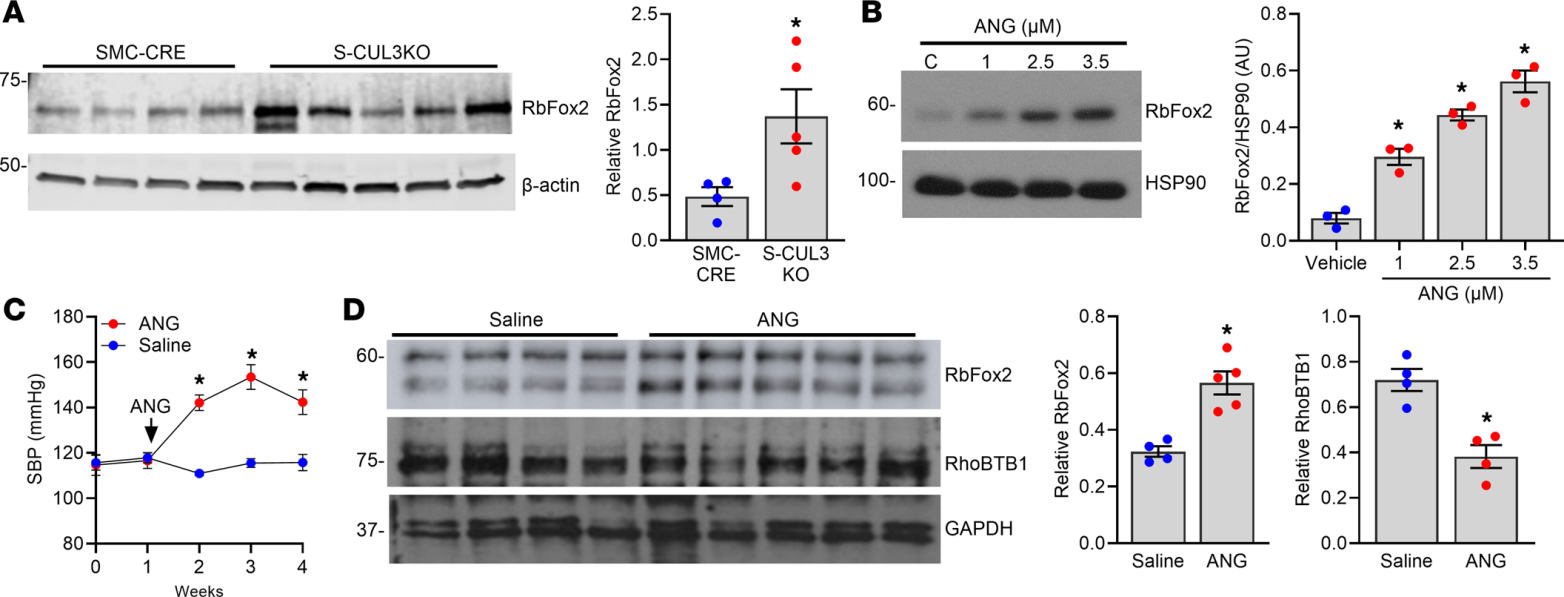
Expression of RbFox2 in various models of hypertension. (**A**) Representative Western blot measuring the expression of RbFox2 in aorta from SMC-CRE and S-CUL3KO mice. Relative levels of RbFox2 in SMC-CRE and S-CUL3KO mouse aorta are quantified. Data represent mean ± SEM; *P < 0.05 by 2-tailed *t* test; *N* = 4 for SMC-CRE; *N* = 5 for S-CUL3KO. These Western blot data are from samples from a previously published study (44). RbFox2 is a re-probe of a CUL3 Western blot from that study, whereas the β-actin blot is the same. (**B**) Immunoblot measuring the expression of RbFox2 in response to dose-dependent treatment of A7R5 cells with ANG (indicated in μM). Data are quantified and represent mean ± SEM; *P < 0.05 by 1-way ANOVA with Dunnett’s multiple comparisons test; representative of *N* = 3 for all samples. (**C**) Summary data (mean ± SEM) for SBP (mmHg) at baseline and after ANG-induced hypertension (1,000 ng/min/kg, Alzet minipump) in C57BL/6 mice for indicated times measured in weeks. *P < 0.05 by 2-way repeated-measures ANOVA with Tukey’s multiple comparison test vs. 0 weeks (baseline); *N* = 6 for both ANG and saline. One mouse died after the second week of ANG. (**D**) Immunoblot showing RbFox2 expression in aorta from some of the saline- and ANG-treated C57BL/6 mice from **C**. Data are quantified and represent mean ± SEM; *P < 0.05 by 2-tailed *t* test; *N* = 4 for saline; *N* = 5 for ANG. One sample in the RhoBTB1 ANG group was found to be an outlier by Grubb’s test. Molecular weight markers were transferred from the original blots.

RhoBTB1 serves as a substrate adaptor for CUL3, a scaffold protein that plays a key role in the ubiquitin-proteasome pathway (6). The co-IP data led us to hypothesize that the cellular expression of RbFox2 might be regulated through the RhoBTB1/CUL3 axis. We investigated whether the expression of RbFox2 is regulated via the ubiquitin-proteasome pathway through RhoBTB1- and CUL3-dependent mechanisms. First, pharmacological inhibition of the proteasome with MG132 or inhibition of Cullin neddylation with MLN4924 increased RbFox2 protein in A7R5 cells (Figure 4A). Neddylation of Cullin proteins is required for their activity (20). Second, the expression of RbFox2 was elevated in CRISPR/Cas9–edited CUL3-deficient HEK293 cells (referred to as HEK293^CUL3KO^ hereafter) compared with the parental HEK293 wild-type cells (HEK293^WT^) (Figure 4B) (21). Third, RbFox2 expression was increased in A7R5 cells transfected with a siRNA targeting *RhoBTB1* (Figure 4C). Like in the HEK293^CUL3KO^ cells, expression of RbFox2 was increased in aorta from mice with SMC-specific CUL3 knockout (termed S-CUL3KO) (Figure 5A) (22). Additionally, treatment of A7R5 SMCs with angiotensin II (ANG), which we previously showed markedly downregulated RhoBTB1 (14), also led to a dose-dependent elevation in RbFox2 expression (Figure 5B). Similarly, ANG treatment of C57BL/6 mice that caused a marked increase in systolic BP (SBP) (Figure 5C) was accompanied by a corresponding decrease in expression of RhoBTB1 and increase in expression of RbFox2 in the aorta in comparison with the saline-treated control group (Figure 5D).

**Figure 6:**
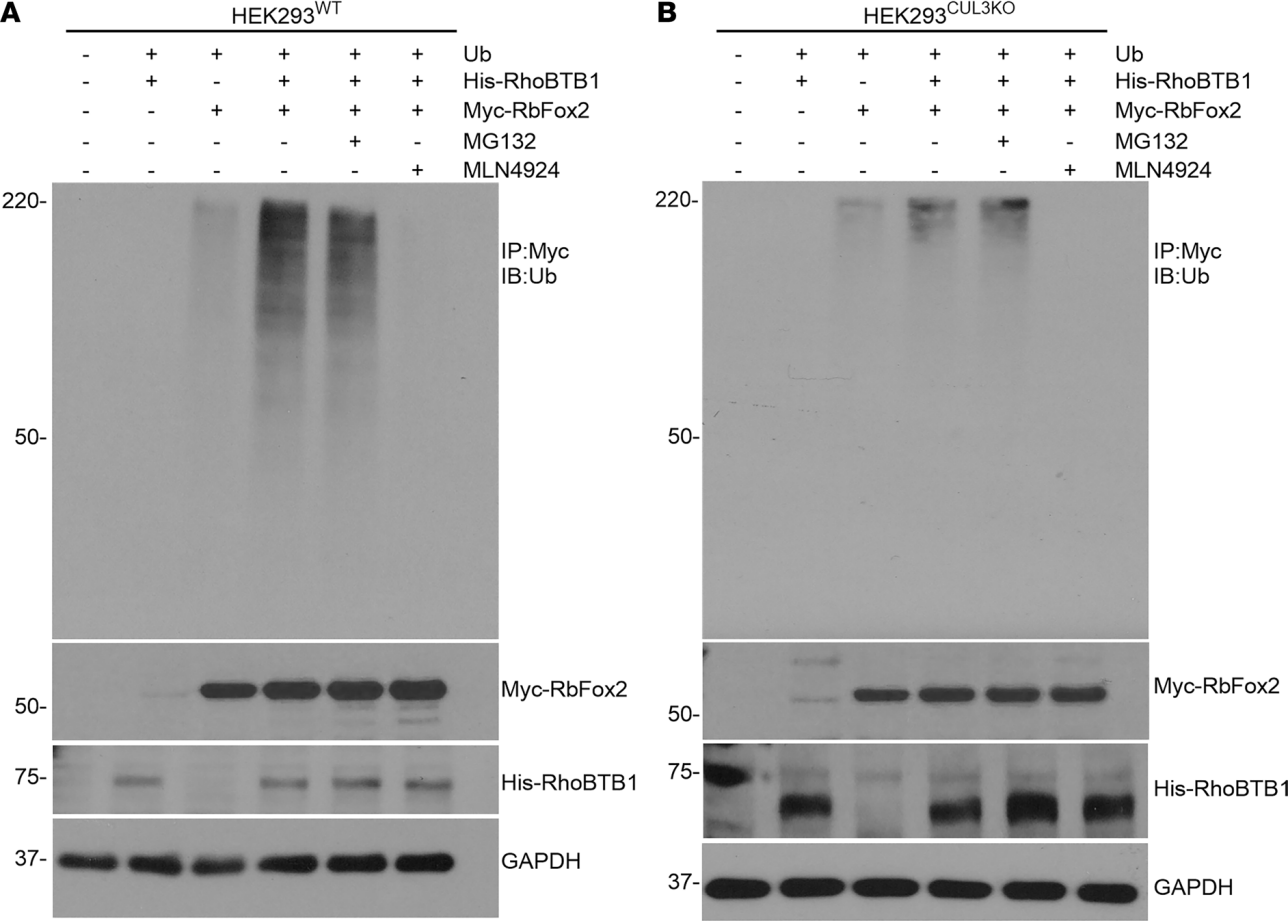
RhoBTB1-dependent polyubiquitination of RbFox2. Representative immunoblot showing polyubiquitination observed in wild-type HEK293 cells (HEK293^WT^, **A**) and CRISPR/Cas9–edited CUL3-knockout HEK293 cells (HEK293^CUL3KO^, **B**) transfected with the indicated His-tagged RhoBTB1, Myc-tagged RbFox2, and ubiquitin constructs. MG132 and MLN4924 treatment was started immediately after transfection of the cells. Whole-cell lysates were collected, and IP was performed under denaturing conditions that prevent RhoBTB1 from getting pulled down. Immune complexes were probed with the indicated antisera. The top blots are the IP, and the bottom 3 blots are from whole-cell lysates. Molecular weight markers were transferred from the original blots. GAPDH was used as an internal control. These data are representative of 2 independent experiments.

BTB domain–containing proteins can bind to their substrate and mediate polyubiquitination, thereby targeting them for degradation via the cullin ring ligases (CRL) complex–dependent proteasomal pathway (23, 24). Therefore, we investigated whether RbFox2 degradation occurs through ubiquitination. Myc-tagged RbFox2 was cotransfected with ubiquitin either with an empty vector or with His-tagged RhoBTB1 in HEK293^WT^ cells (Figure 6A). This revealed ubiquitination of RbFox2 in the presence of RhoBTB1 in HEK293^WT^ cells. Supporting the involvement of CUL family proteins, MLN4924 treatment abolished the ubiquitination of RbFox2 in HEK293^WT^ cells. To further elucidate the specific role of CUL3 in RhoBTB1-mediated ubiquitination of RbFox2, we ectopically coexpressed RbFox2 and ubiquitin, either with RhoBTB1 or with an empty vector in HEK293^CUL3KO^ cells. CUL3 deficiency reduced the RhoBTB1-dependent polyubiquitination of RbFox2 (Figure 6B). However, the polyubiquitination was not completely abolished in HEK293^CUL3KO^ cells, suggesting the potential role of other ubiquitinating proteins. Given this, we concluded that RhoBTB1 mediates the polyubiquitination of RbFox2 followed by its CUL3-dependent proteasomal degradation. Taken together, these results suggest that RbFox2 expression is regulated by RhoBTB1 and CUL3.

### RbFox2 modulates the cytoskeleton.

**Figure 7:**
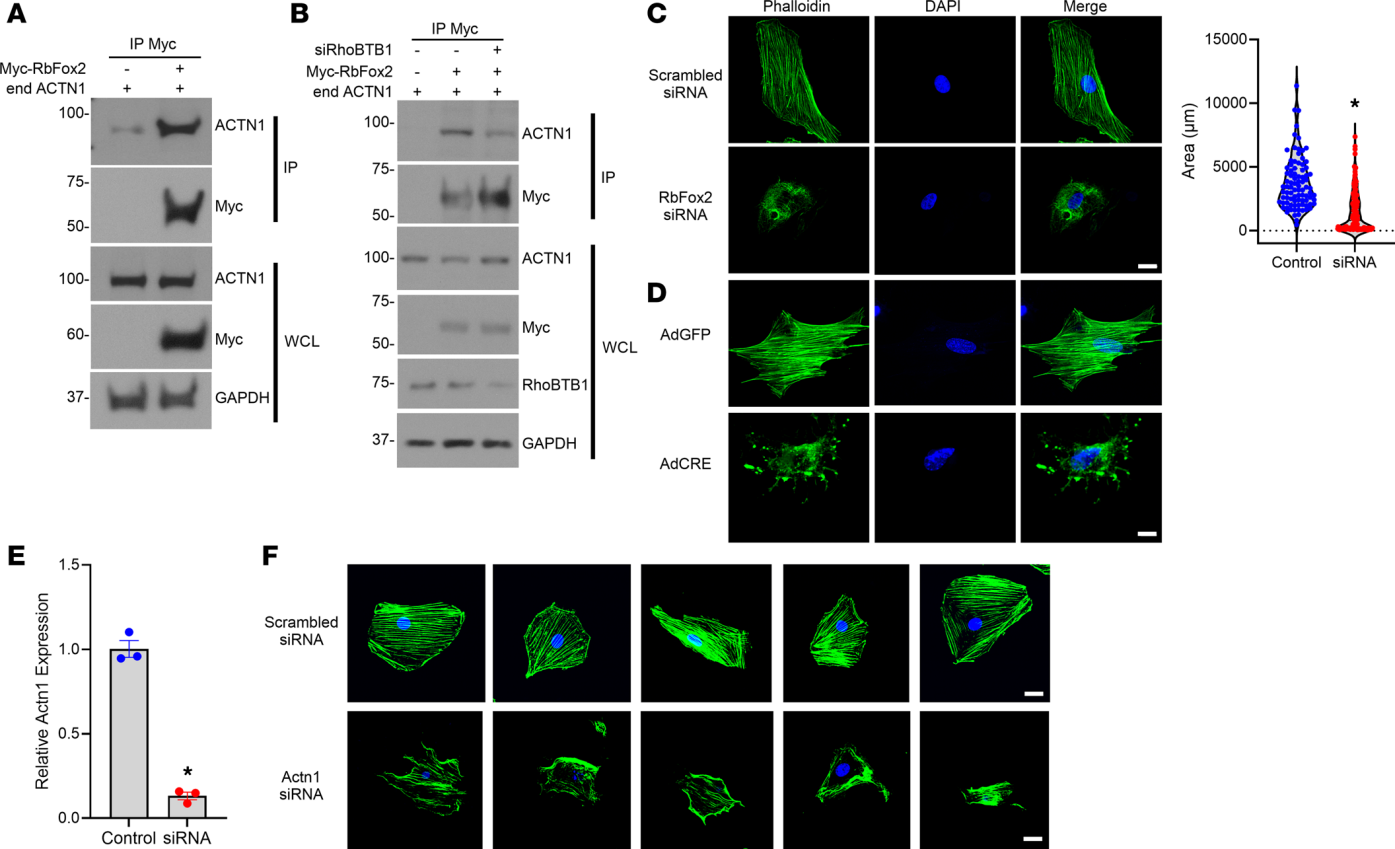
Interaction of RbFox2 with Actn1 and its role in cytoskeletal modulation. (**A**) Co-IP assay in A7R5 SMCs. Myc-tagged RbFox2 was transfected for 24 hours, followed by immunoprecipitation using anti-Myc antibodies and Western blotting with the indicated antibodies. The experiment was performed once but replicated in **B**. (**B**) Co-IP in A7R5 SMCs after RhoBTB1 knockdown. A7R5 cells were transfected with scrambled or RhoBTB1-targeting siRNA for 48 hours, then cotransfected with Myc-RbFox2 for the next 24 hours (total siRNA exposure: 72 hours). Co-IP was performed with anti-Myc beads and analyzed by Western blot using indicated antisera. IP, immunoprecipitation; WCL, whole-cell lysates. Molecular weight markers were translated from the original blots. Data represent 2 independent replicates. (**C**) Phalloidin staining (green) in A7R5 cells following *RbFox2* siRNA transfection (72 hours) compared with scrambled control. Images represent at least 7 different fields from duplicate independent experiments performed by 2 different researchers. DAPI as counterstain in confocal images shows nuclei. Violin plot quantifies the cell area differences. (**D**) Phalloidin staining in primary SMCs from *RbFox2^fl/fl^* mice infected with either AdGFP (control) or AdCRE (RbFox2 deletion). DAPI as counterstain in confocal images shows nuclei. Multiple fields and cells were examined from a single experiment. (**E**) Expression of *Actn1* in A7R5 cells in response to *Actn1* siRNA. *P < 0.001 by unpaired 2-tailed *t* test; *N* = 3. (**F**) Phalloidin staining (green) in A7R5 cells following *Actn1* siRNA transfection (72 hours) compared with scrambled control. Images represent fields from duplicate independent experiments. Scale bars: 10 μm (**C**, **D**, and **F**).

Our pathway enrichment analysis data suggested that the identified proteins were involved in altering the components of cellular cytoskeleton. We therefore performed co-IP to assess whether there was a direct interaction between RbFox2 and α-smooth muscle actin (αSMA). A7R5 cells were transfected with Myc epitope–tagged RbFox2, and immunocomplexes were probed for αSMA. However, we did not detect any direct interaction between RbFox2 and αSMA (data not shown). Consequently, we hypothesized that RbFox2 modulates the SMC cytoskeleton through actin-associated proteins. This hypothesis was of particular interest because we identified α-actinin-1 (Actn1) in the A7R5 proteome that bound to RhoBTB1, and because Actn1 is a key protein that regulates the organization of the actin cytoskeleton. Therefore, we examined the physical interaction between Actn1 and RbFox2. Immunoblotting demonstrated that RbFox2 directly interacted with Actn1 (Figure 7A). Interestingly, in an experiment replicated twice, the interaction between RbFox2 and Actn1 appeared to require RhoBTB1, since the level of pull-down of Actn1 was decreased in response to a siRNA targeting *RhoBTB1* (Figure 7B).

Given the interaction between RbFox2 and Actn1, we next sought to determine whether RbFox2 expression affects actin polymerization in SMCs. First, we transfected A7R5 SMCs with a siRNA targeting *RbFox2* and performed phalloidin staining. Fluorescence imaging revealed a greater extent of disorganized actin filaments and decreased cellular area in RbFox2-depleted SMCs in comparison with A7R5 cells with scrambled control siRNA (Figure 7C; additional examples in Supplemental Figure 6). Next, SMCs were isolated from mice carrying a conditional allele of *RbFox2^fl/fl^*. Deletion of *RbFox2* was induced by infection with an adenovirus expressing Cre recombinase (AdCre) with AdGFP serving as a control. AdCre-infected primary SMCs from *RbFox2^fl/fl^* mice exhibited a greater extent of actin filament disorganization (Figure 7D; additional examples in Supplemental Figure 7). We next tested the significance of Actn1 as a RhoBTB1 target by examining phalloidin staining in A7R5 cells in response to siRNA targeting *Actn1*, which resulted in an 87% decrease in *Actn1* expression (Figure 7E). Inhibition of *Actn1* expression resulted in disorganized actin filaments (Figure 7F; additional examples in Supplemental Figure 8).

### RbFox2 regulates arterial stiffness.

**Figure 8:**
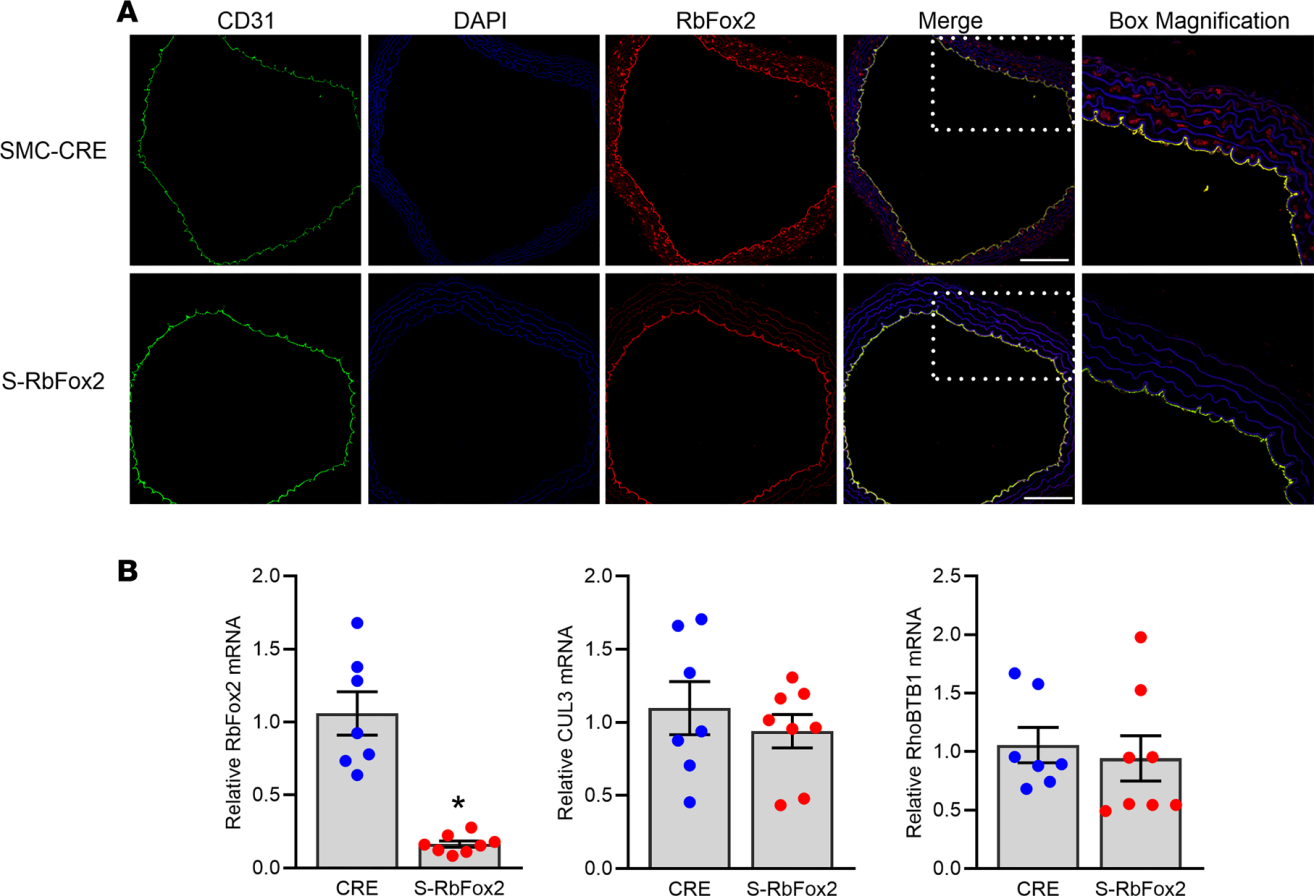
Vascular RbFox2 Expression. (**A**) Immunofluorescence images detecting RbFox2 (red) in SMC-CRE and S-RbFox2KO mice after ANG and Tx treatment. CD31 (green) indicates endothelial staining, while DAPI (blue) shows nuclei. The outlined areas in the merged panels are magnified in the far right column. Scale bars: 50 μm. (**B**) Summary graphs demonstrate the relative transcriptional level of *RbFox2*, *CUL3*, and *RhoBTB1* in aorta from ANG- and Tx-treated SMC-CRE and S-RbFox2KO mice as detected by real-time RT-PCR. *Gapdh* mRNA was used as internal control. Data represent mean ± SEM; calculated by the 2^–ΔΔCt^ method. *P < 0.05 by 2-tailed *t* test; *N* = 7 for SMC-CRE (CRE); *N* = 8 for S-RbFox2KO (S-RbFox2).

To assess the impact of SMC-specific deletion of RbFox2, we used double-transgenic mice carrying a conditional *RbFox2^fl/fl^* allele bred with an SMC-specific tamoxifen-inducible (Tx-inducible) Cre recombinase (termed S-RbFox2KO). SMC-CRE mice expressing Tx-inducible Cre recombinase were controls, and all mice were treated with Tx. Robust RbFox2 expression was observed in the medial layer of the aorta from control SMC-CRE mice (Figure 8A). In contrast, S-RbFox2KO mice exhibited markedly reduced RbFox2 expression in the medial layer. Expression of CD31, a marker of endothelial cells, was preserved in both groups. Reverse transcriptase PCR (RT-PCR) analysis revealed that *RbFox2* mRNA levels were decreased by about 80% (*P* < 0.05) in aorta from S-RbFox2KO mice, confirming efficient transcriptional silencing of *RbFox2* transgene (Figure 8B). In contrast, expression of *CUL3* and *RhoBTB1* mRNAs remained unaltered between the groups.

**Figure 9:**
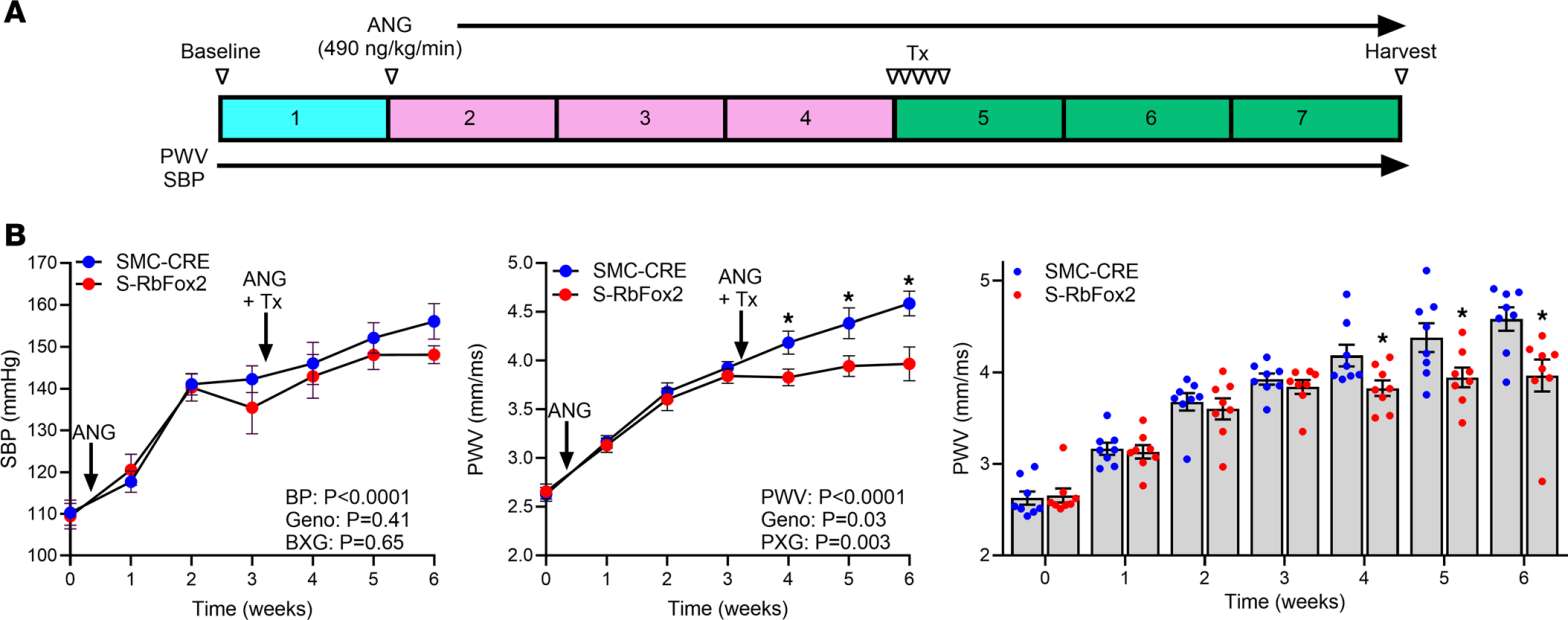
SBP and arterial stiffness in S-RbFox2KO mice. (**A**) Schematic of the experiment showing the time course for measurements. Baseline SBP and PWV were recorded in SMC-CRE and S-RbFox2KO mice for 1 week before ANG administration by osmotic minipump (week 1) and Tx injections. All animals received ANG for 3 weeks (weeks 2–4) followed by Tx injections for 5 consecutive days. SBP was recorded daily by tail-cuff plethysmography until the end of the protocol. PWV was measured before pump implantation, and once every subsequent week after pump implantation and Tx injections. Tissues were harvested at the end of the protocol. (**B**) Summary data for SBP and PWV in SMC-CRE (*n* = 8) and S-RbFox2KO mice (*n* = 8) at indicated time points. Arrows show the start of ANG and Tx. The dot plot shows individual PWV readings from the middle panel at each time point. BXG, BP X Genotype; PVG, PWV X Genotype. All data are represented as mean ± SEM. *P < 0.05 vs. SMC-CRE by 2-way ANOVA with Tukey’s multiple comparisons.

We next assessed whether RbFox2, like RhoBTB1, might also regulate arterial stiffness in vivo (14). We designed a reversal protocol where mice expressing SMC-CRE and S-RbFox2KO were treated with 490 ng/kg/min ANG via osmotic minipumps for 6 weeks (Figure 9A). SBP and pulse wave velocity (PWV) were measured weekly during baseline and throughout ANG administration. SBP was measured by tail-cuff plethysmography to ensure there was no interference with the measurement of arterial stiffness. After HTN was established, Tx was administered to induce deletion of RbFox2 in vascular SMCs. Both groups had equivalent SBP (109.48 ± 3.06 vs. 110.27 ± 3.01 mmHg, S-RbFox2KO vs. SMC-CRE) and PWV (2.66 ± 0.08 vs. 2.63 ± 0.07 mm/ms, S-RbFox2KO vs. SMC-CRE) at baseline (Figure 9B). Also, 3 weeks of ANG infusion elevated the SBP (135.44 ± 6.26 vs. 142.29 ± 3.22 mmHg, S-RbFox2KO vs. SMC-CRE) and PWV (3.84 ± 0.08 vs. 3.93 ± 0.06 mm/ms, S-RbFox2KO vs. SMC-CRE) in both groups. Despite the upward trend in SBP in both groups of mice, Tx-treated S-RbFox2KO mice exhibited a modest but statistically insignificant decline in SBP as compared with SMC-CRE mice (148.15 ± 2.14 vs. 156.09 ± 4.25 mmHg, S-RbFox2KO vs. SMC-CRE). In contrast, whereas PWV continued to increase in SMC-CRE mice (4.52 ± 0.17 mm/ms) 3 weeks after Tx (6 weeks after ANG), PWV remained stable in S-RbFox2KO mice (4.0 ± 0.19 mm/ms) after Tx. Examination of individual PWV data also supported the robustness of the declining arterial stiffness in response to RbFox2 deletion in SMCs (Figure 9B).

**Figure 10:**
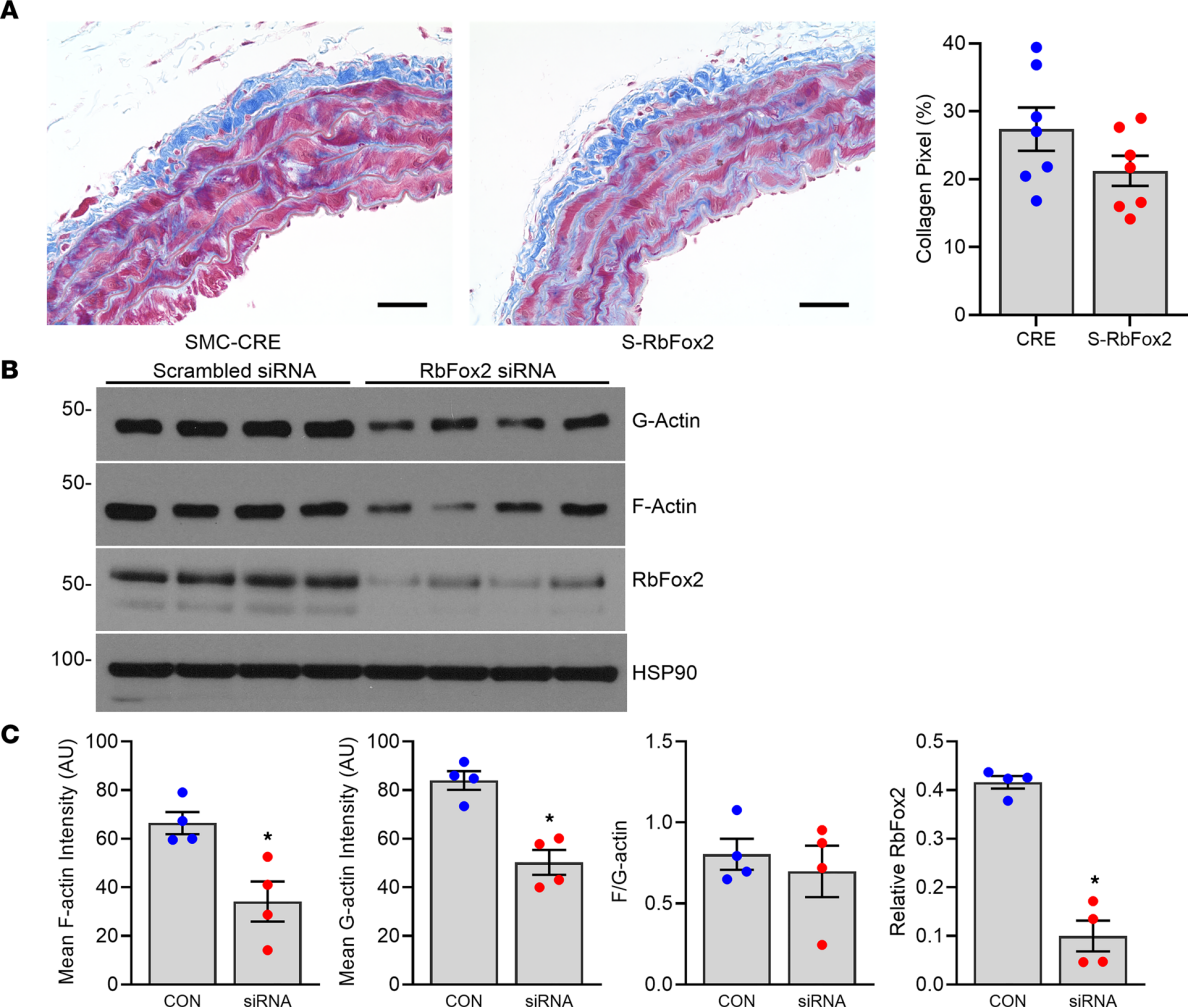
Role of RbFox2 in cytoskeletal modulation. (**A**) Representative histopathological images of aorta cross sections from indicated animals showing the deposition of collagen 3 weeks after ANG treatment followed by Tx treatment. Scale bars: 50 μm. Bar graph compares the collagen levels in aorta from indicated animals. *N* = 7 for SMC-CRE; *N* = 7 for S-RbFox2KO. Not significant by 2-tailed *t* test. (**B**) Western blot analysis of G-actin and F-actin in A7R5 cells transfected with *RbFox2*-targeting siRNA compared with cells transfected with scrambled control siRNA. Immunoblots were probed with indicated antisera. Molecular weight markers are translated from original blots. Represents a single experiment from 4 independent samples each. (**C**) Data summarizing the levels of F-actin and G-actin, F/G-actin ratio, and relative expression of RbFox2 in A7R5 cells before and after siRNA-mediated *RbFox2* knockdown. These data are representative of 4 separate siRNA transfections. All data are represented as mean ± SEM. *P < 0.05 by 2-tailed *t* test; *N* = 4 for both groups.

We next measured the extent of collagen deposition using Masson’s trichrome staining of aortic sections. Although there was a trend toward decreased collagen in aorta from S-RbFox2KO, this difference was not statistically significant (Figure 10A; additional examples in Supplemental Figure 9). Altogether, this suggests that RbFox2 may contribute to arterial stiffness, at least in part, through mechanisms beyond collagen-dependent vascular remodeling.

We next hypothesized that RbFox2 might regulate the polymerization of actin filaments. To investigate this, we returned to the A7R5 cell model and performed ultracentrifugation-based enrichment of polymerized actin (F-actin) and its globular isoform (G-actin) from whole-cell lysates of A7R5 cells transfected with siRNA targeting *RbFox2*. Depletion of RbFox2 led to a reduction in the total actin content as evident by the decreased levels of both F- and G-actin isoforms (Figure 10, B and C). Because of this, the ratio of F- to G-actin was unchanged in comparison with si*RbFox2* versus siRNA control. We conclude that RbFox2 regulates cytoskeletal integrity in SMCs likely through the modulation of the actin cytoskeleton.

### RbFox2 is a target of RhoBTB1 and CUL3 in human aortic SMCs.

**Figure 11:**
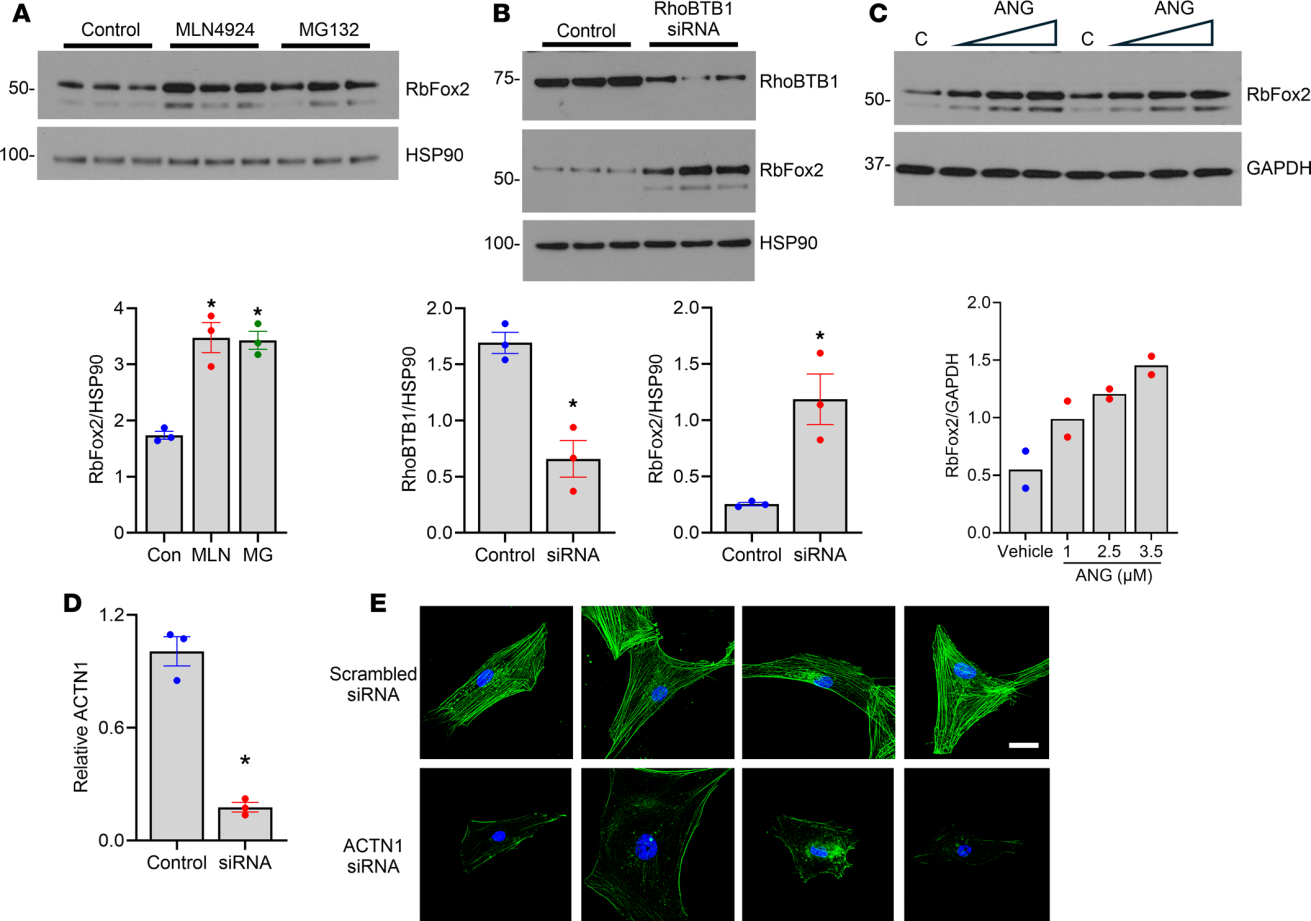
Role of RbFox2 and Actn1 in primary human aortic SMCs. (**A**) Immunoblot demonstrating the regulation of RbFox2 by CUL3-dependent ubiquitin-proteasome pathway in human aortic SMCs (HASMCs) treated with MLN4924 (1 µM) or MG132 (10 µM) for 24 hours. Hsp90 was used as a loading control. The experiment was performed with 3 independent samples and is quantified below. Data represent mean ± SEM; *P < 0.05 by 2-tailed *t* test; *N* = 3 each. (**B**) Relative levels of RbFox2 and RhoBTB1 in HASMCs transfected with scrambled control and siRNA targeting *RhoBTB1* for 72 hours. Hsp90 was used as an internal control for protein loading. Data are quantified below and represent mean ± SEM; *P < 0.05 by 2-tailed *t* test; *N* = 3. (**C**) Immunoblot measuring the expression of RbFox2 in response to ramped increases in ANG treatment (1.0, 2.5, 3.5 μM) of HASMCs. Data are quantified and represent mean of *N* = 2 samples. Immunoblots were probed with indicated antisera. Molecular weight markers are translated from original blots. (**D**) Real-time RT-PCR of *ACTN1* expression in response to scrambled siRNA (Control) and siRNA targeting *ACTN1* in HASMCs. Data represent mean ± SEM; *P < 0.05 by 2-tailed *t* test; *N* = 3 each. (**E**) Phalloidin staining (green) in HASMCs following *ACTN1* siRNA transfection (72 hours) compared with scrambled control. Images represent fields from duplicate independent experiments. Scale bar: 10 μm.

We next examined the significance of these findings in primary human aortic SMCs (HASMCs). First, RbFox2 expression increased in response to both MLN4924 and MG132, suggesting that it is a CUL3 target in HASMCs (Figure 11A). Second, expression of RbFox2 increased in response to siRNA targeting *RhoBTB1*, suggesting that it is a RhoBTB1 target (Figure 11B). Third, RbFox2 expression increased in response to ANG treatment of HASMCs (Figure 11C). Finally, siRNA targeting *Actn1* in HASMCs resulted in a marked downregulation of *Actn1* mRNA (Figure 11D), and a disruption of the actin cytoskeleton (Figure 11E; additional examples in Supplemental Figure 10). Thus, the findings identified using the A7R5 cell line have been validated in human vascular SMCs.

### RbFox2 regulates mRNA splicing.

**Figure 12:**
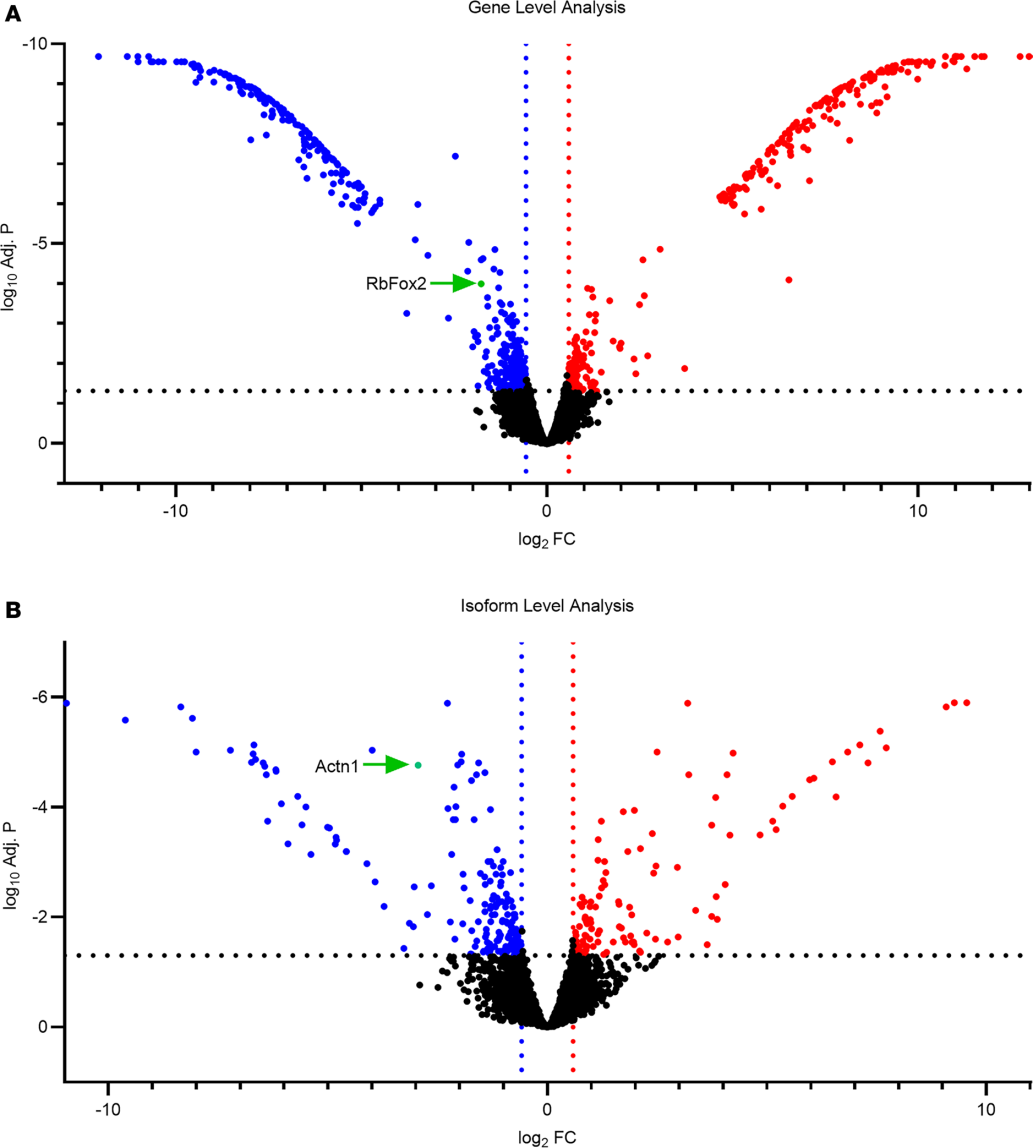
Role of RbFox2 in mRNA splicing. (**A**) Volcano plot representing gene-level abundance of mRNAs from A7R5 cells in the presence or absence of *RbFox2* siRNA. (**B**) Volcano plot representing isoform-level abundance of mRNAs from A7R5 cells in the presence or absence of *RbFox2* siRNA. There were 2 control siRNAs and 2 siRNAs targeting *RbFox2*.

RbFox2 is recognized as a factor affecting mRNA splicing (15, 25). To assess whether loss of RbFox2 caused aberrations in mRNA splicing, we performed isoform sequencing–based (Iso-Seq–based) RNA sequencing (RNA-seq) of A7R5 cells with and without a siRNA targeting *RbFox2*. Iso-Seq enables sequencing of full-length transcript isoforms from 5′-UTR to 3′ poly(A) tail. We validated the effectiveness of the siRNA targeting *RbFox2* in the previous experiment (Figure 10C). RNA-seq analysis confirmed downregulation of *RbFox2* by about 70% by the siRNA (Supplemental Dataset 2). Gene-level analysis revealed that inhibition of *RbFox2* caused the marked differential expression of over 650 mRNAs, suggesting a key role for RbFox2 in transcriptional regulation (Figure 12A). Mapping individual mRNA isoforms to the rat genome revealed the presence of unique isoforms of mRNA encoding nearly 300 genes, one of which was *Actn1* (Figure 12B). We focused on *Actn1* because it was identified as a RhoBTB1 binding protein in the targeted proteomic screen and was found to bind to RbFox2 in a RhoBTB1-dependent manner (Figure 7B). The presence or absence of RbFox2 resulted in several differential *Actn1* isoforms, particularly at the 3′ end of the mRNA, including isoforms that excluded exons and mis-spliced introns leading to intron inclusion of the mRNA (Supplemental Figure 11). We tested whether isoforms that exhibited intron inclusion at the 3′ end of the gene (Supplemental Figure 11, red arrows) were more abundant in RbFox2-intact cells (Supplemental Figure 12). A comparison of the RT-PCR signal of PCR products targeting the fully spliced and intron inclusion isoforms revealed that they were both more abundant in RbFox2-intact cells versus RbFox2-deficient cells. All RT-PCR products were confirmed by DNA sequencing. These data suggest that RbFox2 affects the abundance and splicing of key mRNAs that regulate the cytoskeleton.

## Discussion

Proximity ligation proteomics using APEX2-tagged domains of RhoBTB1 were employed to identify novel interacting partners and potential modulators of arterial stiffness. We first provided the foundational evidence that the RhoBTB1-APEX2 fusion constructs were expressed in A7R5 SMCs, were capable of binding with their previously known targets, and could efficiently biotinylate the SMC proteome (13). Gene set enrichment analysis revealed that many of the identified proteins were engaged in myosin and actin binding, cytoskeletal structure, contractile function, or calcium regulation. This suggests that the RhoBTB1/CUL3 pathway in SMCs may contribute to the regulation of vasomotor tone. These observations align with our previous studies showing that RhoBTB1 regulates NO responsiveness through PDE5 and arterial stiffness through undefined RhoBTB1 binding proteins (7, 12, 14). Thus, RhoBTB1 may have multiple functions in vascular SMCs designed to regulate vasomotor tone through mechanisms controlling both the state of NO responsiveness (e.g., vasodilation/contraction) and arterial stiffness through the cytoskeleton.

Our approach to increase the specificity and selectivity of the proteomic screen relied on our previous study in which we identified the C-terminal half of RhoBTB1 (also known as the B1B2C domains) as the minimum region critical for its adaptor function that delivers substrates for ubiquitination to the CUL3 complex (13). RhoBTB1 acts as a substrate recognition protein for PDE5, a negative regulator of cGMP-dependent NO signaling in vascular SMCs (12). The comparison between binding proteins that are associated with B1B2C, but not B1B2 (lacking the C-terminal domain), presumably allowed us to remove a large background of nonspecific interactions in the proximity labeling. Among the identified proteins, we selected 6 top-ranked proteins to validate the proteomic data using co-IP. Half of these proteins (Cald1, Pls3, and StomL2) were validated to bind to RhoBTB1. The other half — filamin A (Flna), tropomyosin 2 (Tpm2), and RhoQ — were intriguing targets because they are associated with the cytoskeleton or regulators of vasomotor function but did not bind to either B1B2C or full-length RhoBTB1. It remains possible that these proteins associate with other proteins that directly bind to RhoBTB1 or are located in proximity to RhoBTB1.

Ultimately, we focused on RbFox2 because of (a) its exclusive presence in several B1B2C replicates and complete absence in the B1B2 concurrent screen, (b) previous studies pointing to its regulation through the proteasomal pathway, and, most importantly, (c) the immediate availability of a conditional knockout model for *RbFox2^fl/fl^* (15, 26). In the first set of experiments, we evaluated the hypothesis that RbFox2 is regulated by the RhoBTB1/CUL3 pathway. Supporting this, we found that RbFox2 binds directly to RhoBTB1, and its expression is upregulated by (a) pharmacological inhibition of the cellular proteasomal machinery, (b) CUL3 deficiency, both in cells and in aorta, and (c) RhoBTB1 ablation. Expression of RbFox2 was also increased by ANG in cells and in vivo, presumably through a mechanism involving the downregulation of RhoBTB1 by ANG. The observation that ANG increased RbFox2 in both cells and aorta from ANG-treated mice likely rules out the possible contribution of hemodynamic interference from the HTN caused by ANG. Importantly, the regulation of RbFox2 expression by RhoBTB1/CUL3 was replicated in primary HASMCs.

We also ruled out a contribution to the previously identified RhoBTB1 target PDE5 by co-IP (12). PDE5 could be pulled down in HEK293 cells coexpressing RbFox2, PDE5, and RhoBTB1 (Supplemental Figure 13). However, PDE5’s contribution to the complex was eliminated by siRNA targeting *RhoBTB1*, suggesting that PDE5 interacts with RhoBTB1 and not directly with RbFox2. Further evidence that RbFox2 is a CUL3 target was gained by measurement of the ubiquitination of RbFox2. Higher ubiquitination of RbFox2 in HEK293^WT^ cells was observed in the presence of RhoBTB1, which was impaired in HEK293^CUL3KO^ cells. Interestingly, there was some residual ubiquitination of RbFox2 occurring even in HEK293^CUL3KO^ cells, which could be due to incomplete deletion of CUL3 (thus residual activity) or the possibility that RbFox2 ubiquitination is not completely confined to CUL3. If the latter is true, this protein would likely be another member of the Cullin family, because treatment of SMCs with MLN4924 (a pan-Cullin inhibitor) appeared to completely abolish ubiquitination of RbFox2.

Based on gene set enrichment analysis, we hypothesized that RbFox2 might regulate the cytoskeleton. We evaluated this by two strategies. First, siRNA targeting of *RbFox2* in A7R5 cells led to profound rearrangement of the cellular cytoskeleton as detected by phalloidin staining. Similarly, aortic SMCs from *RbFox2^fl/fl^* mice induced to RbFox2 deficiency by AdCre infection caused a rearrangement of the cytoskeleton. Second, this prompted us to assess the importance of RbFox2 in a model of ANG-induced HTN and arterial stiffness in vivo. *RbFox2^fl/fl^* mice were bred with an inducible SMC-specific Cre-driver line that resulted in effective deletion of RbFox2 in medial SMCs of the aorta with preservation of RbFox2 expression in the endothelium. As expected, ANG caused a progressive rise in SBP and PWV in both groups of mice before the deletion of RbFox2. PWV is a sonographic measure of the speed with which a pressure wave travels through the arterial tree and is a well-accepted index of arterial stiffness. Whereas PWV continued to increase in control mice after Tx, the PWV in S-RbFox2KO mice remained steady. Halting of the rise of PWV in S-RbFox2KO mice occurred even though BP remained similar in both groups. This suggests that hemodynamic factors are not responsible for the dampened rise in arterial stiffness. Interestingly, the effects of RbFox2 ablation were less pronounced than in our previous study, in which we overexpressed RhoBTB1 selectively in vascular SMCs. In those mice, PWV markedly decreased, returning nearly to baseline after 3 weeks of RhoBTB1 expression (14). Recall that increased expression of RhoBTB1 translates to decreased RbFox2, because RhoBTB1 promotes RbFox2 ubiquitination and degradation. These data would suggest that multiple RhoBTB1 target proteins are involved in the regulation of arterial stiffness, and that RbFox2 is one component of that response. This is consistent with the proteomic data showing that numerous cytoskeletal proteins are RhoBTB1 targets. Future studies will examine the contribution of other RhoBTB1 targets.

It is notable that SMC-specific deletion of RbFox2 improves (or at least halts the further increase in) arterial stiffness even with preservation of the pressor effect of ANG. This is also consistent with our previous studies in which we restored expression of RhoBTB1 in the ANG model (14). There is a known relationship between arterial BP and arterial stiffness, and increases in BP are causative for increased arterial stiffness (3, 27). Our data showing a reversal of arterial stiffness in response to RhoBTB1, which would decrease RbFox2 and other RhoBTB1-CUL3 targets, and a halting of further increases in arterial stiffness by RbFox2 deletion suggest that improvements in arterial stiffness can be induced even in the presence of continued HTN. This is not unique to RhoBTB1, as a separation between arterial stiffness and pulse pressure has been previously observed in mice deficient in Bcl11b (28).

Finally, we examined the mechanism by which RbFox2 regulates arterial stiffness. First, we ruled out major changes to collagen deposition in the aorta, as only modest (not statistically significant) changes were noted. The results of the phalloidin staining experiments and our surprising finding that RbFox2 may bind directly to α-actinin-1 (Actn1) in a RhoBTB1-dependent manner suggested an effect directly on the cytoskeleton. Indeed, siRNA targeting *Actn1* in either A7R5 cells or primary HASMCs resulted in a disorganization of the actin cytoskeleton. To validate this, we returned to A7R5 cells and observed a decrease in the level of both F (filamentous and polymerized) and G (soluble and monomeric) actin isoforms in RbFox2-deficient cells compared with cells receiving nonspecific siRNA. The importance of the actin cytoskeleton for the regulation of arterial stiffness has been reviewed (29). Indeed, the ratio of F- to G-actin has been reported to increase in response to contractile stimulation (30, 31). This result differs from that of our previous study in which we induced overexpressed RhoBTB1 in vascular SMCs in the ANG model, which caused an increase in the F- to G-actin ratio (14), whereas here a siRNA targeting *RbFox2* decreased both forms of actin. The physiological relevance of this difference in F/G-actin ratio remains unclear, but it may reflect the effects of RbFox2 on mRNA splicing, in particular *Actn1*. 

Our data are not the first to report protein-protein interactions between RbFox2 and other proteins (25). RbFox2 binds to RNA splicing factors including heterogeneous nuclear ribonucleoprotein C (Hnrnpc), heterogeneous nuclear ribonucleoprotein M (Hnrnpm), and serine- and arginine-rich splicing factor 1 (Srsf1), but here we provide evidence for a direct association with a non-splicing factor. That RbFox2 is a recognized RNA splicing factor deserves some comment. Indeed, it is possible that RbFox2 may regulate alternative splicing events of RNAs of genes encoding the components of the cytoskeleton or other aspects of vascular function. Iso-Seq RNA-seq analysis revealed that siRNA-mediated ablation of *RbFox2* in A7R5 cells resulted in differential expression and splicing of many mRNAs, including *Actn1*. It is notable that RbFox2 was reported to regulate the splicing of the L-type calcium (Ca^2+^) Ca_V_1.2 channels, which are essential for cardiomyocyte excitation and contraction (32). Abnormal Ca_V_1.2 channels are seen in diabetic cardiomyopathy, diastolic dysfunction, and cardiac hypertrophy in rats. Disruption of the actin cytoskeleton alters the pattern of Ca_V_1.2 channels from recycling toward lysosomal degradation, presumably leading to disturbed Ca^2+^ homeostasis in cardiomyocytes (33). Paradoxically, elevated expression of RbFox2 has also been detected in spontaneously hypertensive rats, in type 2 diabetic human patients, and in impaired Ca^2+^ signaling (34, 35). Furthermore, dominant-negative RbFox2 isoforms have been detected at the early stages of cardiomyopathy, and aberrantly expressed RbFox2 isoforms are positively linked to myotonic dystrophy 1–mediated (DM1-mediated) cardiac pathogenesis (34, 36).

In conclusion, our data collectively support the concept that RbFox2 is a RhoBTB1 binding protein that is itself regulated by the RhoBTB1/CUL3 pathway and that manipulating this pathway pharmacologically may provide therapeutic targets for the treatment of cardiovascular disease risk factors such as arterial stiffness.

## Methods

### Sex as a biological variable.

Male mice were studied, because the *Myh11*-Cre^ERT2^ transgene is inserted on the Y chromosome (22).

### Cell culture.

HEK293^WT^, HEK293^CUL3KO^, and A7R5 cells were cultured in Dulbecco’s modified Eagle medium (DMEM; 11885084, Gibco, Thermo Fisher Scientific) supplemented with 5% fetal bovine serum (FBS; 10082147, Gibco, Thermo Fisher Scientific) and 1% penicillin-streptomycin (15140122, Gibco, Thermo Fisher Scientific) maintained at 37°C with 5% CO_2_ (13). Primary HASMCs (PCS-100-012, ATCC) were cultured in vascular cell basal medium (PCS-100-030, ATCC) supplemented with vascular smooth muscle growth kit (PCS-100-042, ATCC) comprising 5 ng/mL fetal growth factor, 4 μg/mL insulin, 5 ng/mL epidermal growth factor, 10 nM l-glutamine, 50 ng/mL ascorbic acid, 5% FBS, and 1% penicillin-streptomycin. These cells were derived from a 57-year-old African American man.

### Mouse aortic SMCs.

Mice were injected with heparin (300 μL) for 3 minutes and were euthanized. The aorta was dissected and incubated with digestion buffer A containing collagenase A (1 mg/mL; 10103586001, Sigma-Aldrich), collagenase B (1 mg/mL; 11088815001, Sigma-Aldrich), and DNase I (0.1 mg/mL; LS002139, Worthington Biochemical Corp.) reconstituted in serum-free DMEM, in a CO_2_ incubator for 15 minutes. The adventitia was removed with a pair of sterile forceps, and the aorta was washed in cold Dulbecco’s PBS (DPBS; J67802-K2, Thermo Fisher Scientific; three 15-second washes) and then incubated in serum-free DMEM for 30 seconds, transferred in digestion buffer B (digestion buffer A supplemented with elastase; 0.125 mg/mL; E0127, Sigma-Aldrich), minced, and incubated in a CO_2_ incubator for 40 minutes. Cells were dissociated by vigorous pipetting for 10 minutes at room temperature followed by centrifugation at 125*g* for 10 minutes (4°C). The cell pellet was resuspended in DMEM supplemented with 5% FBS and 1% penicillin-streptomycin. The cell suspension was filtered through a 70 μm sterile cell strainer (352350, Corning) and plated in 35 mm culture dishes (130180, Thermo Fisher Scientific). Cells were left undisturbed for 3 days, after which the culture medium was replaced. Cells were subsequently maintained in a humidified atmosphere (37°C, 5% CO_2_) and treated with AdCre and AdGFP in separate culture dishes (72 hours).

### Western blotting.

Cells were lysed using RIPA buffer (50 mM Tris-HCl [pH 7.5], 150 mM NaCl, 1 mM Na_2_EDTA, 1 mM EGTA, 1% NP-40, 1% sodium deoxycholate, 2.5 mM sodium pyrophosphate, 0.1% SDS, 1 mM β-glycerophosphate, 1 mM Na_3_VO_4_) supplemented with protease and phosphatase inhibitor (78442, Thermo Fisher Scientific) and 1 mM phenylmethylsulfonyl fluoride (PMSF; 36978, Thermo Fisher Scientific). Cells were rotated in 1 mL centrifuge tubes (10 minutes, 4°C). Supernatants were collected by centrifugation of the homogenates at 13,000*g* (10 minutes, 4°C). Fifty micrograms of protein lysates were resolved using SDS–polyacrylamide gel electrophoresis (PAGE; 4% to 20%) and transferred to nitrocellulose (88018, Thermo Fisher Scientific). Membranes were blocked using 3% bovine serum albumin (BSA) in 1× Tris-buffered saline (TBS; 1706435, Bio-Rad Laboratories) containing 0.1% Tween 20 (0.1% TBST; P1379, Sigma-Aldrich) (1 hour, room temperature). Membranes were incubated with primary antibodies overnight (4°C). The following primary antibodies were used: rabbit anti-RbFox2 (12498-1-AP, Proteintech), rabbit anti-CUL3 (10450S, Cell Signaling Technology), mouse anti-HSP90 (60318-1-Ig, Proteintech), mouse anti-GAPDH (sc-47724, Santa Cruz Biotechnology), rabbit anti-RhoBTB1 (PA5-42077, Invitrogen, Thermo Fisher Scientific), and mouse anti–β-actin (58169S, Cell Signaling Technology). The next day, membranes were incubated with IRDye-conjugated secondary antibodies (926-32211 and 926-68070, LI-COR Biosciences) at 1:20,000 dilution in blocking buffer and horseradish peroxidase–linked (HRP-linked) secondary antibodies (7074S and 7076S, Cell Signaling Technology) at 1:3,000 dilution in 0.1% TBST (1 hour, room temperature).

### Co-IP.

A7R5 SMCs were treated with 1 μM MLN4924 and 10 μM MG132 after transfection (13). To determine the interaction between RhoBTB1, PDE5, and RbFox2, HEK293 cells were transfected with siRNA targeting endogenous RhoBTB1. After 48 hours of siRNA transfection, cells were transfected with plasmids encoding Myc-tagged RhoBTB1, His-PDE5, tag-free RhoBTB1, and Myc-RbFox2. Twenty-four hours after transfection, cells were washed with ice-cold DPBS and lysed on ice using IP lysis buffer (20 mM Tris-HCl [pH 7.5], 150 mM NaCl, 1 mM Na_2_EDTA, 1 mM EGTA, 1% Triton X-100, 2.5 mM sodium pyrophosphate, 1 mM β-glycerophosphate, 1 mM Na_3_VO_4_, 1 μg/mL leupeptin, and 1 mM PMSF reconstituted in isopropanol). Cells were collected and rotated in 1 mL centrifuge tubes (30 minutes, 4°C). Supernatants were collected by centrifugation at 13,000*g* (10 minutes, 4°C). Protein lysates (1,500 μg) were incubated with 20 μL of mouse anti-Myc magnetic bead conjugate (5698, Cell Signaling Technology) overnight at 4°C with rotation. Magnetic beads were separated using a magnetic rack and washed 3 times (2 minutes each) with IP washing buffer (20 mM Tris-HCl [pH 7.5], 150 mM NaCl, 0.5 mM EDTA) at 4°C with rotation. Immunocomplexes were dissociated by boiling in 4× LDS sample buffer (NP0007, Invitrogen, Thermo Fisher Scientific), resolved by SDS-PAGE, and transferred to nitrocellulose. Membranes were blocked in EasyBlocker (GTX425858, GeneTex Inc.) reconstituted in 0.1% TBST for 1 hour (room temperature) and incubated with appropriate primary antibodies. The following antibodies were used: rabbit anti-Myc (2278S, Cell Signaling Technology), rabbit anti-CALD1 (20887-1-AP, Proteintech), rabbit anti-STOML2 (10348-1-AP, Proteintech), rabbit anti-RbFox2 (12498-1-AP, Proteintech), mouse anti-RbFox2 (66976-1-Ig, Proteintech), rabbit anti-PLS3 (12917-1-AP, Proteintech), rabbit anti-ubiquitin (43124S, Cell Signaling Technology), rabbit anti-His (2365S, Cell Signaling Technology), rabbit anti-ACTN1 (3134S, Cell Signaling Technology), rabbit anti–α-SMA (14968S, Cell Signaling Technology), rabbit anti-FLNA (67133-1-Ig, Proteintech), rabbit anti-TPM2 (11038-1-AP, Proteintech), rabbit anti-PDE5 (2395S, Cell Signaling Technology), and rabbit anti-RhoQ (17805-1-AP, Proteintech). The next day, membranes were incubated with HRP-conjugated EasyBlot secondary antibodies (GTX221666-01 and GTX221667-01, GeneTex Inc.) at 1:2,000 dilution in 0.1% TBST (1 hour, room temperature).

### Ubiquitination assay.

Transfected cells were treated with 10 μM MG132 (M7449, Sigma-Aldrich) or 1 μM MLN4924 (951950-33-7, Calbiochem) immediately after transfection. Cell lysates were prepared in RIPA lysis buffer to maintain the reducing environment (12). Under these conditions RhoBTB1 is not pulled down. The immunoprecipitation was performed, and blots were developed as above.

### Transfection and RNA interference.

A7R5, HEK293, and HEK293^CUL3KO^ cells were transfected with Myc epitope–tagged and Myc-APEX2–tagged RhoBTB1 B1B2 and B1B2C domains using Lipofectamine LTX-PLUS reagent (A12621, Thermo Fisher Scientific) for 24 hours. Control cells were transfected with pcDNA3.1. SMARTPool siRNA targeting *RbFox2* (L-080094-02-0005, Horizon Discovery), *Actn1* (L-088980-02-0005, Horizon Discovery), and RhoBTB1 (L-094796-02-0005, Horizon Discovery) were transfected into A7R5 cells and HASMCs using DharmaFECT transfection reagent 2 (T-2002-02, Horizon Discovery) (72 hours). Control cells were transfected with negative control siRNAs (D-001810-10-05, Horizon Discovery).

### Phalloidin staining.

A7R5 cells and primary SMCs from *RbFox2^fl/fl^* mice were cultured in 35 mm dishes with a glass bottom (P35GC-0-14-C, MatTek Corp.). A7R5 cells were transfected with siRNA targeting *RbFox2* and *Actn1* or SMCs from *RbFox2^fl/fl^* mice were treated with AdCre (2 × 10^7^ PFU/dish; University of Iowa Vector Core, Iowa City, Iowa, USA) in 10 μg Polybrene (TR-1003, Sigma-Aldrich). HASMCs were transfected with siRNA targeting *Actn1* for 72 hours. Controls received scrambled control siRNA or AdGFP. Cells were washed 3 times with prewarmed PBS (20012050, Thermo Fisher Scientific) and fixed with 3.7% methanol-free formaldehyde in PBS for 15 minutes at room temperature. Cells were washed with PBS and permeabilized in 0.1% Triton X-100 (A16046-AE, Thermo Fisher Scientific) in PBS and were washed again with PBS with 1% BSA (45 minutes, room temperature). Cells were incubated in phalloidin (Alexa Fluor 488, A12379, Thermo Fisher Scientific; reconstituted as 400× stock in DMSO) diluted to 1× concentration in 1% BSA in PBS (1 hour, room temperature) in the dark. Cells were washed again with PBS and mounted in ProLong Glass Antifade hard-setting mountant containing NucBlue DNA stain (P36985, Thermo Fisher Scientific). Images were acquired with a confocal laser scanning microscope (LSM510, Zeiss) and analyzed using the AIM 4.2 software, or acquired with an LSM510 Nikon A1 microscope with ×60 objective and analyzed with ImageJ software (NIH).

### Real-time RT-PCR.

RNA was isolated from aorta, and RNA (250 ng) was reverse-transcribed using an iScript cDNA Synthesis Kit (1708891, Bio-Rad Laboratories) at 25°C for 5 minutes, 46°C for 20 minutes, and finally 95°C for 1 minutes. cDNA was diluted 1:4, and gene expression was measured using Fast TaqMan Master Mix (4385612, Applied Biosystems, Thermo Fisher Scientific). TaqMan assays were for *Rbfox2* (Mm01197021_m1), *Cul3* (Mm00516747_m1), *Rhobtb1* (Mm00143659_m1), *Actn1* (Rn00667357_m1), and *Gapdh* (Mm99999915_g1). The relative levels of mRNA expression were normalized to *Gapdh* and analyzed by 2^−ΔΔCt^ method (37).

### Biotinylation and proximity labeling by APEX2.

APEX2 proximity labeling was performed as described previously (13). A7R5 cells were treated with 1 μM MLN4924 and 10 μM MG132 after 16 hours and 20 hours of transfection, respectively, incubated in 500 μM biotin phenol reconstituted in complete medium at 37°C for 30 minutes, and exposed to flash exposure of 1 mM H_2_O_2_ (H1009, Sigma-Aldrich) for 1 minute. Biotinylation was quenched with 3 washes of cold DPBS containing 5 mM Trolox (238813, Sigma-Aldrich), 10 mM sodium ascorbate (PHR1279, Sigma-Aldrich), and sodium azide (190385000, Thermo Fisher Scientific). Cells were harvested and lysed in RIPA lysis buffer containing 1 mM PMSF, protease inhibitor cocktail (78430, Thermo Fisher Scientific), and the reaction quenchers and centrifuged at 15,000*g* (4°C, 10 minutes). Biotinylated proteins were enriched using streptavidin magnetic beads (88817, Thermo Fisher Scientific) by incubation of the biotinylated proteome with rotation at 4°C overnight. Beads were subsequently washed to remove nonspecific binders with RIPA buffer (2 washes) with the addition of 1 M KCl (P41025-500.0, Research Products International), 0.1 M Na_2_CO_3_ (L13098.36, Thermo Fisher Scientific), 2 M urea (15505035, Thermo Fisher Scientific), in 10 mM Tris (pH 8.0; T5941, Sigma-Aldrich) and then with RIPA buffer alone (2 washes). The biotinylated proteins were eluted by boiling of the beads in 4× protein loading buffer containing 20 mM DTT (D9779, Sigma-Aldrich) and 2 mM biotin (B4501, Sigma-Aldrich) for 5 minutes followed by SDS-PAGE and Western blotting as described above. Blocking of membranes was done in 3% BSA overnight on a rocker platform, and immunoblotting was done using streptavidin-HRP conjugate (1 hour, room temperature). Biotinylation was visualized by enhanced chemiluminescence reagents.

### Mass spectrometry.

Beads were placed on a magnetic rack to remove the suspension buffer and resuspended in 100 μL of 40% Invitrosol (MS1000, Invitrogen, Thermo Fisher Scientific) prepared in 100 mM ammonium bicarbonate (09830, Fluka) (13). Cysteines were reduced with 5 mM TCEP (646547, Sigma-Aldrich) (30 minutes, 37°C) followed by alkylation with 10 mM iodoacetamide (100351, MP Biomedicals) (30 minutes, 37°C). Proteins bound to the streptavidin beads were digested on beads overnight using 5 μg of trypsin (PR-V5113, Promega, Thermo Fisher Scientific) (37°C). Peptides were cleaned using the SP2 protocol (13). For retention time normalization, the Pierce Peptide Retention Time Calibration Mixture (88321, Thermo Fisher Scientific) was added at a final concentration of 4 nM during dilution of the samples to 20 ng/μL.

Samples were analyzed with a Thermo Fisher Scientific Orbitrap Fusion Lumos mass spectrometer using data-dependent acquisition with HCD MS2. We examined 12 total samples (3 technical replicates each of 2 biological samples) for B1B2C and B1B2. Supplemental Table 2 provides instrument settings. A pooled quality control sample, comprising equal portions of all experimental samples, was run at the beginning and the end and between each block of technical replicates in the acquisition sequence to monitor instrument performance and consistency. Raw MS data were processed using Proteome Discoverer v2.4 (Thermo Fisher Scientific) (Supplemental Table 3). Protein identification required at least 2 unique peptides per protein for inclusion in downstream analysis.

### Experimental animals.

Mice carrying a conditional *RbFox2* allele carrying *loxP* sites flanking exons 6–7 (*RbFox2^fl/fl^*, *B6.129S2-Rbfox2^tm1.1Dblk^/J*) were maintained on a C57BL/6 background (strain 014090, The Jackson Laboratory). They were bred with mice expressing a smooth muscle–specific Tx-inducible Cre recombinase (*Myh11*-Cre^ERT2^, herein referred to as SMC-CRE). The SMC-CRE^+^/*RbFox2^fl/fl^* mice (8–12 weeks of age) were treated with Tx (75 mg/kg, i.p. daily, 5 days, reconstituted in corn oil) to promote SMC-specific deletion (termed S-RbFox2KO). SMC-CRE mice expressing Tx-inducible Cre recombinase were used to control for any nonspecific effects. Only males were studied since this SMC-CRE is inserted into the Y chromosome.

### Immunostaining and Masson’s trichrome staining.

Mice were euthanized with CO_2_ and perfused with cold PBS. Aortae were isolated and fixed under physiological pressure using 4% paraformaldehyde (12 hours), paraffin-embedded, and sectioned at 4 μm. All immune stains were performed using a Leica Bond Rx immunostaining platform. Slides were dewaxed and antigen retrieved using reagent Epitope Retrieval solution H2 (AR9640, Leica) and incubated with primary antibodies (90 minutes, room temperature). The following antibodies were used: RbFox2 (12498-1-AP, Proteintech; 1:500) and CD31 (AF3628, Bio-techne; 1:100) diluted in antibody diluent (AR9352, Leica). After washing with Dewax solution (AR9222, Leica), secondary antibodies were applied and incubated for 45 minutes at room temperature followed by washing in wash buffer. The following secondary antibodies were used: donkey anti-rabbit–Cy3 (711.166.1520, Jackson ImmunoResearch; 1:500) and donkey anti-goat–Alexa Fluor 488 (A11055, Invitrogen, Thermo Fisher Scientific; 1:400). 4′-6-diamidino-2-phenylindole dihydrochloride (DAPI; D8417, Sigma-Aldrich; 1:5,000) was used as nuclear counterstain before mounting with ProLong Gold (P36930, Invitrogen, Thermo Fisher Scientific). Images were acquired using a Nikon A1R laser scanning confocal microscope equipped with a ×20 objective and NIS-Elements AR software (v5.42.02, Nikon Corp.).

For Masson’s trichrome, paraffin-embedded aorta sections were deparaffinized in xylene (3 times, 5 minutes each), rehydrated through graded ethanol (100% and 95%), and postfixed in Bouin’s fluid (1020B, NewComer) (60°C, 1 hour). After washing, slides were stained with Weigert’s Iron Hematoxylin (1409A, NewComer) (10 minutes) and stained in Biebrich Scarlet-Acid Fuchsin aqueous stain (10161C, NewComer) (4 minutes), rinsed, and placed in phosphotungstic-phosphomolybdic acid (1332C, NewComer) (12 minutes) followed by Aniline Blue (10072C, NewComer) (4 minutes). After washing in distilled water, slides were quickly dehydrated in graded ethanol (95% and 100%), cleared using xylene, and mounted with coverslips (6419, Tissue-Tek, Sakura). Images were captured with a Keyence fluorescence microscope equipped with a BZ-X800 Viewer and ×40 objective (BZ-X810, Keyence Corp.) using bright-field. All images were analyzed using QuPath software (https://qupath.github.io/).

### Blood pressure and PWV.

SBP was assessed using tail-cuff plethysmography (Visitech 2000 system) in order to avoid interference with simultaneous PWV measurements (38). Mice were trained for 3 weeks prior to 1 week of baseline measurements. BP was monitored 5 days per week, continuing throughout the ANG infusion. Mice were placed on a warmed platform (37°C), the initial 10 readings were discarded, and 30 measurements were recorded per session. A minimum of 15 valid readings (≥50% success rate) was required for data inclusion. The number of attempts, number of successful recordings, heart rate, and daily mean SBP were documented. Body weight was recorded once a week.

Arterial stiffness was assessed by PWV using the Doppler Flow Velocity System (Indus Instruments) (14). Mice were anesthetized with 2% isoflurane (5260-04-05, Abbott Laboratories) delivered in oxygen at 2 L/min and positioned supine on a heating pad. An electrocardiogram electrode embedded in the pad was used to monitor heart rate. Two 20-MHz ultrasound probes were placed over the descending and abdominal aorta to capture the arrival times of the pulse wave. Transit times were averaged across 5 cardiac cycles. PWV was calculated by dividing the distance between probe positions by the transit time.

### Actin polymerization.

Actin polymerization was assessed using a G-actin/F-actin in vivo assay kit (BK037, Cytoskeleton Inc.). A7R5 cells transfected with siRNA targeting *RbFox2* were harvested and lysed in buffer containing proprietary F-actin stabilizing reagents provided with the kit. Lysates were incubated at 37°C for 10 minutes, and 100 μL of lysates were centrifuged at 350*g* for 5 minutes at room temperature to pellet unbroken cells and debris. Supernatants were enriched by ultracentrifugation at 100,000*g* (1 hour). The supernatant containing G-actin was gently collected while the pellet containing F-actin was resuspended in 100 μL of F-actin depolymerization buffer and incubated on ice for 1 hour with vigorous vertexing at 15-minute intervals. Equal volumes of enriched F- and G-actin fractions were mixed with 4× SDS sample buffer and analyzed by Western blotting. Primary antibody was mouse anti-actin (AAN02, Cytoskeleton Inc.). Band intensities were quantified using ImageJ.

### Iso-Seq sequencing, processing, and analysis.

Total RNA was isolated from A7R5 cells transfected with *RbFox2* siRNA and matched scrambled control siRNA for long-read sequencing (39, 40). Library generation and sequencing were performed by Novogene. Total RNA was converted to first-strand cDNA (103-072-000, Kinnex full-length RNA Kit, Pacific Biosciences [PacBio]). First-strand products were PCR-amplified and barcoded using Iso-Seq bc01–bc12 primers supplied with an Iso-Seq Express 2.0 Kit (103-071-500, PacBio) at 98°C for 45 seconds, 98°C for 10 seconds, 60°C for 15 seconds, 72°C for 3 minutes, and 72°C for 5 minutes. The amplified cDNAs were cleaned using 0.9× SMRTbell Cleanup beads (102-158-300, PacBio) and pooled. Kinnex PCR was performed with Kinnex primers to generate DNA fragments containing orientation-specific Kinnex segmentation sequences using a Kinnex PCR 8-fold kit (103-071-600, PacBio). Kinnex PCR products were pooled and cleaned with 1.05× SMRTbell Cleanup beads. Next, cDNA segments were treated with Kinnex enzyme, ligase, and barcoded Kinnex terminal adaptors supplied with the Kinnex PCR kit and assembled into linear arrays. The cleanup step was performed once more, and the reaction mixture was subjected to nuclease treatment. Final cleanup of the reaction mixture was performed with cleanup beads. SMRTbell sequencing library was prepared using a Revio SPRQ polymerase kit following the manufacturer’s instructions (103-520-100, PacBio) and subsequently sequenced on a Revio PacBio sequencing platform.

We performed initial data processing using the Iso-Seq v4.3.0 pipeline (https://isoseq.how/getting-started.html). We determined whether each read was full-length based on the existence of 5′ and 3′ primers by using lima v2.13.0. Full-length reads were then refined by trimming of poly(A) tails and removal of artificial concatemers using the subprogram *refine* in the Iso-Seq package. The resulting full-length non-concatemer reads were clustered to the isoform level by running of the Iso-Seq *cluster2* subprogram. The clustered isoform-level reads were aligned to the rat genome (mRatBN7.2) using pbmm2 v1.17.0. The aligned reads were collapsed by running of the Iso-Seq *collapse* subprogram. Subsequently, transcriptome classification and filtering were performed using pigeon v1.4.0 (https://isoseq.how/classification/pigeon.html) and v112 of the *Rattus norvegicus* Ensembl annotation (Rattus_norvegicus.mRatBN7.2.112.chr.gtf).

For differential expression, we used lima v2.13.0 to remove 5′ and 3′ primers, and full-length reads were aligned to the rat genome (mRatBN7.2) using pbmm2 v1.17.0. IsoQuant v3.5.0 was used to quantify the aligned BAMs at the gene and transcript levels using v112 of the *R. norvegicus* Ensembl annotation (Rattus_norvegicus.mRatBN7.2.112.chr.gtf). Differential expression analysis between control and si*RbFox2* conditions was performed using the edgeR package (version 4.0.16) at both the gene level and the transcript level (41). Genes or transcripts with Benjamini-Hochberg–adjusted *P* values less than 0.05 and fold change greater than 1.5 were assigned as differentially expressed. A focus on smooth muscle–specific markers expressed in A7R5 cells is presented in Supplemental Table 4.

Isoforms overlapping the *Actn1* loci were extracted from the filtered transcriptome. We merged isoforms identified across all samples with gffcompare v0.12.10 (https://github.com/gpertea/gffcompare/). Non-redundant isoforms found only in the control samples or only in the si*RbFox2* samples were partitioned out for visualization in the UCSC Genome Browser. To validate *Actn1* splice isoforms, total RNA was isolated from A7R5 cells transfected with *RbFox2* siRNA and matched scrambled control siRNA, and 250 ng of RNA was reverse-transcribed. The resulting cDNA was diluted to 1:4 and PCR-amplified. Reactions were performed in duplicates on an Eppendorf Flexlid Mastercycler (Eppendorf SE) and contained 200 ng of cDNA, 0.5 μL of each primer, 2 mM dNTPs (R0192, Thermo Fisher Scientific), and 1.25 U DreamTaq DNA polymerase (EP0702, Thermo Fisher Scientific). Primer sequences are shown in Supplemental Figure 12. The amplified PCR products were gel-extracted (28704, QIAquick Gel Extraction Kit, QIAGEN) and analyzed by Sanger sequencing (Functional Biosciences). Sanger-sequenced amplicons were matched and annotated by the reference genome in the Rat Genome Database (42, 43).

### Statistics.

All data are presented as mean ± SEM. Parametric analyses were used throughout, including 2-way ANOVA with repeated measures and followed by selected (Šidák’s) or all pairwise (Tukey’s) multiple-comparison procedures, 2-tailed *t* test, or 1-tailed *t* test. A *P* value of less than 0.05 was considered statistically significant. Outliers were identified by Grubb’s test and are clearly indicated in the Supporting Data Values file.

### Study approval.

All protocols were approved by the Animal Care and Use Committee at the Medical College of Wisconsin. Surgical and experimental procedures adhered to the National Institutes of Health’s *Guide for the Care and Use of Laboratory Animals* (National Academies Press, 2011).

### Data availability.

Values for all data points in graphs are reported in the Supporting Data Values file. A file with raw blots is provided as supplemental material. MS proteomics data were deposited to the ProteomeXchange Consortium via the PRIDE partner repository with the dataset identifiers PXD067797 and 10.6019/PXD067797. Iso-Seq data were deposited at the NCBI’s Gene Expression Omnibus (GEO) with the accession number GSE305901. The data underlying this article will be shared upon reasonable request and have been deposited at the Harvard Dataverse under the “Curt Sigmund Laboratory Dataverse.”

## Author contributions

GK designed research studies, conducted experiments, acquired and analyzed data, and wrote the manuscript. NC designed research studies, conducted experiments, acquired and analyzed data, and wrote sections of the manuscript. DTB conducted experiments, acquired and analyzed data, and assisted with editing the manuscript. DG conducted experiments and acquired and analyzed data. IV performed bioinformatics analysis of the proteomic data. KTL conducted experiments and acquired and analyzed data. KKW performed mouse husbandry. RKS provided the RbFox2^flox^ mouse model and advice on its use. CDS designed the research studies, acquired and analyzed data, provided reagents, and wrote and edited the manuscript.

## Conflict of interest

The authors have declared that no conflict of interest exists.

## Funding support

This work is the result of NIH funding, in whole or in part, and is subject to the NIH Public Access Policy. Through acceptance of this federal funding, the NIH has been given a right to make the work publicly available in PubMed Central.

- NIH grants HL084207, HL144807, HL177123 (to CDS); AR083158 (to RKS).
- The Advancing a Healthier Wisconsin Endowment grants AHW5520631 (to CDS) and AHW5520747 (to GK).
- American Heart Association grant 15SDG25610021 (to RKS).
- Medical College of Wisconsin Cardiovascular Research Center (to GK).

## Supplementary Material

Supplemental data

Supplemental data set 1

Supplemental data set 2

Unedited blot and gel images

Supporting data values

## Version 1 04/02/2026

In-Press Preview

## Version 2 06/08/2026

Electronic publication
