# Supplementary material for: Vascular smooth muscle RbFox2 regulates the cytoskeleton and arterial stiffness by a RhoBTB1/Cullin-3 mechanism
Source: JCI Insight. 2026 Apr 2;11(11):e202638. doi: 10.1172/jci.insight.202638 (PMC13313548; doi:10.1172/jci.insight.202638)
Supplement: Supplemental data [file jciinsight-11-202638-s101.pdf]

Supplemental Data

Vascular Smooth Muscle RbFox2 Regulates the Cytoskeleton and Arterial Stiffness by a RhoBTB1-Cullin-3 Mechanism

Gaurav Kumar[1], Nisita Chaihongsa[1], Daniel T. Brozoski[1], Daria Golosova[1], Ibrahim Vazirabad[1], Ko-Ting Lu[1], Kelsey K. Wackman[1], Ravi K. Singh[2] and Curt D. Sigmund[1]

[1]Department of Physiology, Cardiovascular Center, Medical College of Wisconsin, Milwaukee, WI, USA, 53226

[2]Department of Pharmacological and Pharmaceutical Sciences, College of Pharmacy, University of Houston, Houston, TX, USA, 77204

*Corresponding Author:
Curt D. Sigmund, Ph.D.
Department of Physiology
Medical College of Wisconsin
8701 Watertown Plank Road
Milwaukee, WI 53226-0509
Phone: 414-955-8277
E-mail: csigmund@mcw.edu

Supplemental Figures and Legends

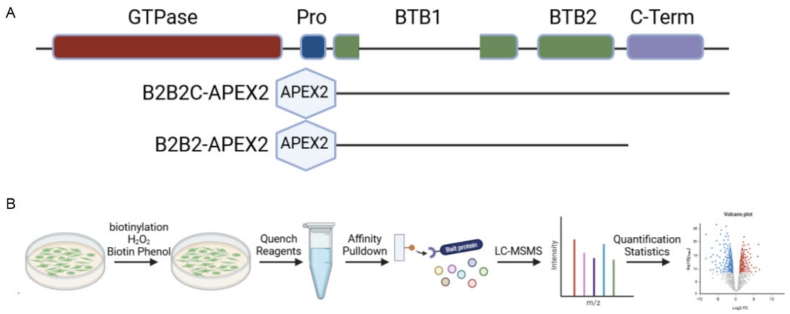

Figure S1. *Schematic of APEX2 Proximity Labeling in A7R5 SMCs.* A) Architecture of APEX2-tagged B1B2C and B1B2 domains of RhoBTB1 for proximity labelling of SMC proteome. B) Strategy to biotinylate the SMC proteome by APEX2 tagged RhoBTB1 domains. Biotinylation involved transfection of A7R5 cells with APEX2-tagged B1B2 and B1B2C domains, treatment with biotin phenol and H_2O_2, affinity pulldown of SMC proteome with streptavidin beads followed by mass spectrometry (MS)-assisted proteome identification and pathway enrichment analysis. Created in BioRender with an academic license.

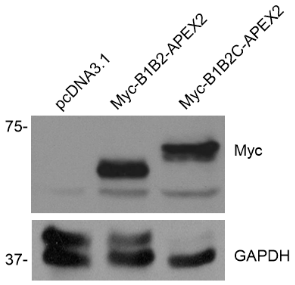

Figure S2. *Optimization of APEX2-tagged RhoBTB1 Domain Expression.* Immunoblot demonstrating the expression of Myc and APEX2-tagged B1B2 and B1B2C domains in A7R5 SMCs for 24 hours. Cells in the control lane were transfected with pcDNA3.1 empty vector. Immunoblots were probed with indicated anti-sera. GAPDH was used as loading control. Molecular weight markers are translated from original blots.

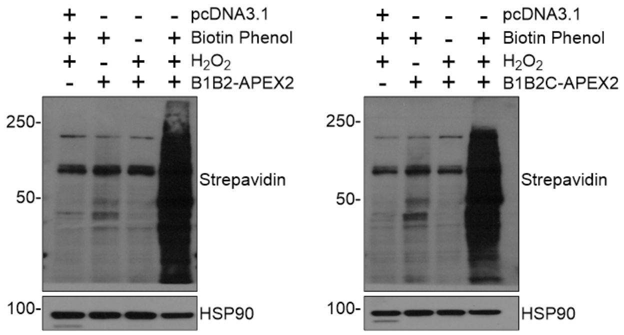

Figure S3. *Characterization of APEX2-tagged Constructs for Functional Activity.* Immunoblots showing robust biotinylation of the A7R5 proteome following transfection with APEX2-tagged constructs and treatment with H_2O_2 and biotin phenol. No biotinylation was evident in the absence of either of these components. Cells in the control lane were transfected with pcDNA3.1 empty vector and treated with appropriate substrates. Immunoblots were probed with streptavidin HRP-conjugate. HSP90 was used as loading control.

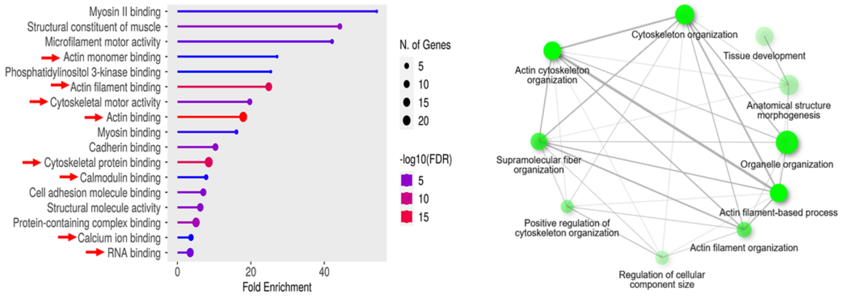

Figure S4. *Pathway Enrichment Analysis of A7R5 proteome.* Mass-spectrometry (MS) analysis of the A7R5 proteome identified several proteins prominently associated with pathways regulating cytoskeletal organization as shown by pathway enrichment analysis (left, red arrows). These proteins modulate actin-dependent cellular events critical for cytoskeletal dynamics (right).

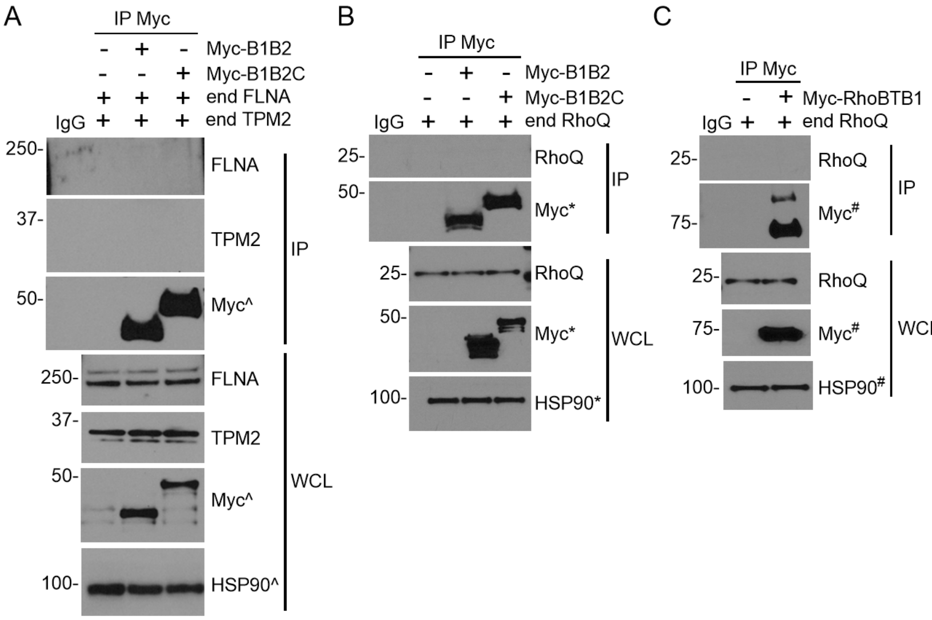

Figure S5. *Failed Validation of Several RhoBTB1 Candidates.* A and B) Co-immunoprecipitation (Co-IP) assay was performed with myc-epitope tagged RhoBTB1 domains, and C) myc-epitope tagged full-length RhoBTB1 transfected into A7R5 SMCs using anti-myc beads or isotype-matched IgG as control. In all Co-IP assays, cells in the control lane were transfected with pcDNA3.1 empty vector and were treated with MLN4924 for 16 hours and MG132 for the last 4 hours, prior to lysis. Total transfection time was 24 hours. Immunocomplexes were western blotted with indicated anti-sera. Molecular weight markers are translated from original blots. Data is representation of two independent replicates. IP represents the immunoprecipitation; WCL represents whole cell lysates. The (*), (^), and ($^{\#}$) notations indicate that the blots are identical to those in Figures 2 and 3 as those blots were from the same contemporaneously performed experiment.

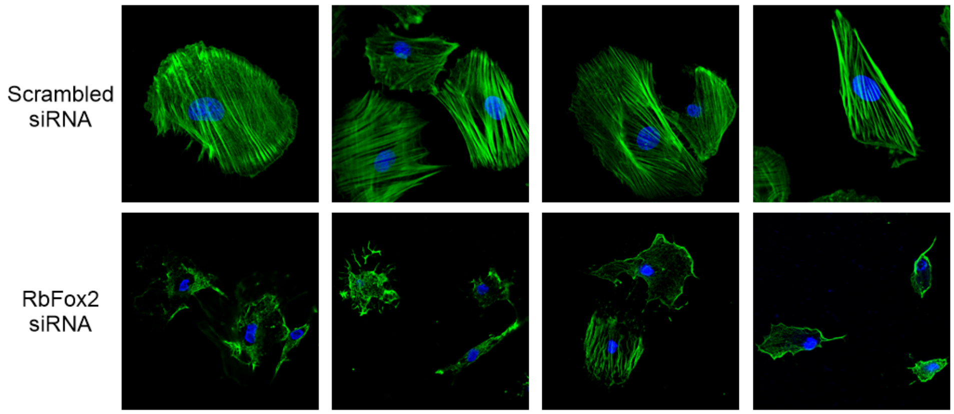

Figure S6. *Cytoskeletal Modulation.* Additional examples of Phalloidin staining (green) in A7R5 cells following RbFox2 siRNA transfection (72 hours) compared to scrambled control. DAPI as counter stain in confocal images shows nuclei.

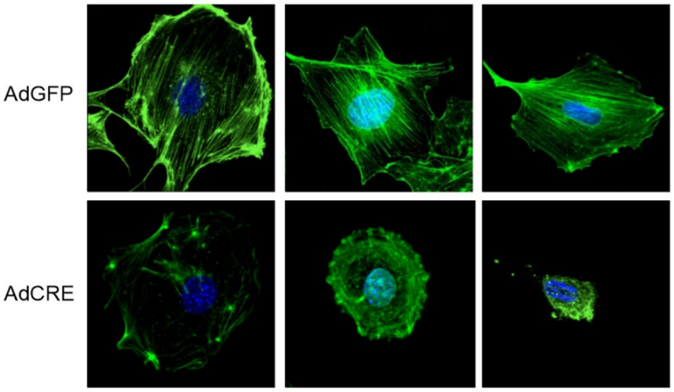

Figure S7. *Cytoskeletal Modulation.* Additional examples of Phalloidin staining (green) in primary SMCs from RbFox2$^{Flox/Flox}$ mice infected with either AdGFP (control) or AdCRE (RbFox2 deletion). DAPI as counter stain in confocal images shows nuclei.

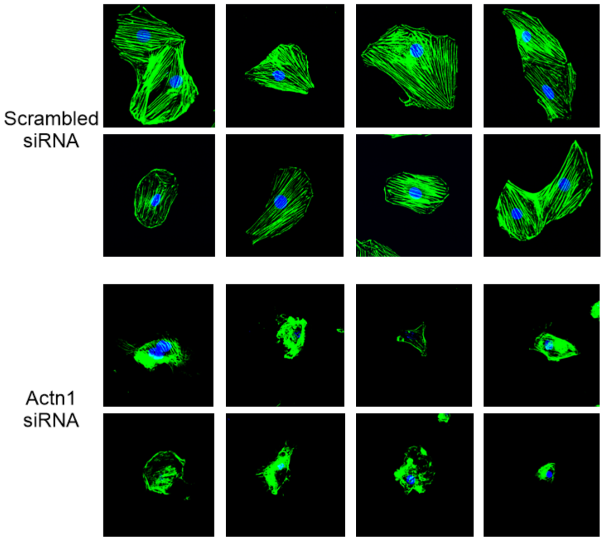

Figure S8. *Cytoskeletal Modulation.* Additional examples of Phalloidin staining (green) in A7R5 cells following Actn1 siRNA transfection (72 hours) compared to scrambled control. DAPI as counter stain in confocal images shows nuclei.

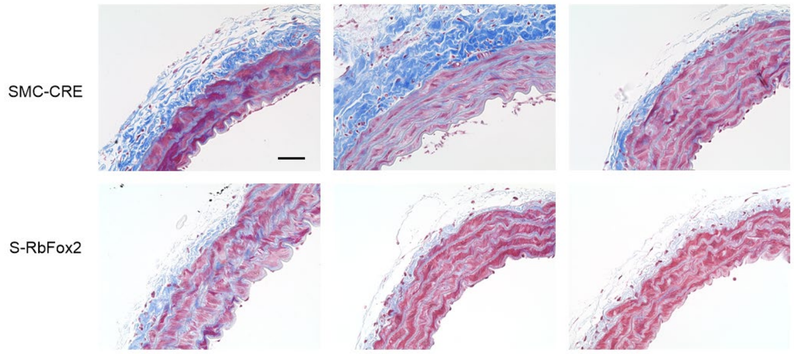

Figure S9. *Masson's-Trichrome Stain of Aorta.* Additional representative Masson's-Trichrome staining of SMC-CRE and S-RbFox2 mice aorta cross-sections showing collagen deposition 3-weeks after ANG treatment followed by Tamoxifen (Tx) injections. Scale bar = 50 μm.

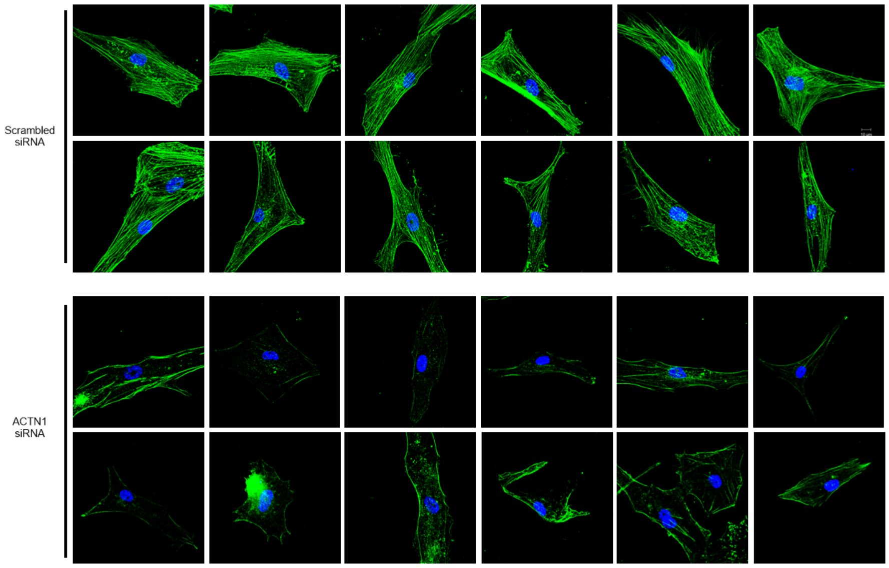

Figure S10. *Cytoskeletal Modulation.* Additional examples of Phalloidin staining (green) in HASMC cells following *Actn1* siRNA transfection (72 hours) compared to scrambled control. DAPI as counter stain in confocal images shows nuclei.

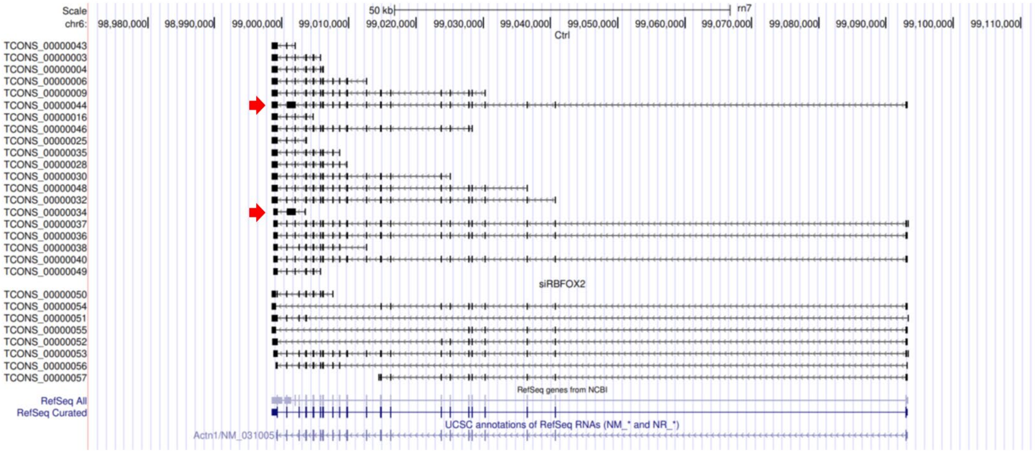

Figure S11. *Alignment of Actn1 to the Rat Genome*. The figure shows a screenshot of a custom track generated in the UCSC Genome browser using mRatBN7.2/rn7 version of the rat genome sequence. Unique mRNA isoforms identified in the IsoSeq analysis are shown. Red arrowheads point to mRNAs scored in Figure S10.

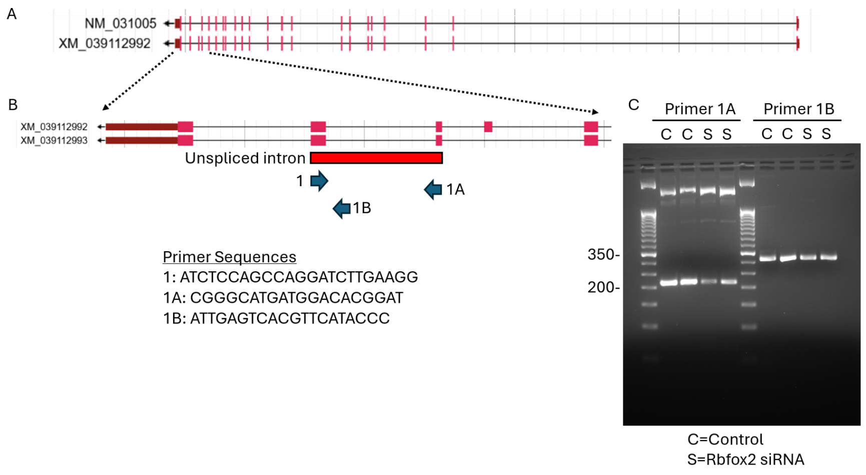

Figure S12. *Validation of Actn1 Splicing*. A) Schematic of the Actn1 locus generated in the Rat Genome Database Browser (JBrowse 1) using mRatBN7.2 version of the rat genome sequence. Note: the direction of transcription is from right to left. B) The region at the 3' end of the gene is expanded showing the presence of the unspliced intron (intron inclusion) from Figure S9 and the locations and sequence of the RT-PCR primers. C) RT-PCR on RNA from control (C) and RbFox2 siRNA (S) treated A7R5 cells. Size markers are identified. Primer set 1A amplified the correctly spliced mRNA. Primer set 1B amplified the mRNA with the intron included. PCR products were verified by DNA sequencing.

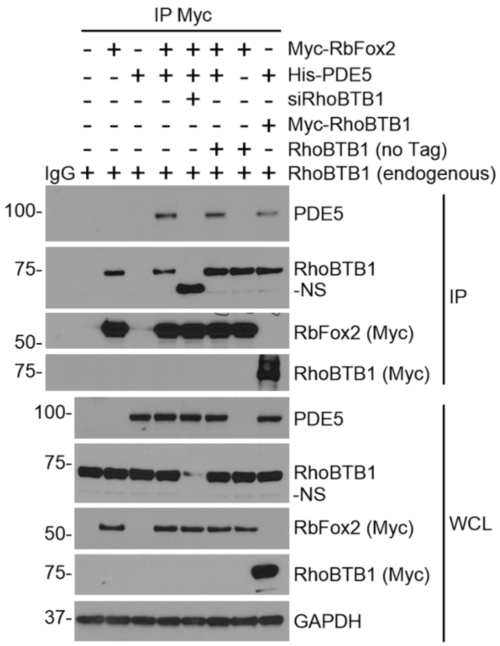

Figure S13. *PDE5 and RbFox2 Do Not Directly Interact.* Co-immunoprecipitation (Co-IP) assay was performed with RhoBTB1 (tagged or untagged), PDE5 (His-tagged) and RbFox2 (Myc tagged) in HEK293 cells using anti-myc beads or isotype-matched IgG as control. In all Co-IP assays, cells in the control lane were transfected with pcDNA3.1 empty vector and were treated with MLN4924 for 16 hours and MG132 for the last 4 hours, prior to lysis. Total transfection time was 24 hours. Immunocomplexes were western blotted with indicated anti-sera. Molecular weight markers are translated from original blots. Data is from a single experiment. IP represents the immunoprecipitation; WCL represents whole cell lysates.

Table S1: Profiling of the A7R5 RhoBTB1 Proteome

Protein	B1B2C/B1B2	Adjusted P	Protein	B1B2C/B1B2	Adjusted P
Eif3e	1.501	0.031734	Cfh	1.54	0.003291
Actr3	1.501	0.037397	Dnm2	1.55	0.00026
Pls3	1.508	0.035133	Flnc	1.594	0.0000117
Dync1h1	1.547	0.007736	Myh9	1.613	0.0000754
Ywhaz	1.561	0.036436	Sept7	1.626	0.001771
Abcc4	1.573	0.016606	Map1b	1.64	0.000695
Mprip	1.595	0.023219	Clcc1	1.675	0.003434
Abce1	1.603	0.029861	Gsn	1.679	0.000116
Rrbp1	1.626	0.024503	Myl6	1.72	0.00026
Cap1	1.631	0.041784	Marc2	1.754	0.003458
Cbx3	1.636	0.022309	Txndc15	1.771	0.00488
Mia3	1.652	0.014493	Rlca	1.812	0.0000149
Nkap	1.663	0.014238	Rai14	1.859	0.000455
Fhl2	1.664	0.005017	Flna	1.862	0.000000276
Lars1	1.681	0.031412	Cald1	1.89	0.00614
Ywhag	1.748	0.046156	Fndc3b	1.927	0.000177
Pofut1	1.79	0.022213	Tmem263	1.932	0.000948
Myoc1	1.791	0.021058	Actn1	1.947	0.0000149
Rpl35	1.854	0.0096	Otol1	1.974	0.001458
Hibadh	2.03	0.005525	Rars1	2.065	0.0000135
Cdk1	2.19	0.030075	Flnb	2.073	0.000462
Luzp1	2.342	0.035526	Tmp4	2.105	0.00415
Tpm1	2.4	0.004915	Myh10	2.11	0.00000726
Tmem106b	2.453	0.01231	Lima1	2.296	0.001449
Sprr2d	2.558	0.043794	Tpm2	2.438	0.003349
Sec61b	3.23	0.012749	Atp5f1d	2.661	0.00395
Slc12a2	3.281	0.005492	Stoml2	2.825	0.000664
Rpl8	1.532	0.0000117	Tpm3	2.898	0.000927
Es1	8.993	0.007732	Myl3	4.863	0.00025

Candidate proteins selected by applying a cut-off interaction of B1B2C/B1B2 >1.5-fold and statistical significance (P<0.05 adjusted by Benjamini-Hochberg procedure).

Table S2. Chromatography and MS Instrument Acquisition Settings.

Sample Volume	20 µL	Isolation Window	1.6 m/z
Stationary Phase	Thermo Acclaim PepMap C18 75µm × 50cm	MS2 AGC Target	5e4
LC Solvent A	100% H2O, 0.1% formic acid	MS2 Maximum IT	54 ms
LC Solvent B	80% acetonitrile, 0.1% formic acid	Normalized Collision Energy	30
Gradient Ramp and Duration Flow Rate	2.5-5% B in 1 minute<br>5-7% B in 4 minutes<br>7-28% B in 72 minutes<br>28-60%B in 10 minutes<br>60-99%B in 4 minutes<br>300 nL/min	Minimum Intensity Req.	5e5
Mass Spectrometer	Thermo Orbitrap Fusion Lumos	Dynamic Exclusion	60.0 s
Spray Voltage	2.1 kV	MS2 acquisition	Data dependent, 3 s cycle time, Centroid
In-Source CID	0.0 eV	MS2 Fragmentation	HCD
MS1 scan range	375-1500 m/z	MS2 Detection	Orbitrap
MS1 resolution	120,000 @ 200 m/z	MS2 fixed first mass	110 m/z
MS1 AGC Target	4e5	MS2 resolution	30,000 @ 200 m/z
MS1 Maximum IT	50 ms	Advanced Precursor Determination	on

Table S3. Mass Spectrometry Data Processing Parameters

Platform	ProteomeDiscoverer 2.4	Quantitation	Precursor Ions Quantifier, Peak Area
Search Algorithms	SequestHT	Normalization	Total Peptide Amount
Validation	Percolator	Scaling	Validation
Database	Uniprot Rattus norvegicus with isoforms, 2022-04-30, MaxQuant Contaminants Recombinant sequence	Ratio Calculation	Summed Abundance Based
Digest	Trypsin (semi) 2 Missed Cleavages Allowed	Hypothesis Test	ANOVA (Individual Proteins)
Precursor mass tolerance	10 ppm	Target FDR (Strict) for PSMs:	0.01
Fragment mass tolerance	0.02 Da	Target FDR (Relaxed) for PSMs:	0.05
Static Modifications	Carbamidomethyl (C)	Target FDR (Strict) for Peptides:	0.01
Dynamic Modifications	Oxidation (M), Acetylation (protein N-terminus)	Target FDR (Relaxed) for Peptides:	0.05

Table S4: IsoSeq RNAseq Data for A7R5 Cells – Smooth Muscle Markers

Gene	Gene Description	A7R5 Counts (TPM)
Actn1	α1-actinin	1013
Acta2	Smooth muscle actin α2	10961
Actg2	Smooth muscle actin γ2	357
Cacna1c	Calcium voltage-gated channel α1C	15
Cald1	Caldesmon 1	1391
Cnn1	Calponin 1	480
Col1a1	Collagen type 1α1	5465
Col1a2	Collagen type 1α2	4730
Col3a1	Collagen type 3α1	2089
Eln	Elastin	35
Itga8	Integrin Subunit α8	20
Klf5	KLF Transcription Factor 5	31
Myh11	Myosin heavy chain 11	18
Myl9	Myosin light chain 9	204
Mylk	Myosin Light Chain Kinase	23
Pgm5	Phosphoglucomutase 5	352
Smtn	Smoothelin	106
Tagln	Transgelin	401
Tpm1	Tropomyosin 1	203
Tpm2	Tropomyosin 2	198
