# Supplementary material for: Vascular smooth muscle RbFox2 regulates the cytoskeleton and arterial stiffness by a RhoBTB1/Cullin-3 mechanism
Source: JCI Insight. 2026 Apr 2;11(11):e202638. doi: 10.1172/jci.insight.202638 (PMC13313548; doi:10.1172/jci.insight.202638)

# Full Unedited Blots

# Full unedited blots for Figure 2A

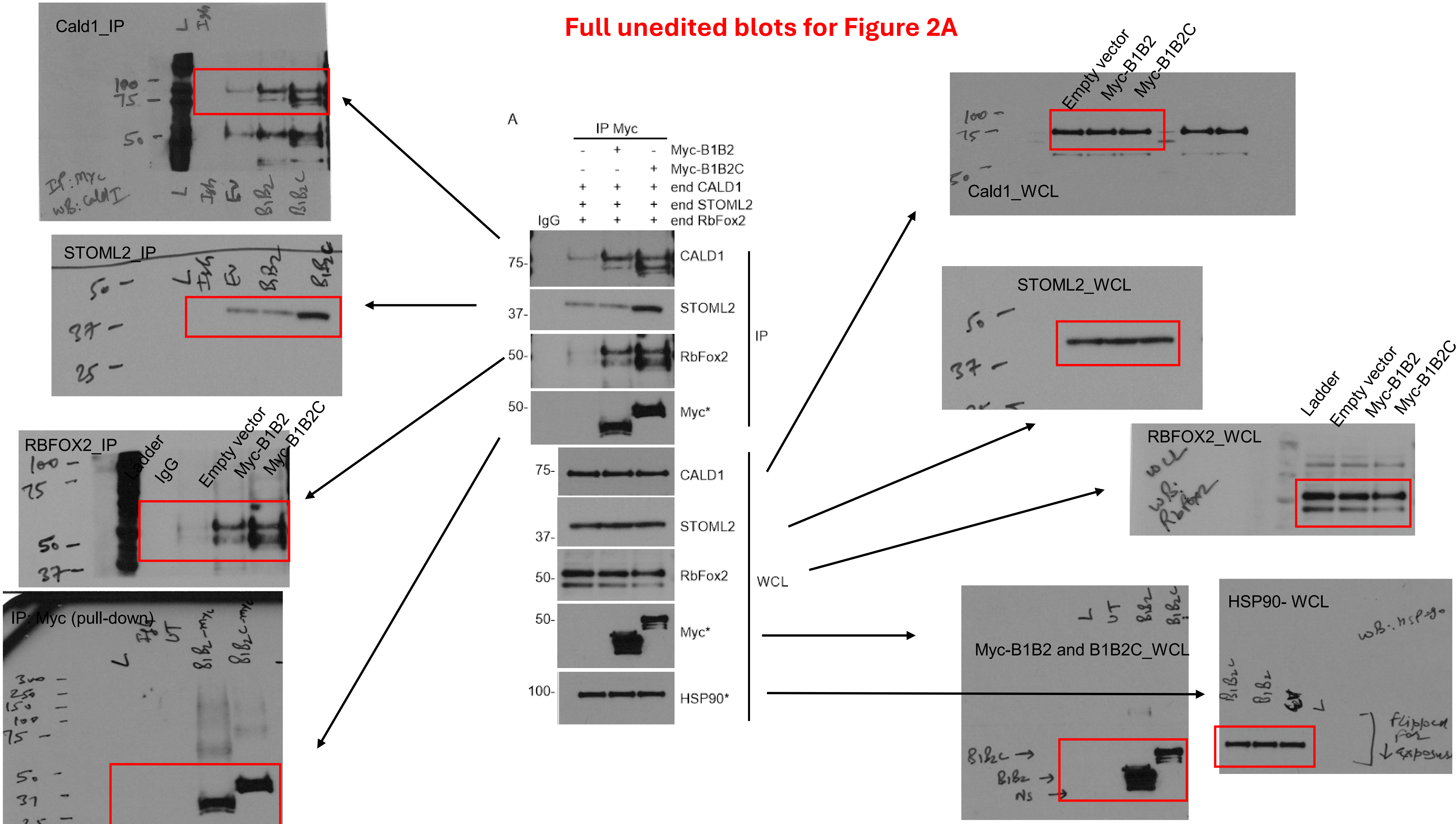

# Full unedited blots for Figure 2B

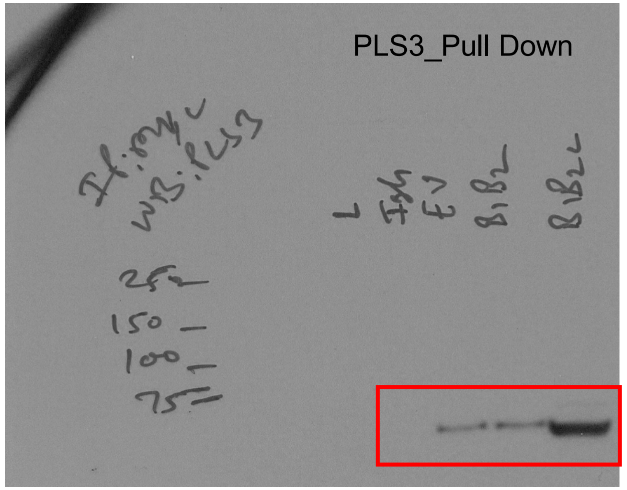


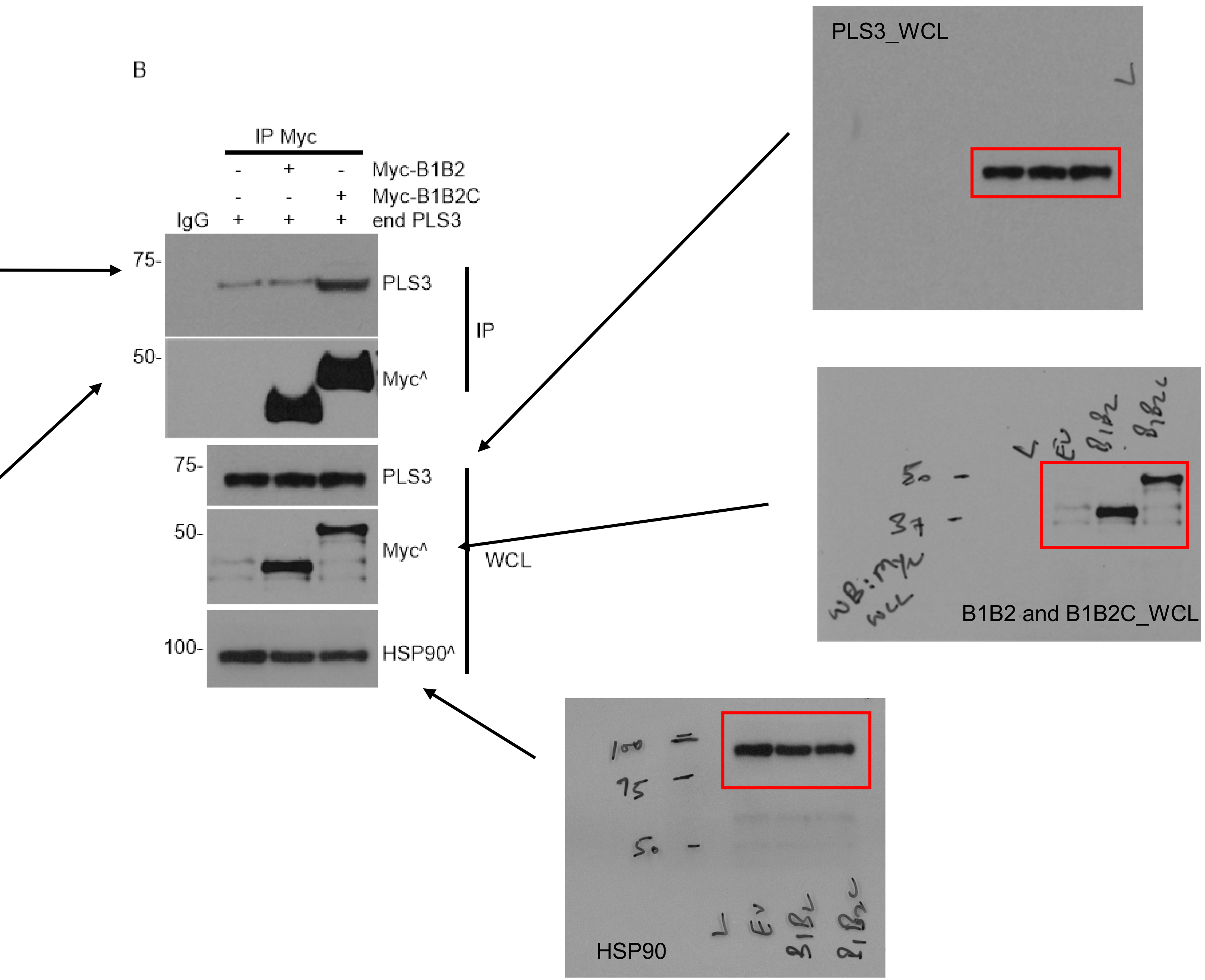


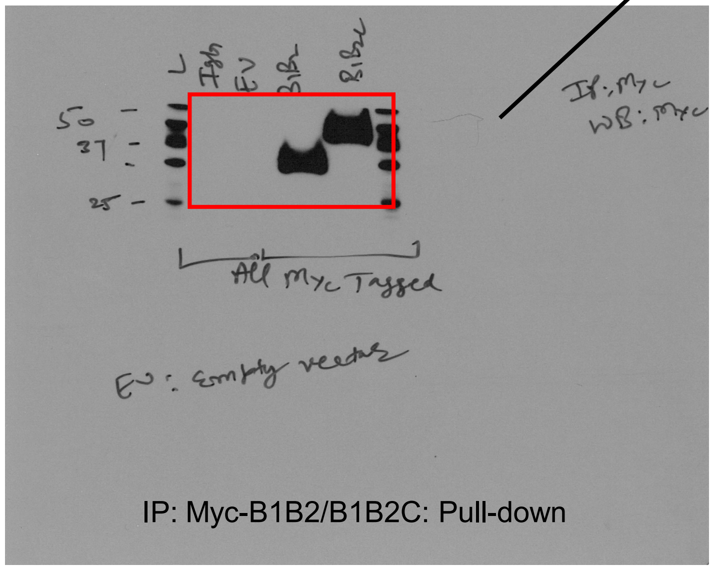

# Full unedited blots for Figure 3A

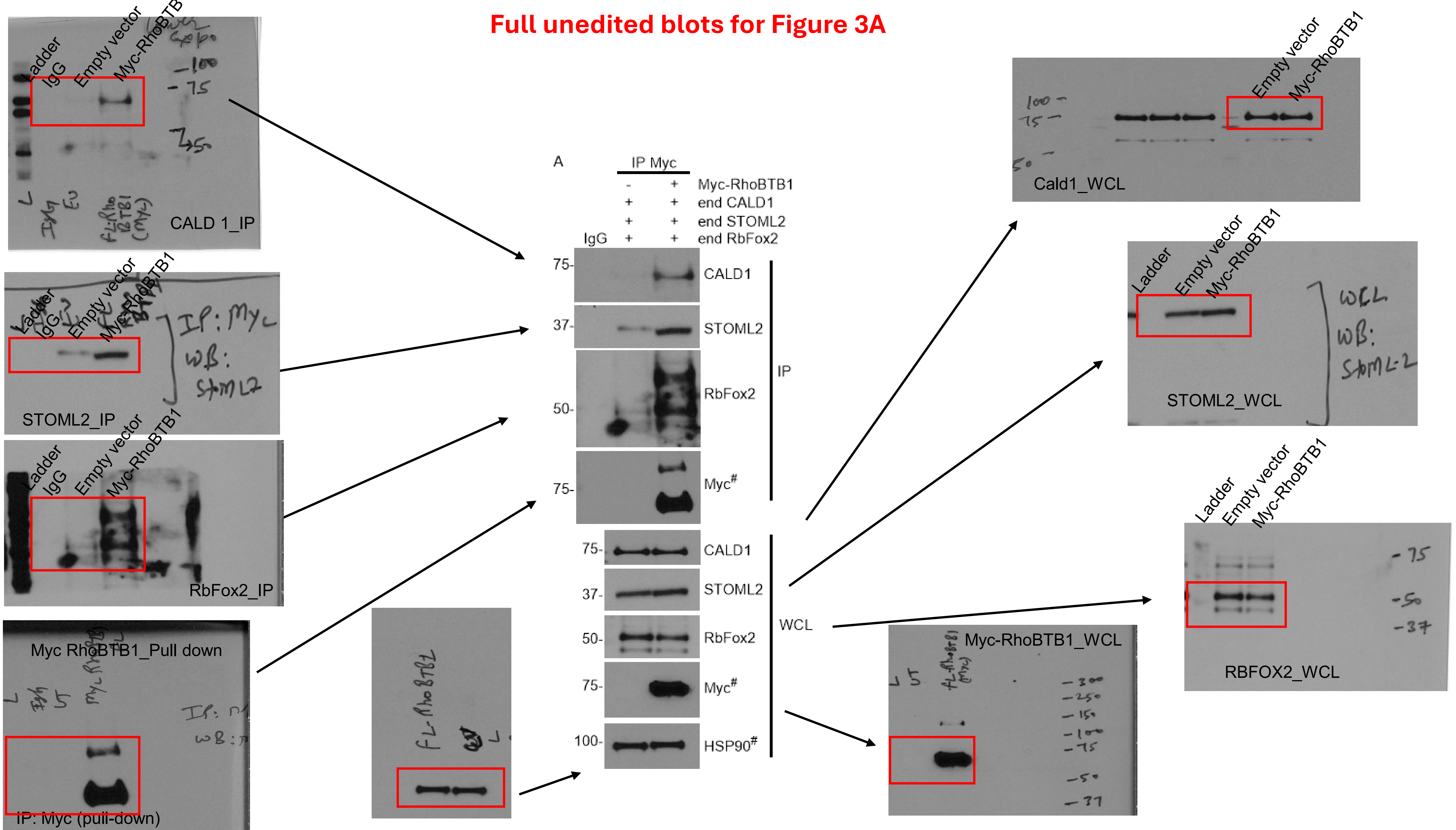

# Full unedited blots for Figure 3B

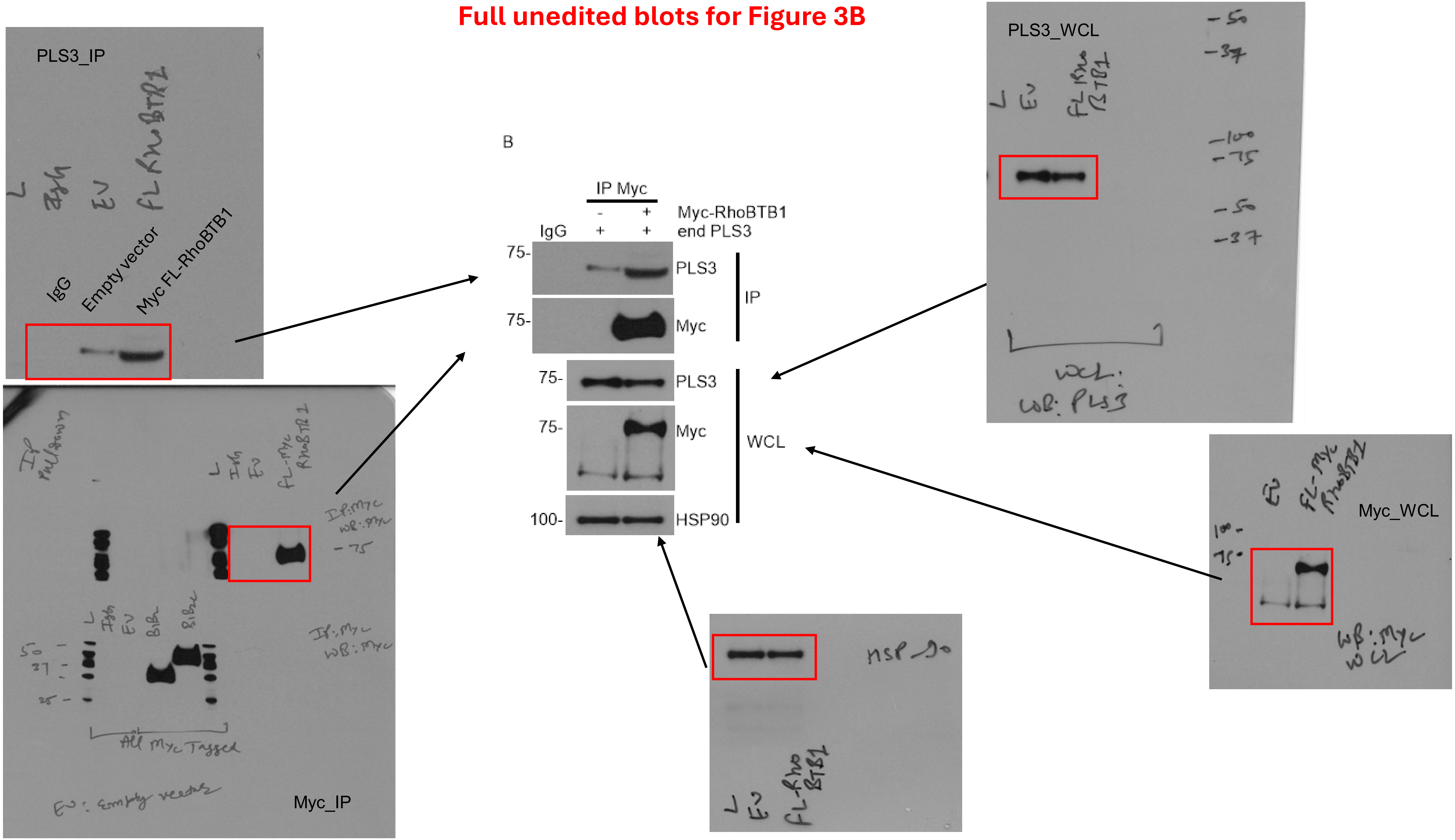

# Full unedited blots for Figure 4A

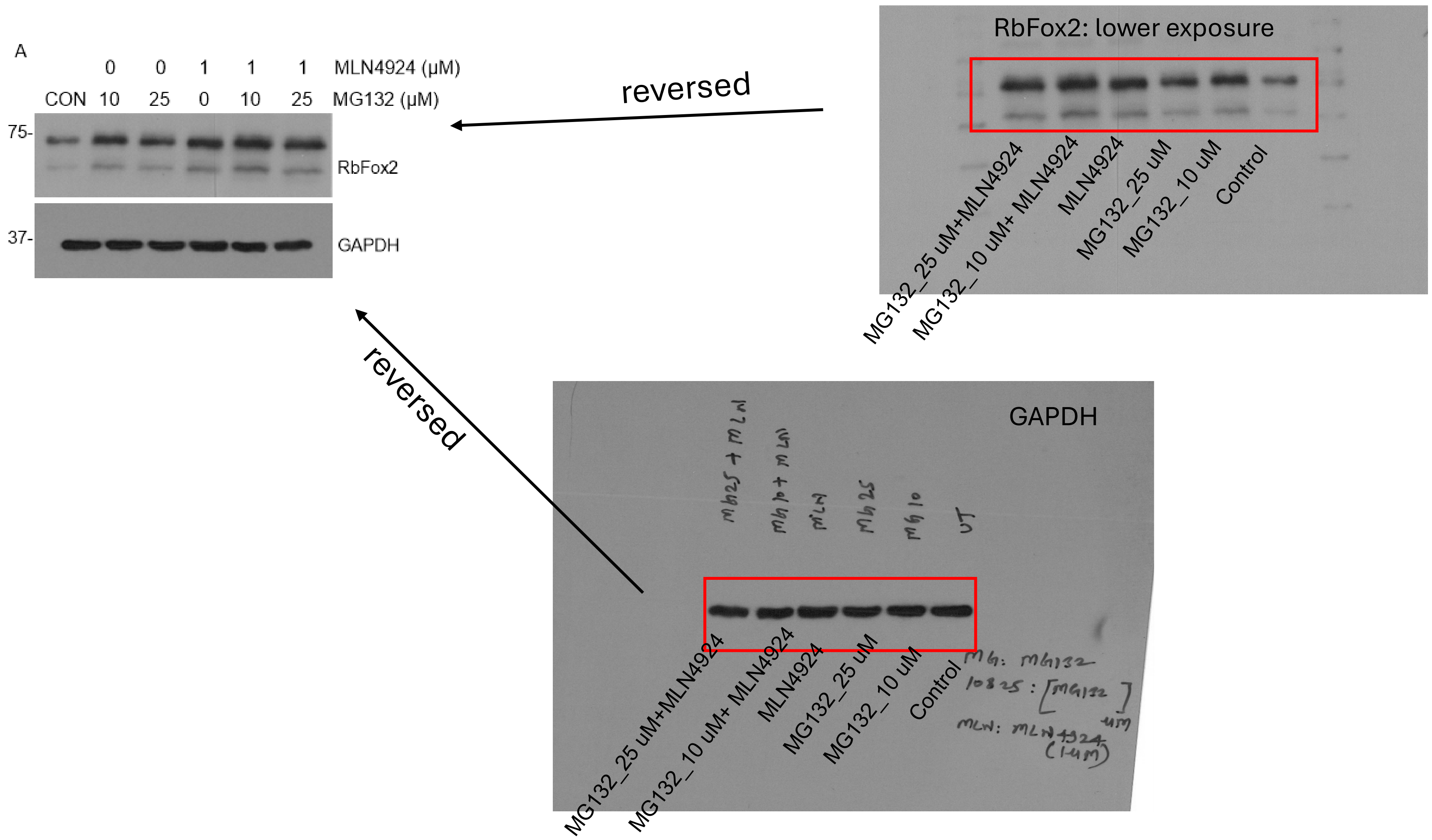

# Full unedited blots for Figure 4B

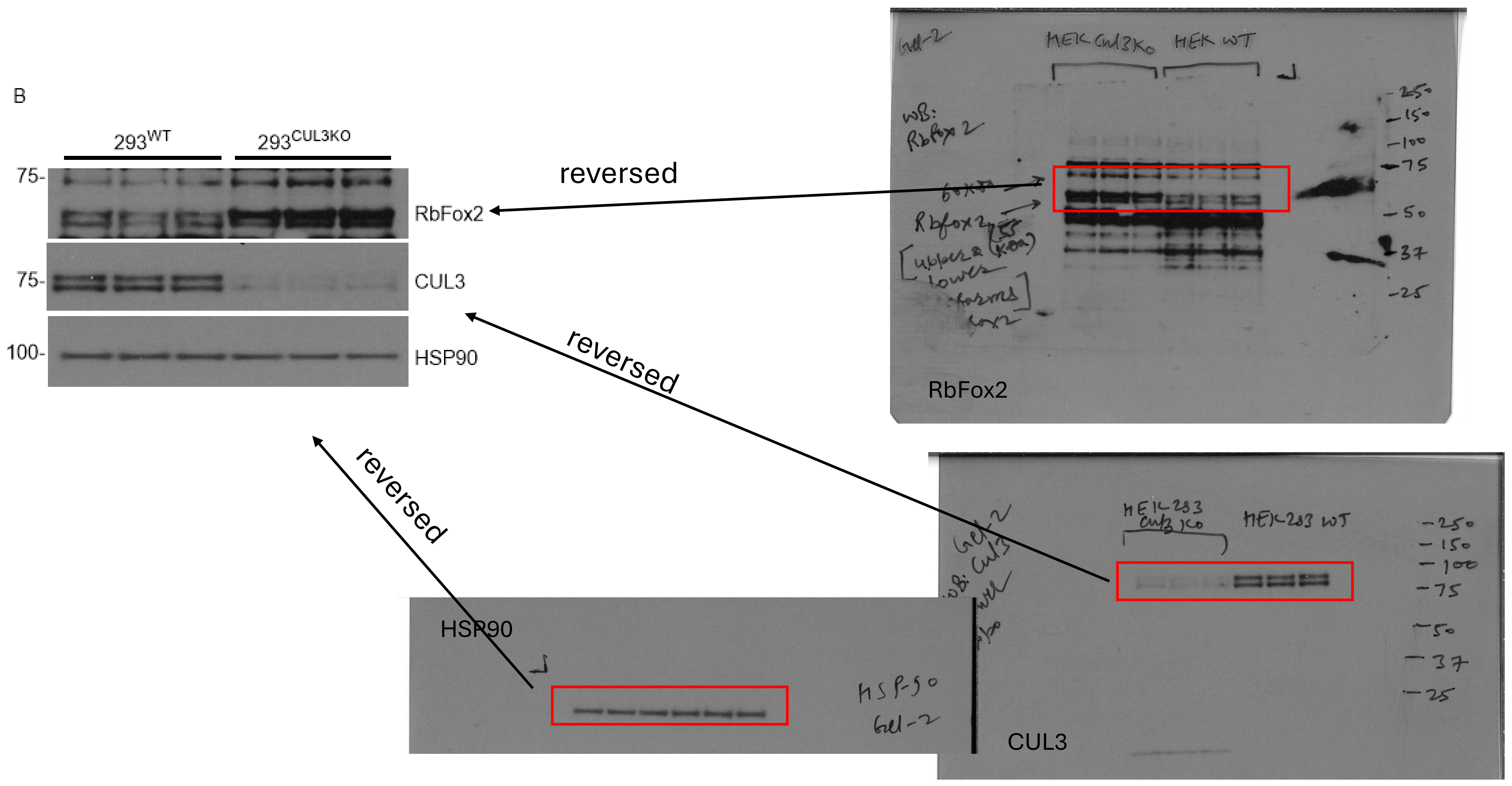

# Full unedited blots for Figure 4C

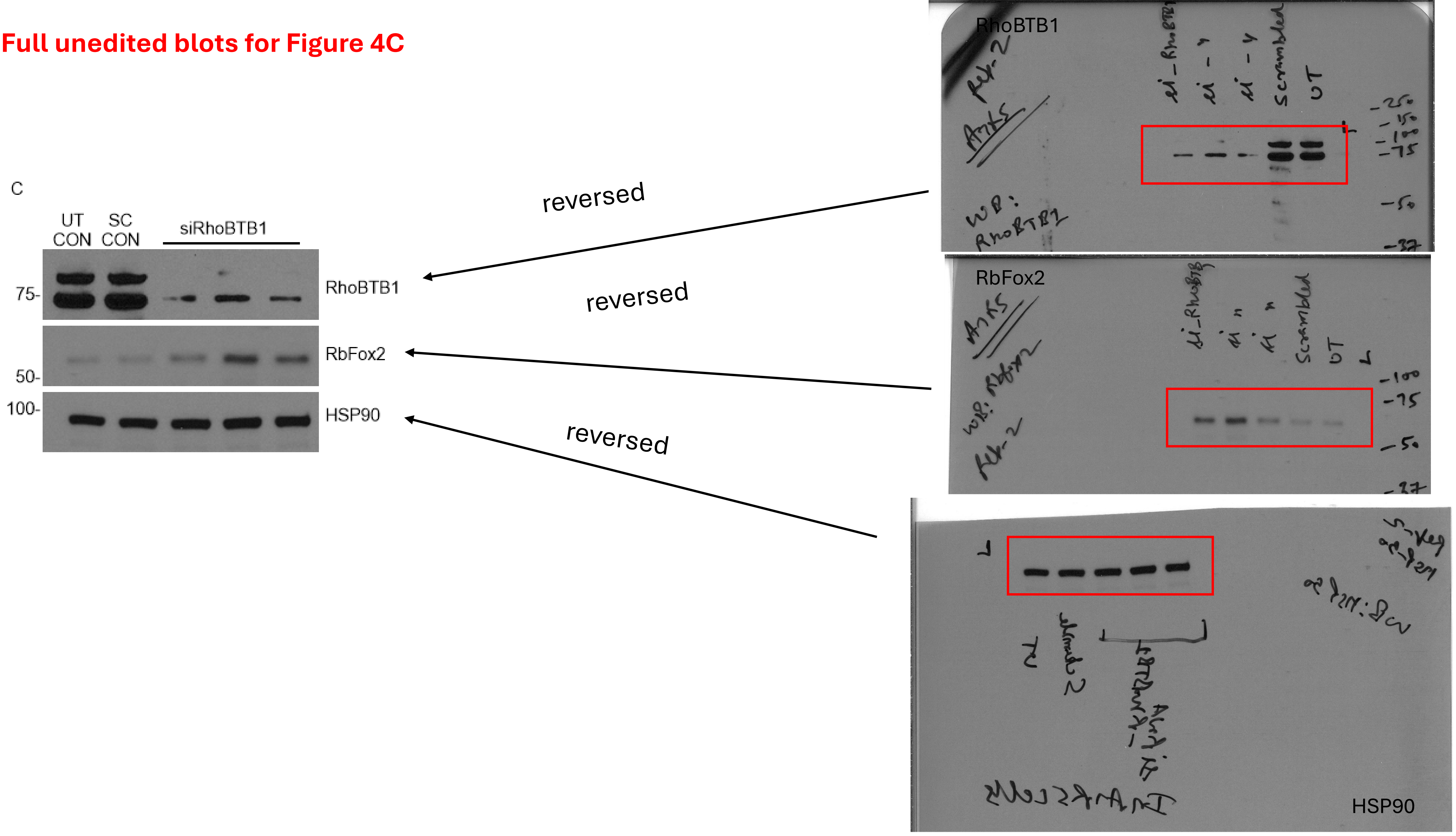

# unedited blots for Figure 5A

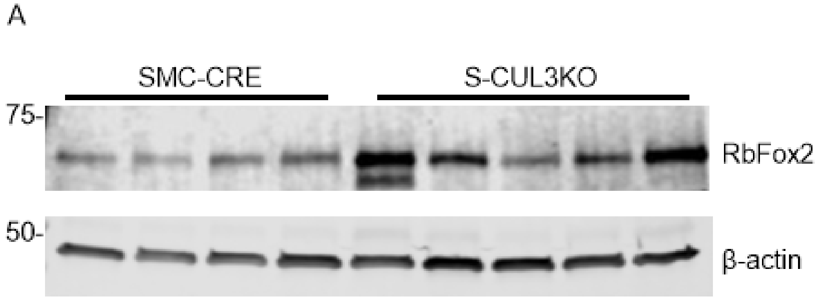


**These are the original blots, developed on Licor**

RbFox2

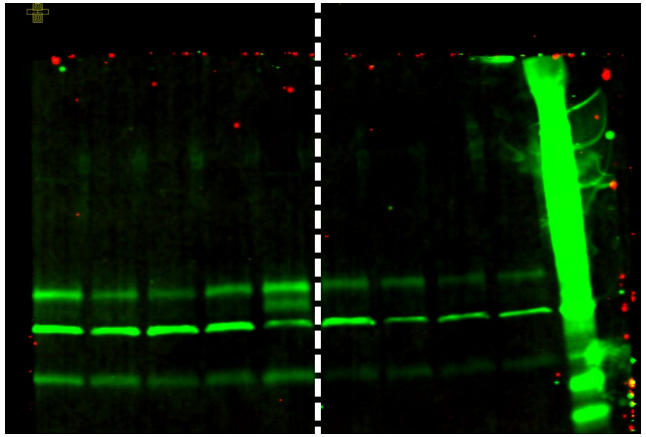

β-actin

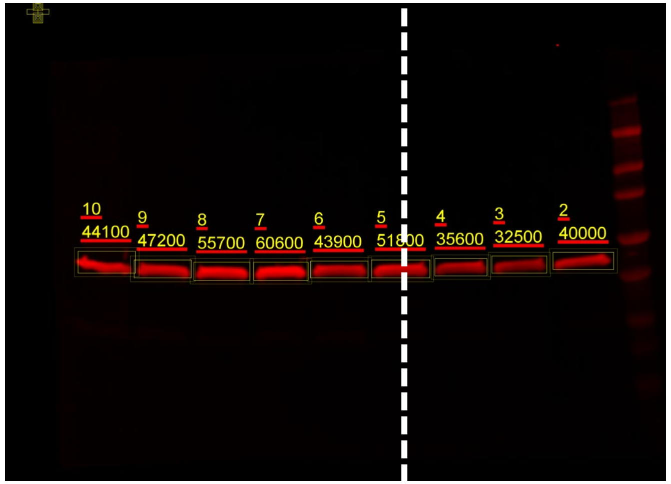


Set to gray Scale

Flipped for orientation

RbFox2, aorta

SMC-Cre

S-CUL3KO

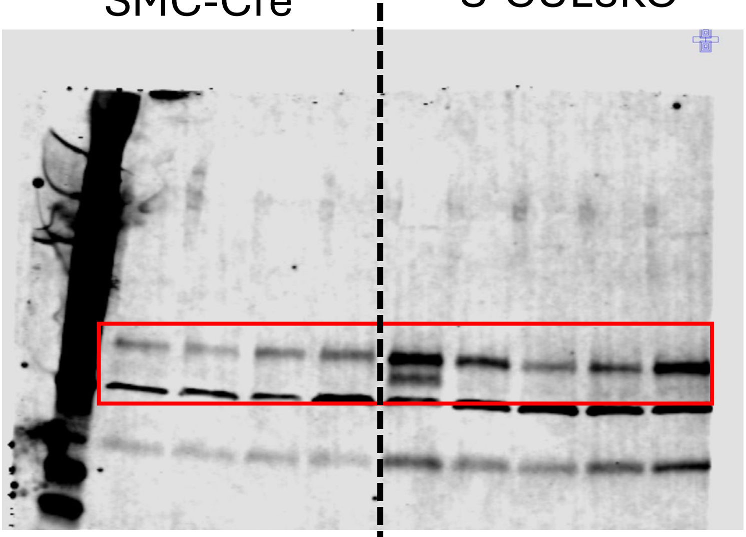

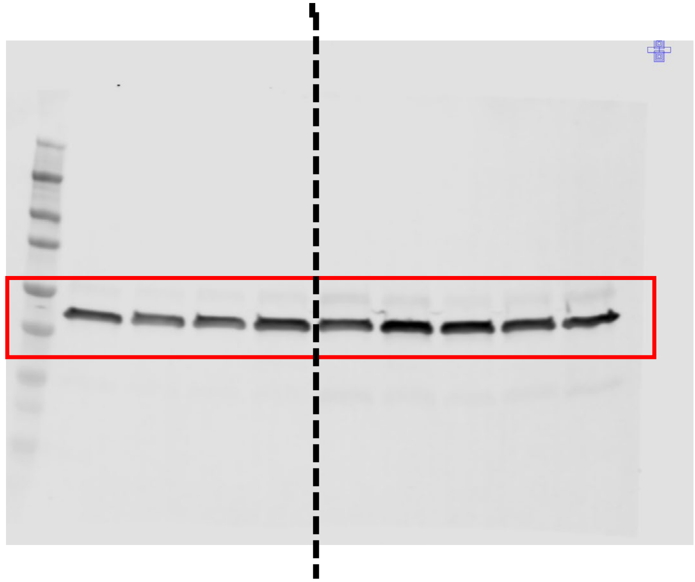

β-Actin, aorta

Note: These samples are the same as previously published.

A CUL3 blot from Ref 44 was re-probed with RbFox2.

The actin blot is the same as in Ref 44 as the samples were the same.

# Full unedited blots for Figure 5B

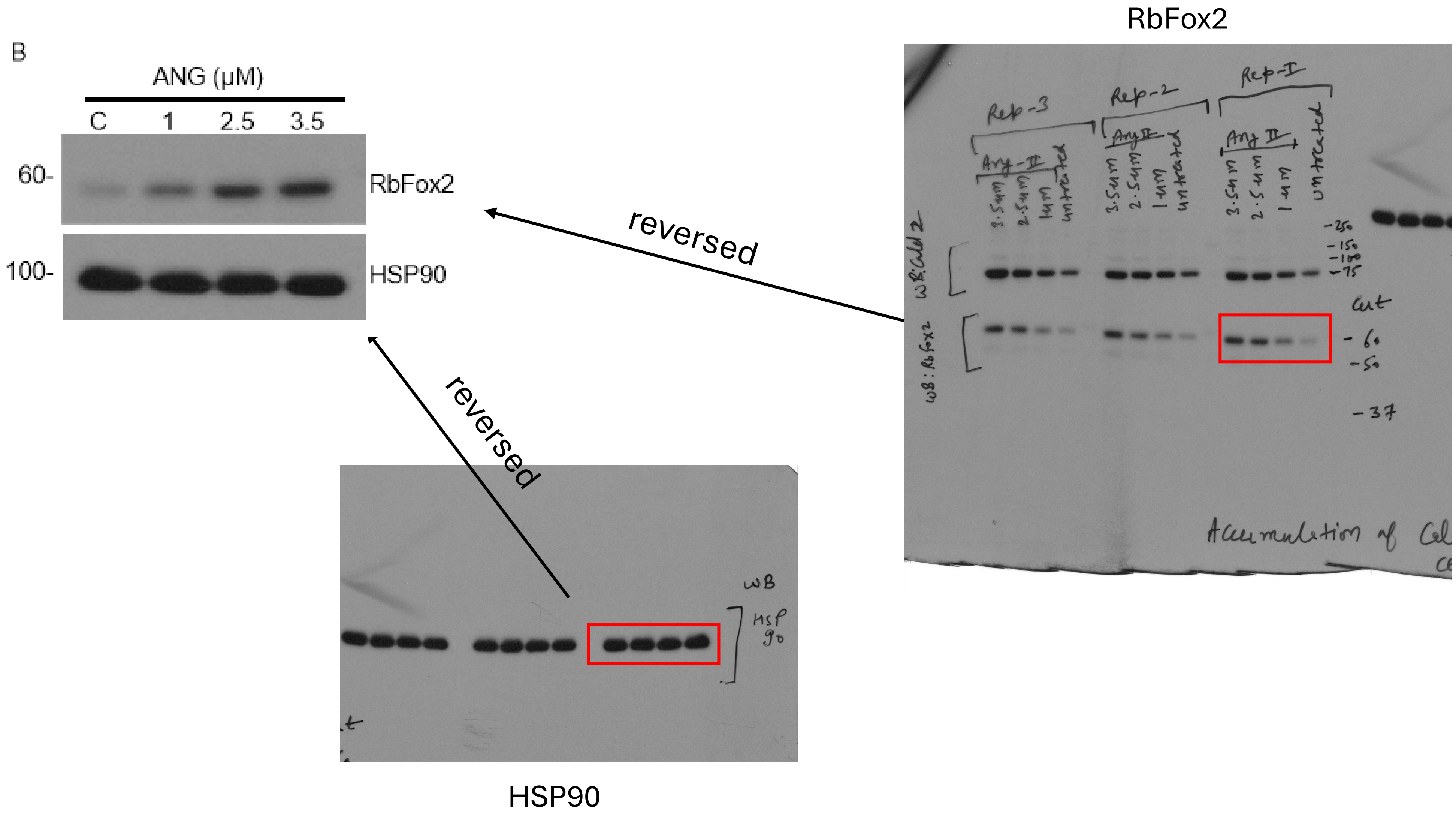

# Full unedited blots for Figure 5D

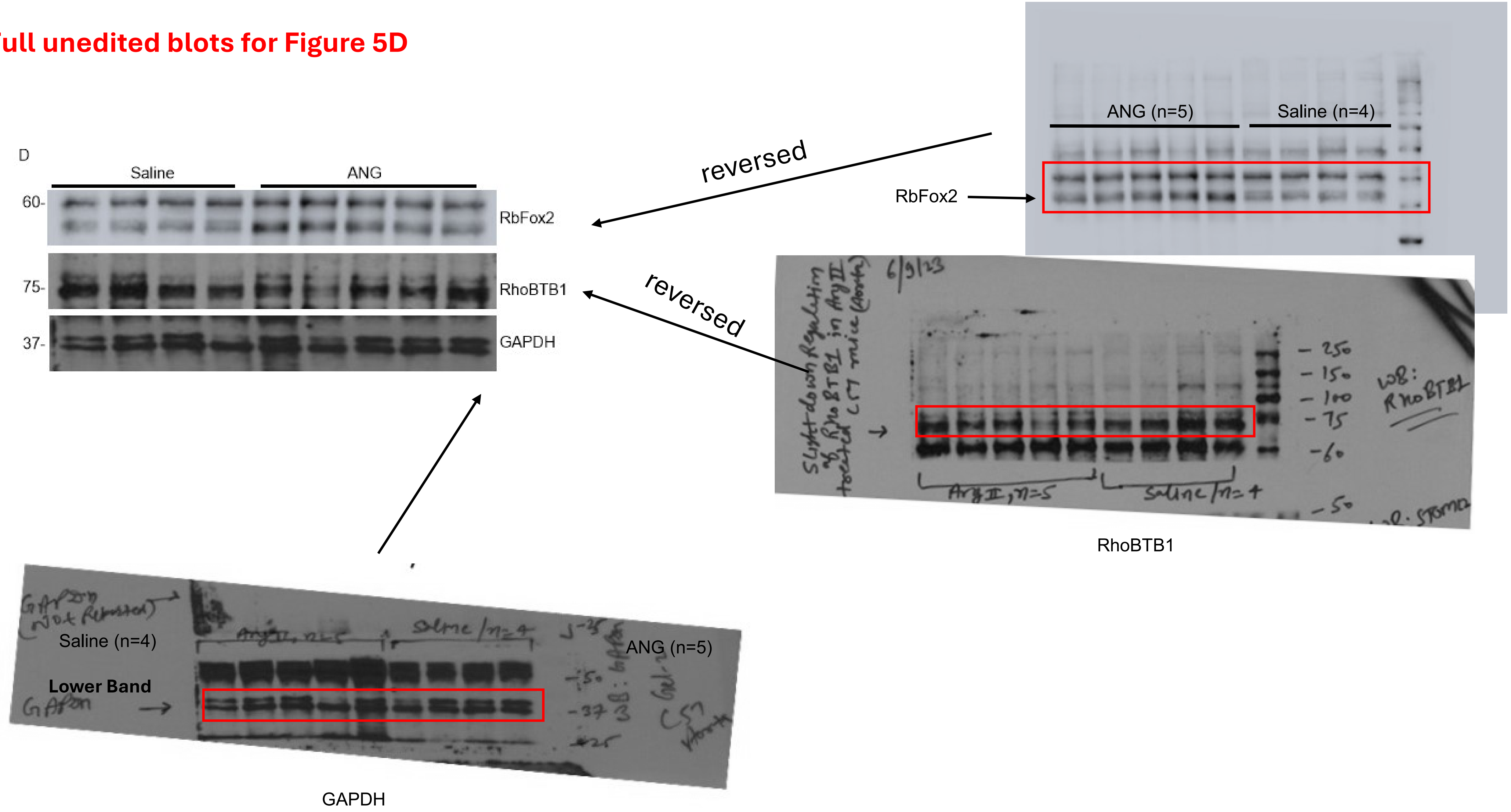

**Full unedited blots for Figure 6A**

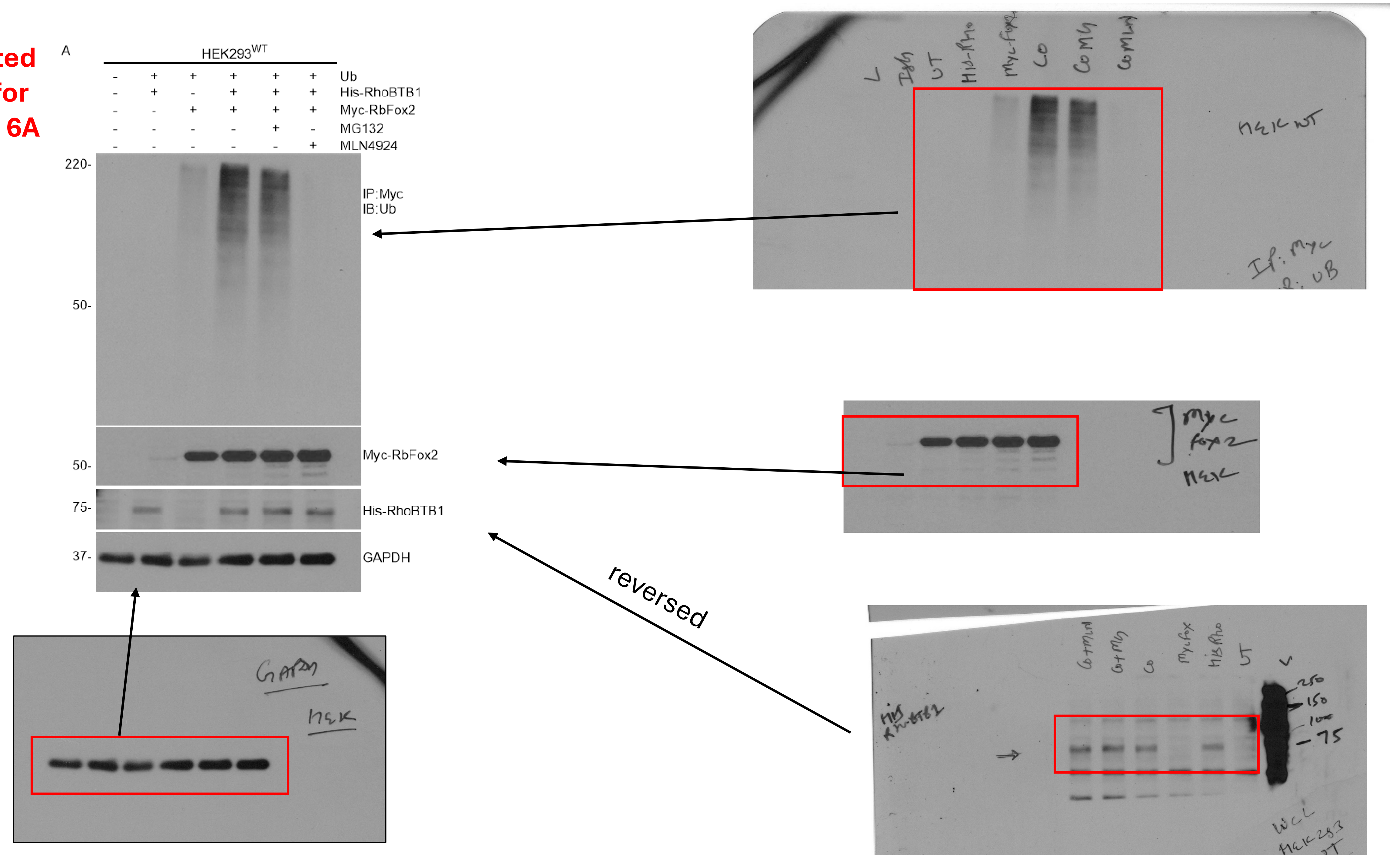

**Full unedited blots for Figure 6B**

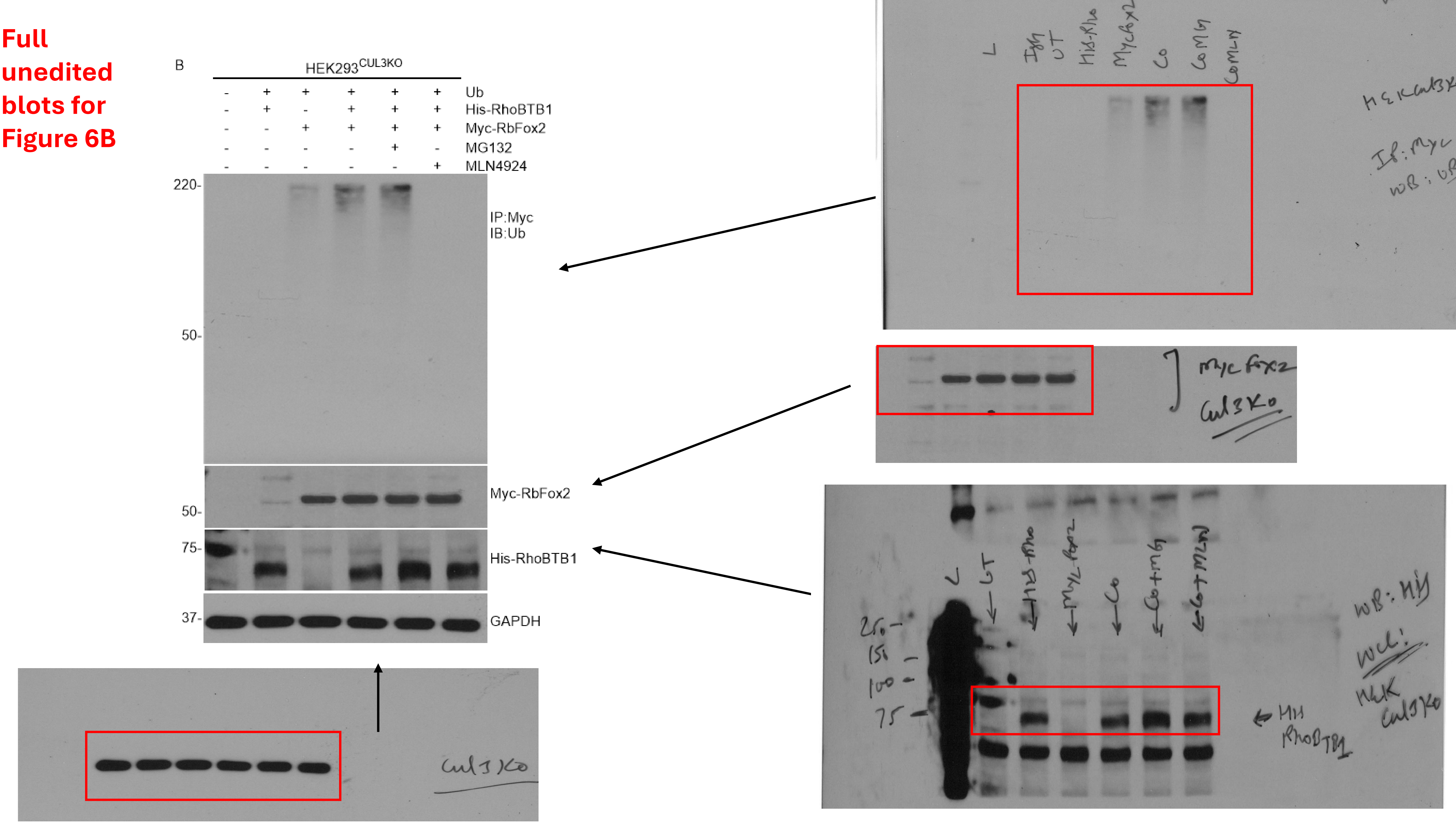

# Full unedited blots for Figure 7A

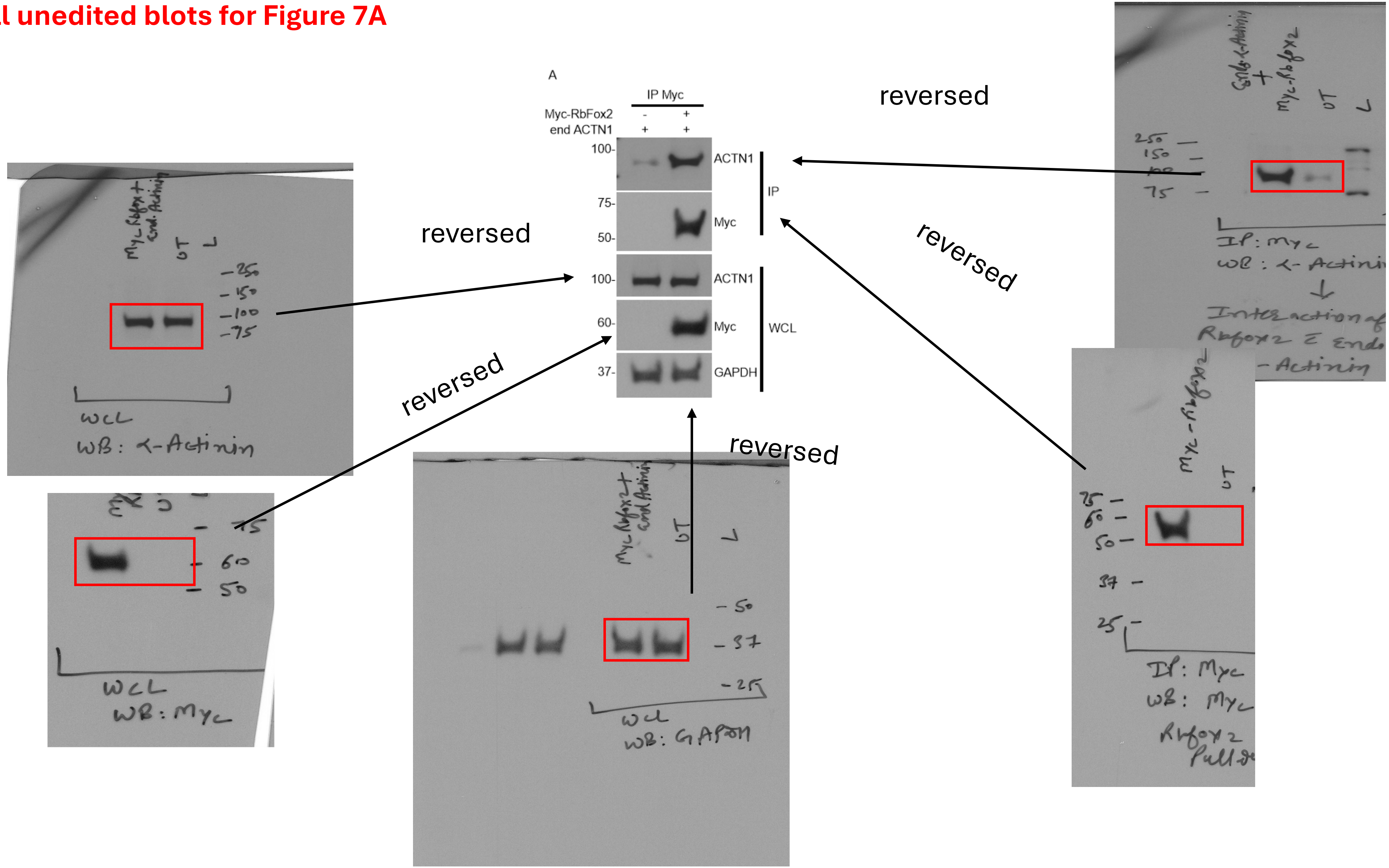

# Full unedited blots for Figure 7B

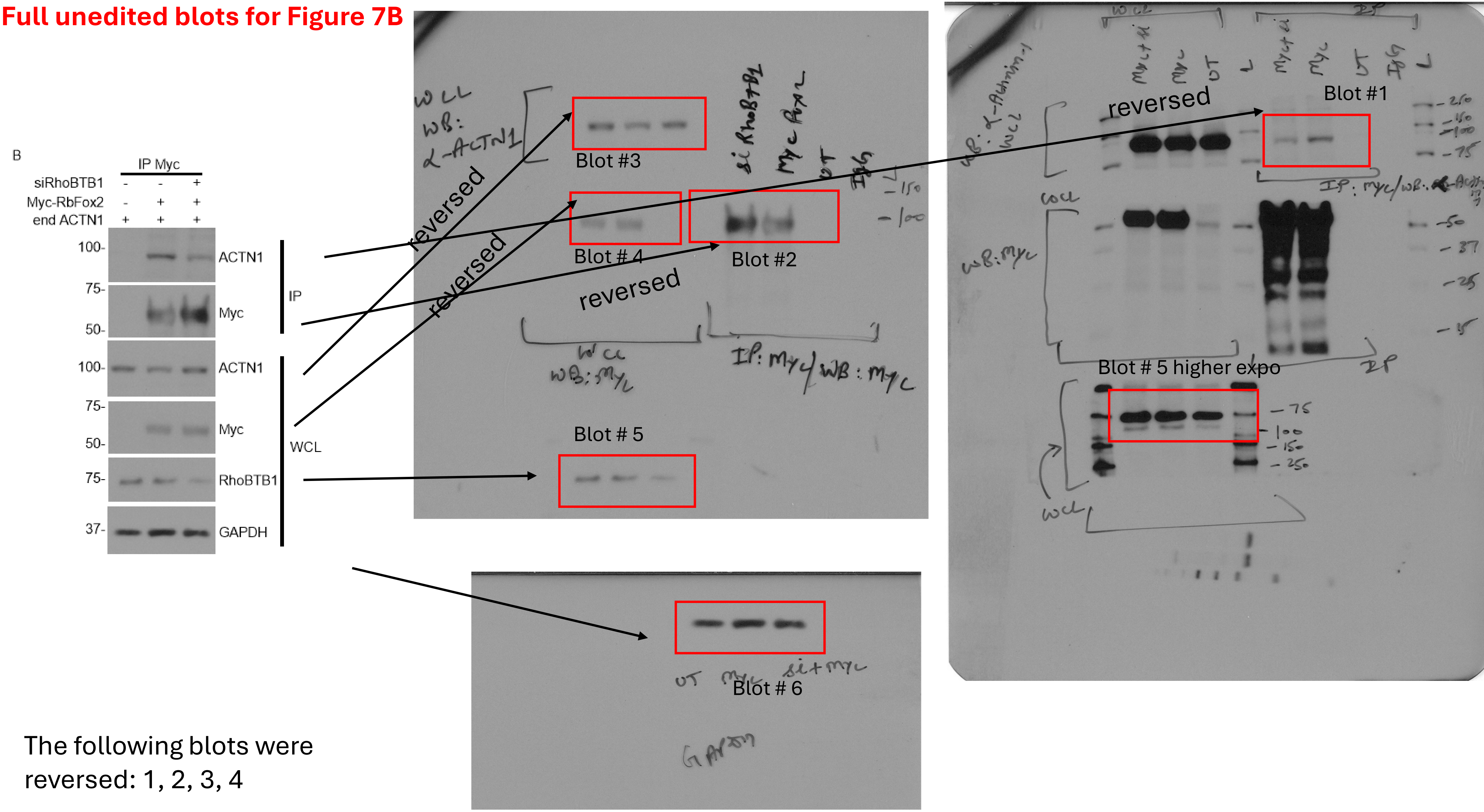


The following blots were reversed: 1, 2, 3, 4

# Full unedited blots for Figure 10B

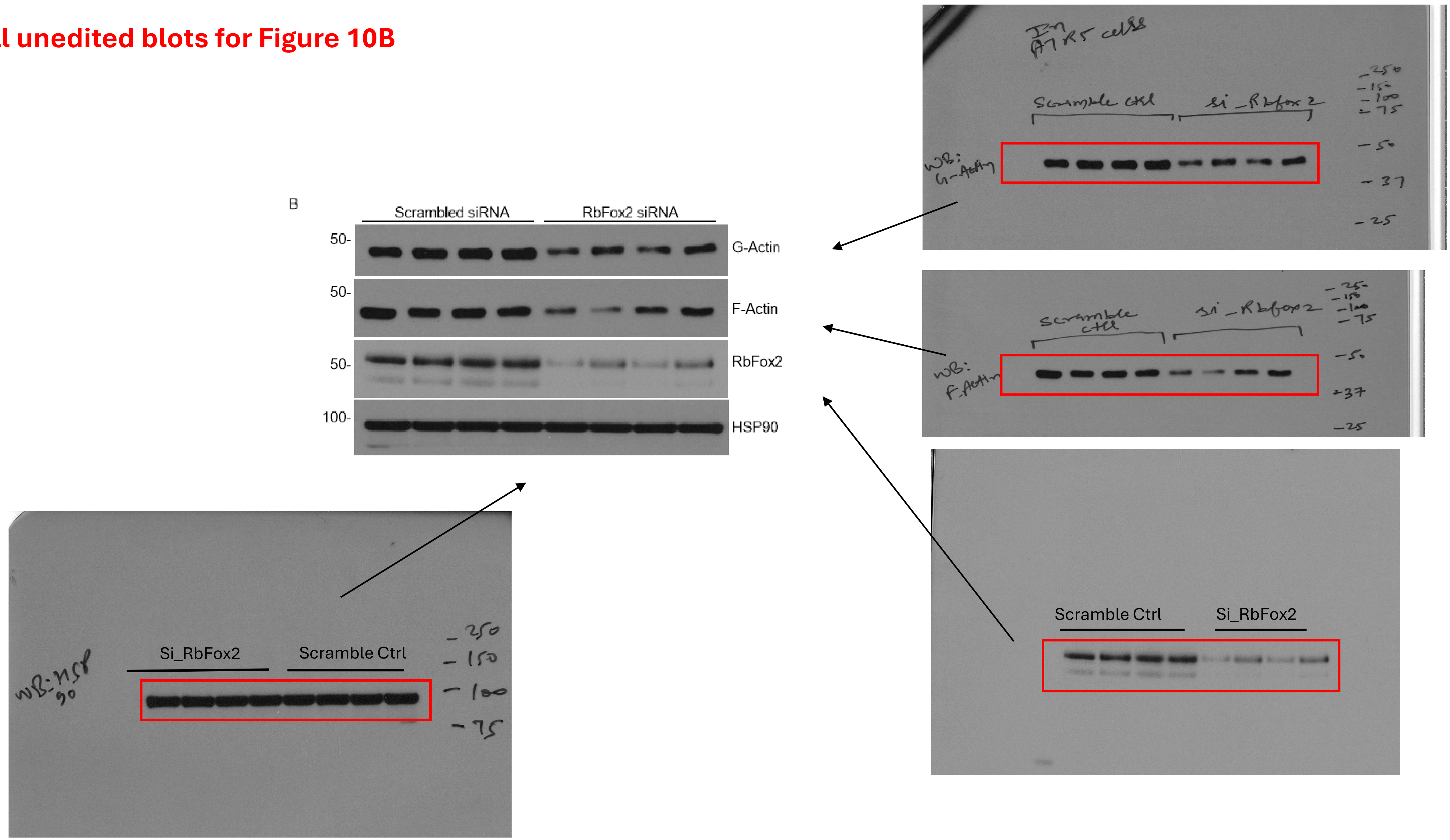

## Full unedited blots for Figure 11A

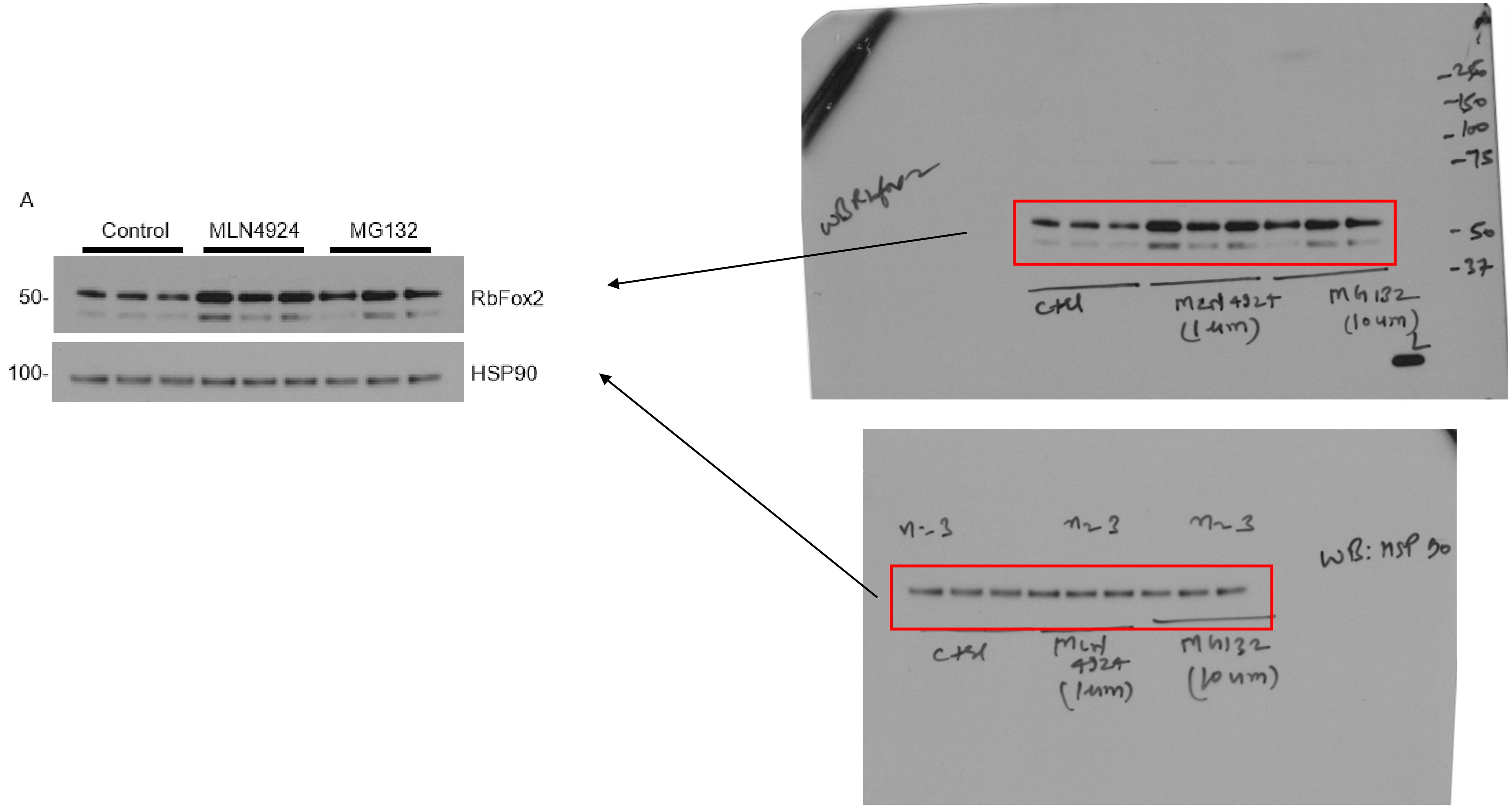

# Full unedited blots for Figure 11B

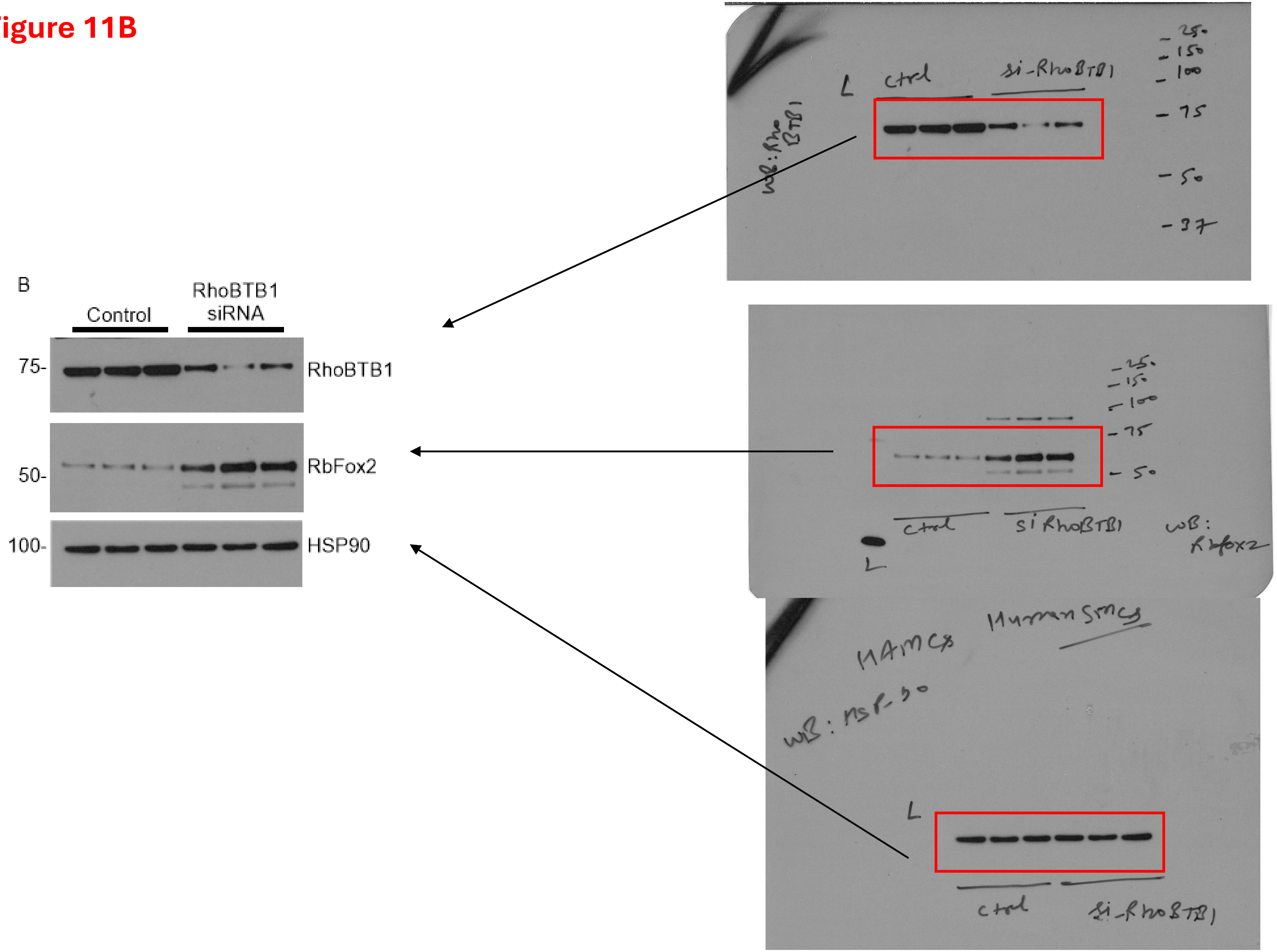

# Full unedited blots for Figure 11C

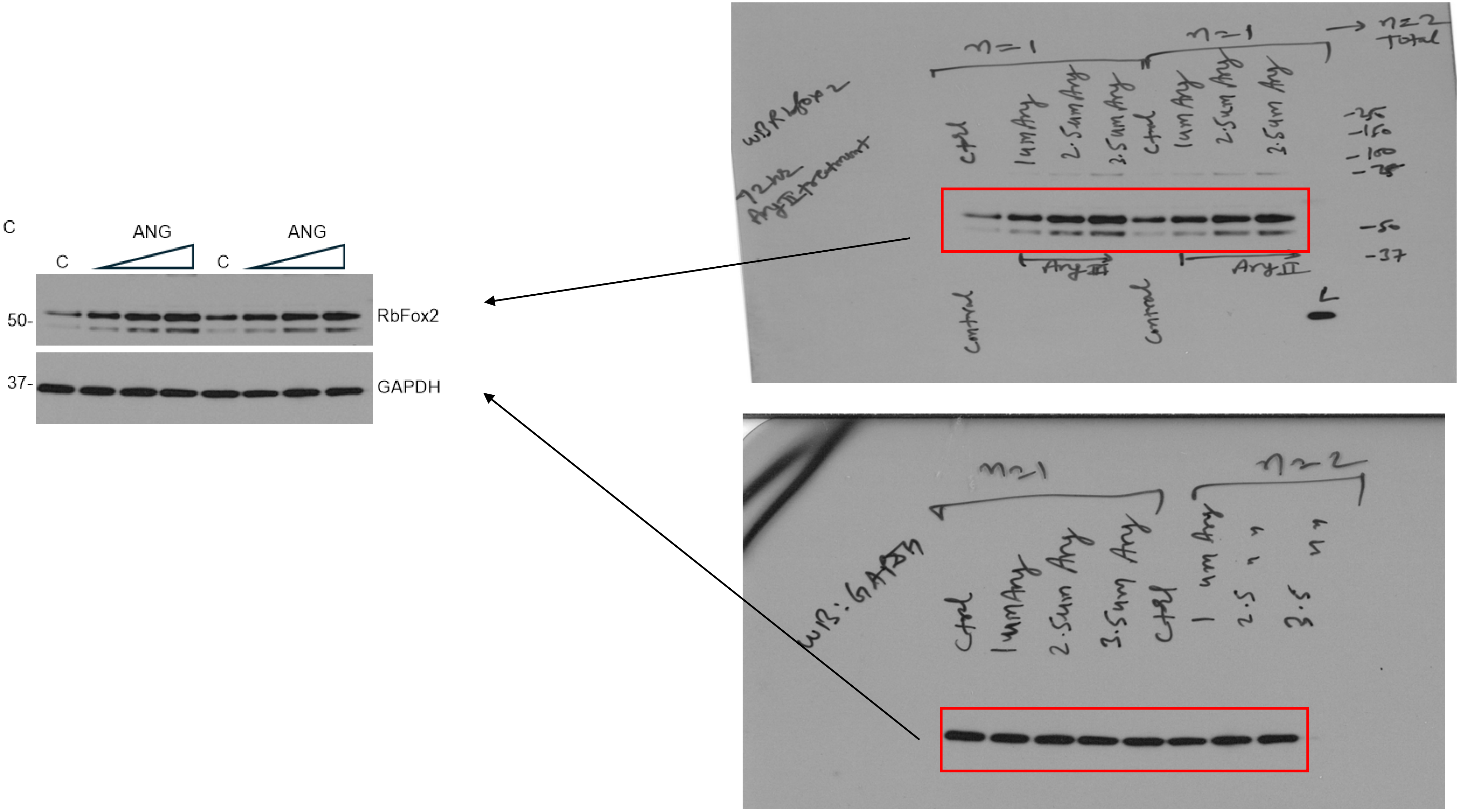

# Full unedited blots for Figure S2

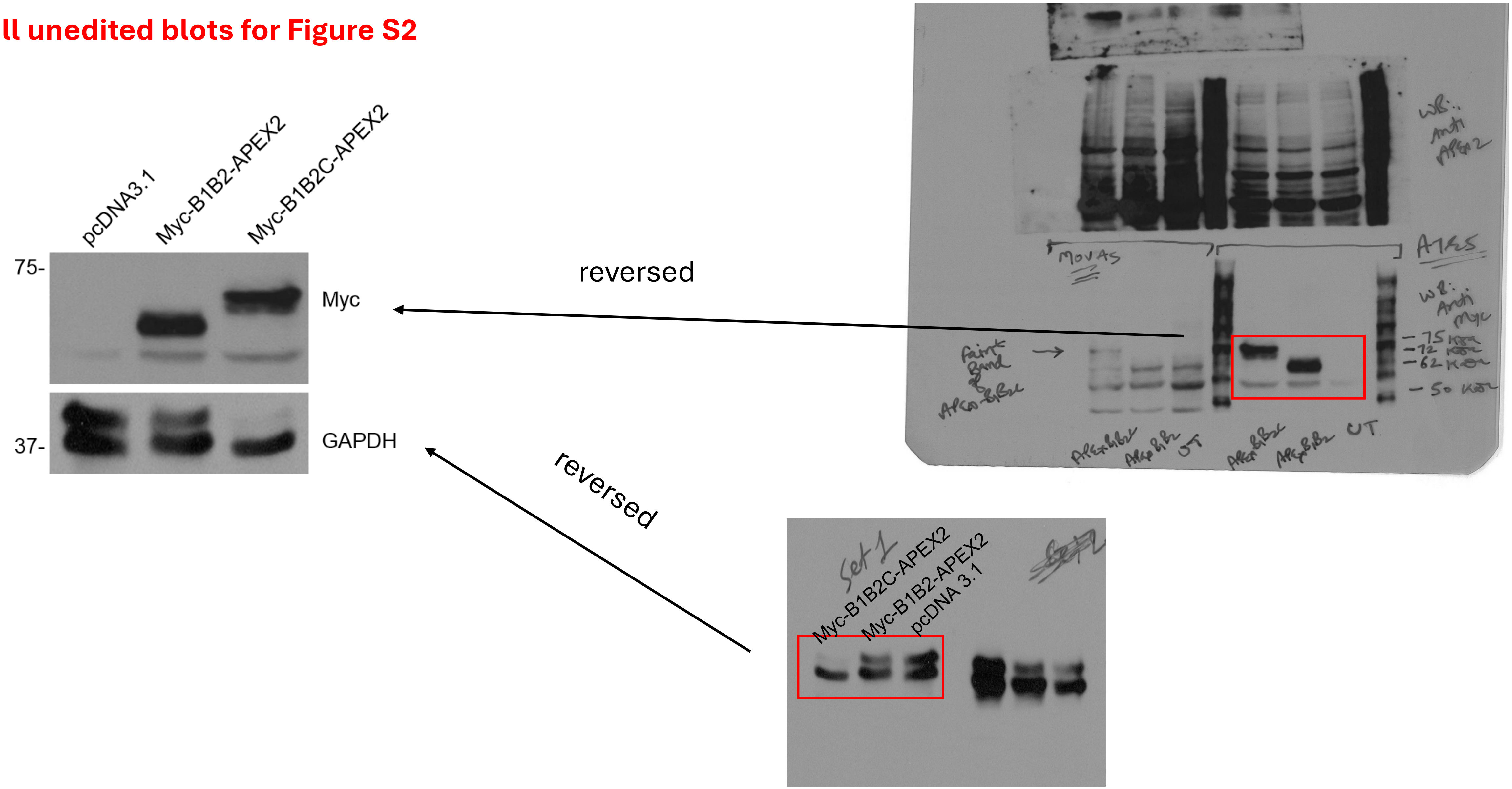

# Full unedited blots for Figure S3

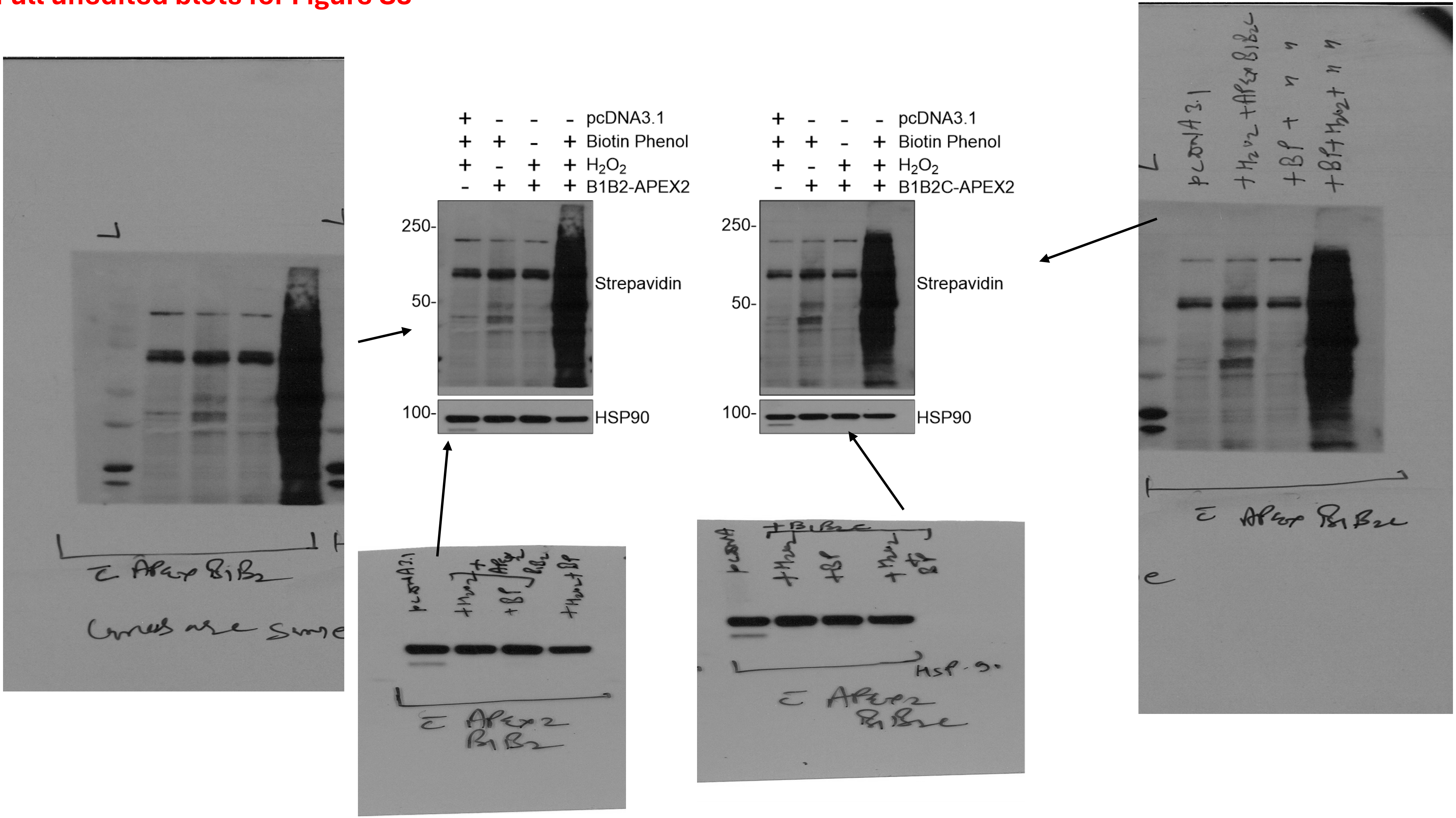

# Full unedited blots for Figure S5A

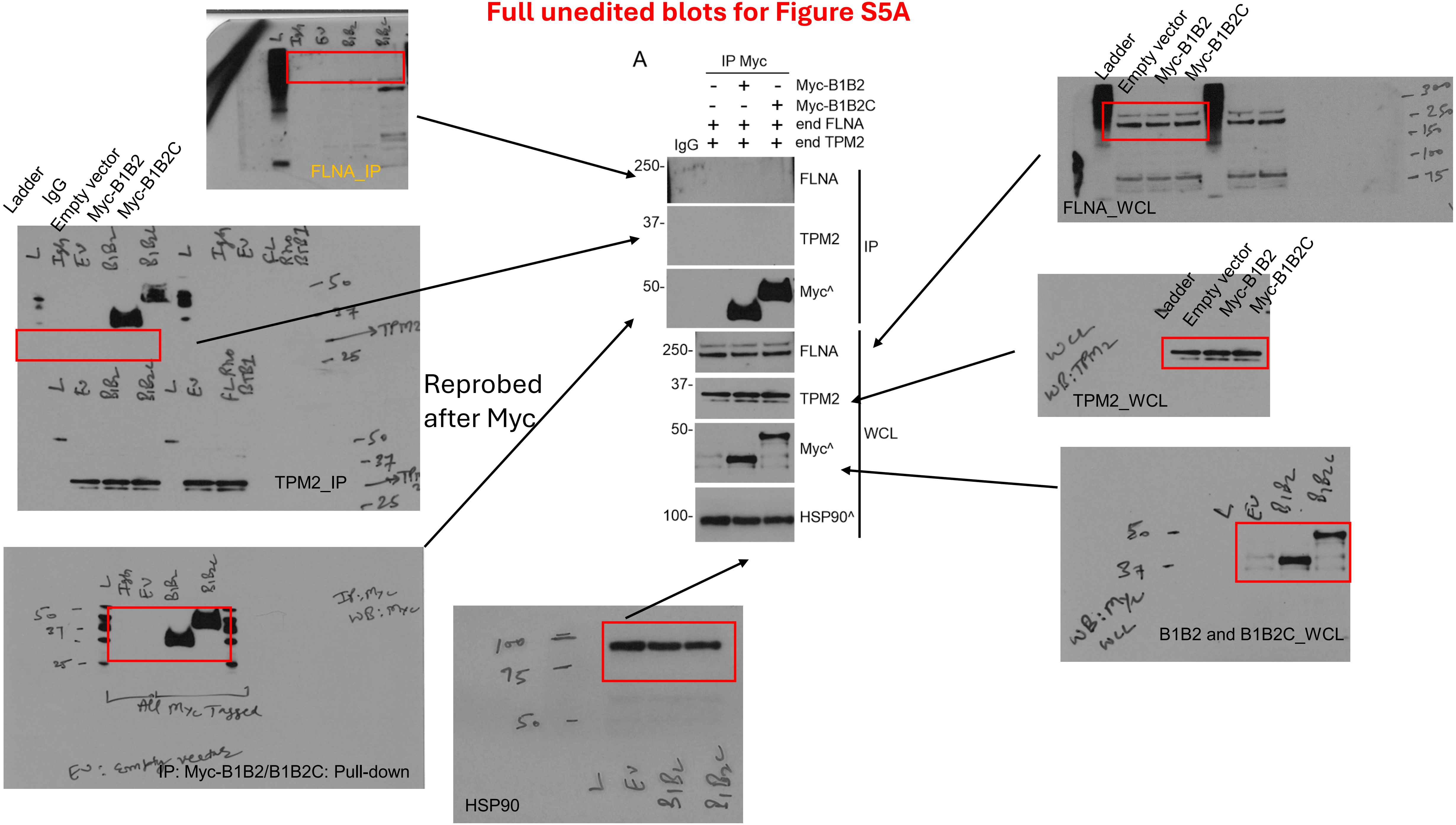

# Full unedited blots for Figure S5B

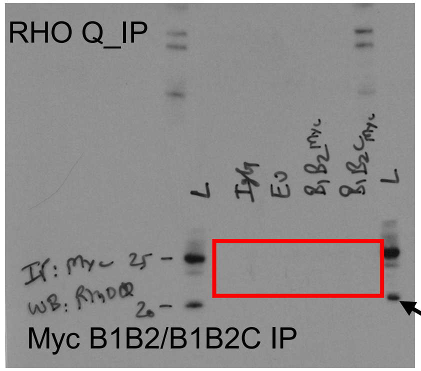


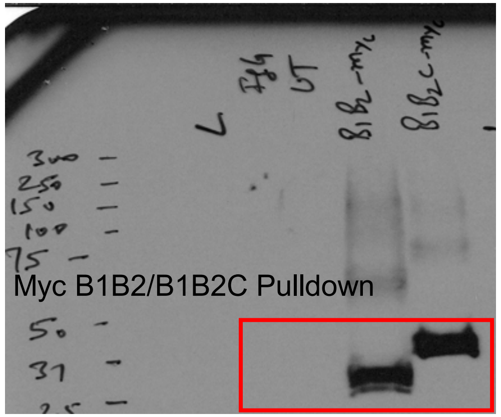


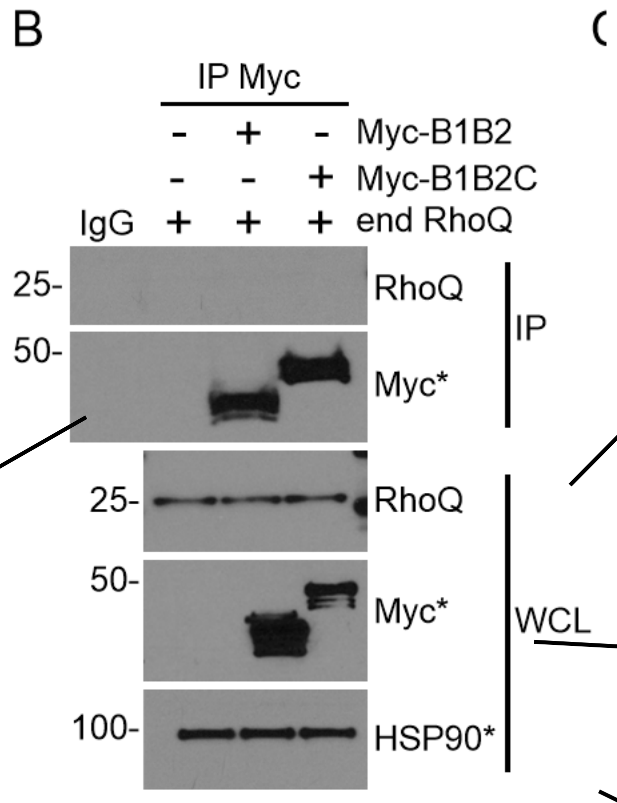


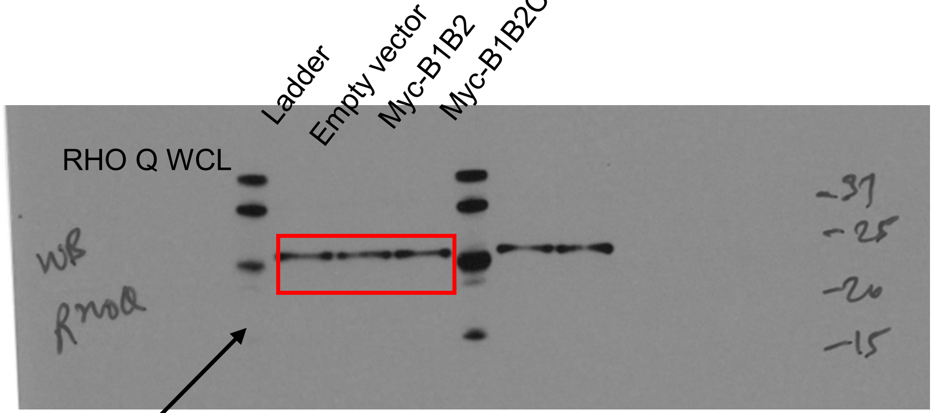


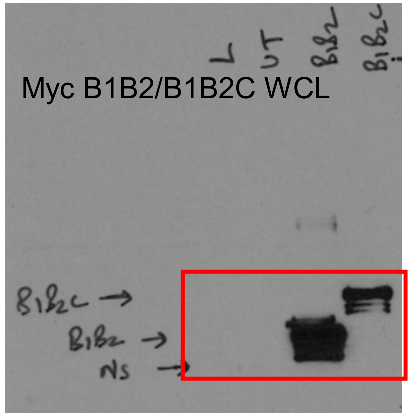


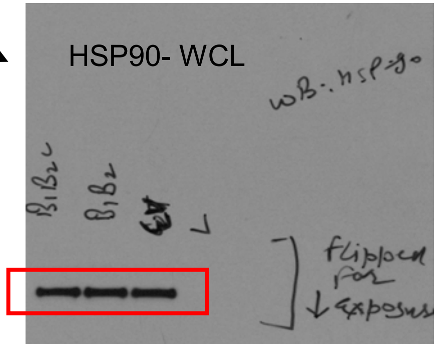

# Full unedited blots for Figure S5C

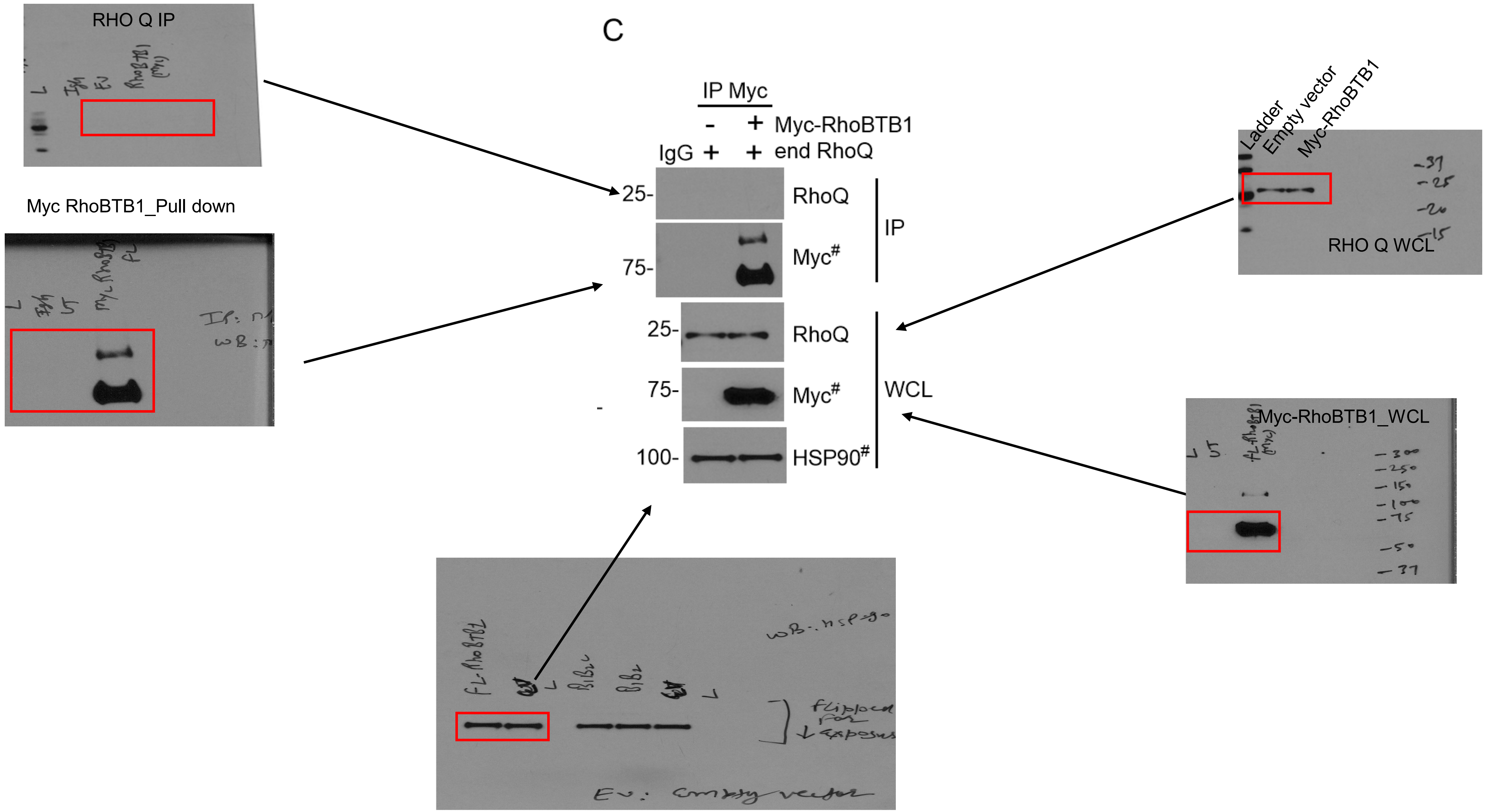

Full unedited gel for Figure S12

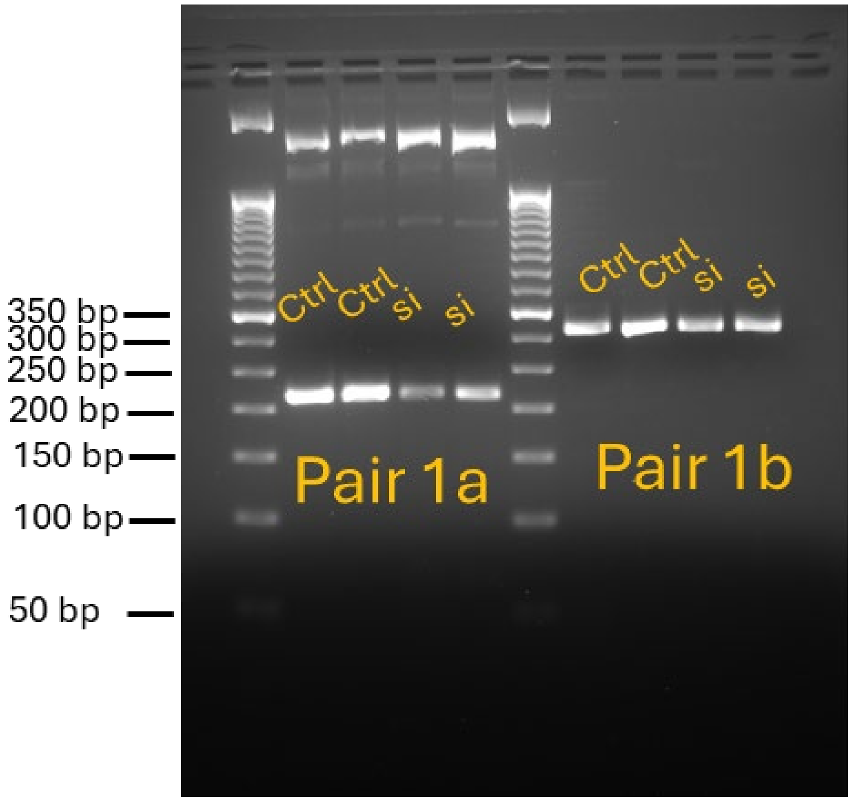


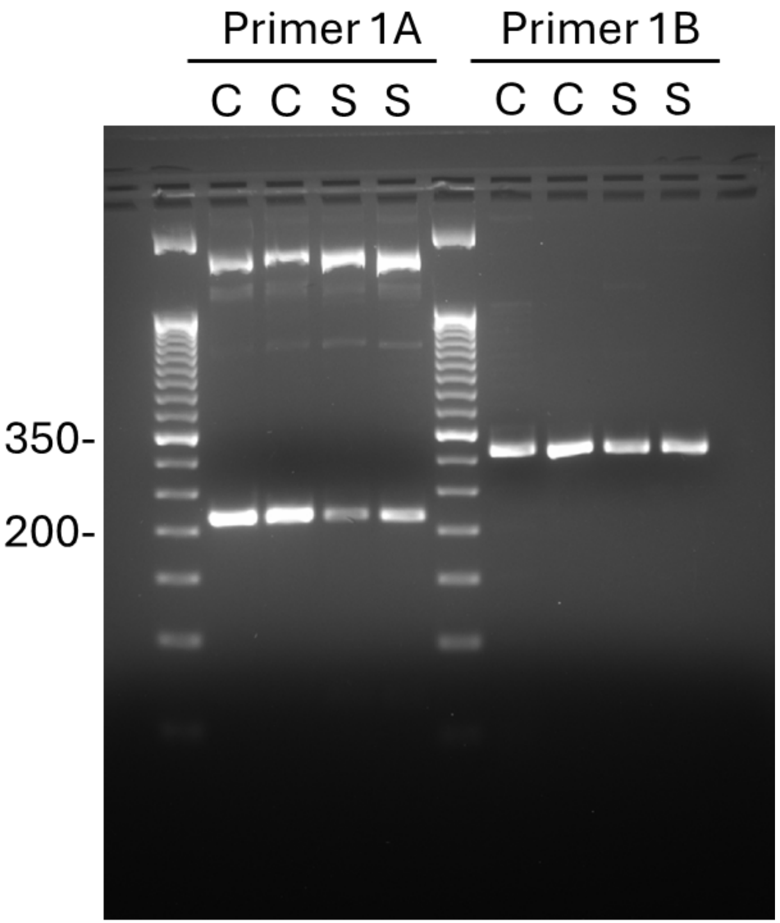


C=Control
S=Rbfox2 siRNA

# Full unedited blots for Figure S13

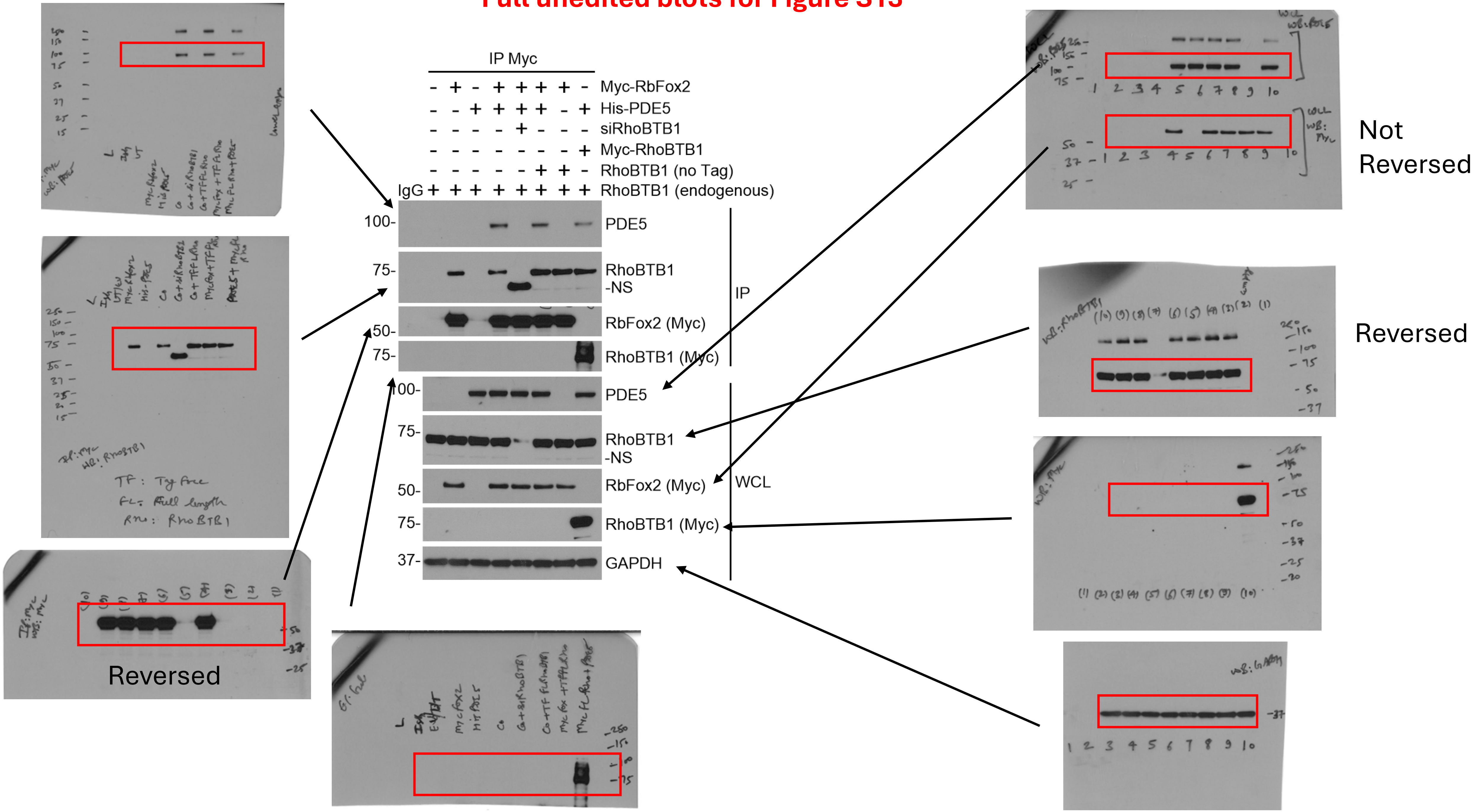

Supplement: Unedited blot and gel images [file jciinsight-11-202638-s104.pdf]
